# Supplementary material for: Structural and functional basis of mammalian microRNA biogenesis by Dicer
Source: Mol Cell. 2022 Nov 3;82(21):4064–4079.e13. doi: 10.1016/j.molcel.2022.10.010 (PMC9645528; doi:10.1016/j.molcel.2022.10.010)
Supplement: Document S1. Figures S1–S7, Tables S2–S5, and supplemental references [file mmc1.pdf]

Molecular Cell, Volume *82*

Supplemental information

Structural and functional basis of mammalian microRNA biogenesis by Dicer

David Zapletal, Eliska Taborska, Josef Pasulka, Radek Malik, Karel Kubicek, Martina Zanova, Christian Much, Marek Sebesta, Valeria Buccheri, Filip Horvat, Irena Jenickova, Michaela Prochazkova, Jan Prochazka, Matyas Pinkas, Jiri Novacek, Diego F. Joseph, Radislav Sedlacek, Carrie Bernecky, Dónal O'Carroll, Richard Stefl, and Petr Svoboda

Supplemental information

Supplemental information includes the following material:

Figures

Figure S1 - Production and validation of Dicer mouse mutants.

Figure S2 - Phenotype of *Dicer*$^{\Delta HEL1/\Delta HEL1}$ mouse mutants.

Figure S3 - miRNome dysregulation in *Dicer*$^{\Delta HEL1/\Delta HEL1}$ mouse mutants

Figure S4 - Purification and cryo-EM analyses of apo-Dicer.

Figure S5 - Purification and cryo-EM analyses of Dicer–pre-miR-15a complex.

Figure S6 - Reconstitution and cryo-EM analyses of Dicer^{O}–pre-miR-15a complex.

Figure S7 - Reconstitution and cryo-EM analysis of Dicer–pre-miR-15a–TARBP2 complex.

Tables

Table S2 - Expression of host genes of most upregulated mirtrons in ESCs

Table S3 - Asymmetric cleavage of miRNAs

Table S4 - Cryo-EM data collection and refinement statistics

Table S5 - RNA-seq libraries

Table S1 - miRNA expression in mutants is provided in a non-PDF format separately

Supplementary Figures

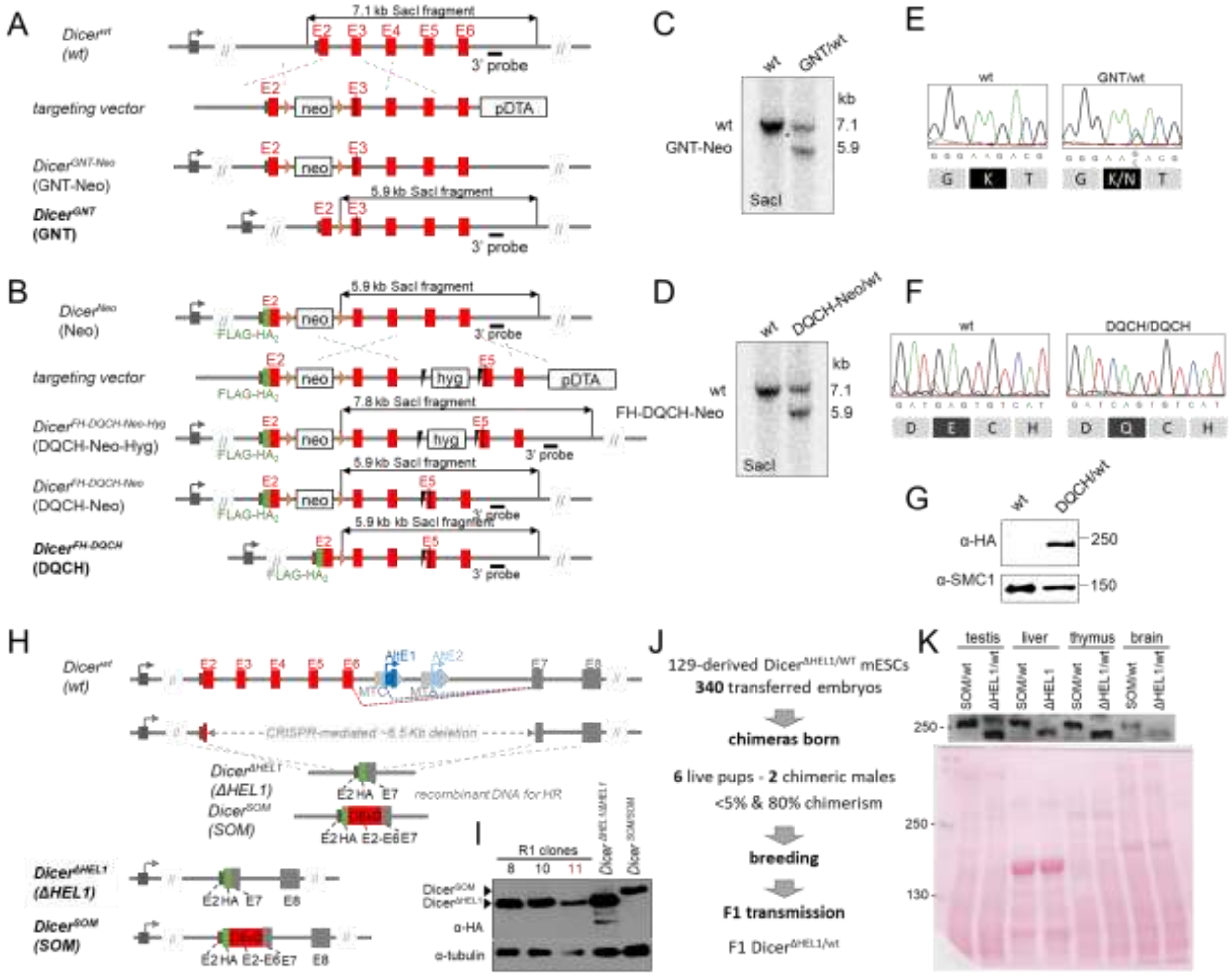

Figure S1 **Production and validation of Dicer mouse mutants.** Related to Figure 1A. (**A, B**) Schematic depiction of introduction of the GNT and the DQCH mutation into endogenous *Dicer* gene, respectively. (**C**) Detection of the GNT and (**D**) DQCH alleles by Southern blotting. (**E, F**) Validation of the mutated alleles by Sanger sequencing. (**G**) Western blot demonstrating expression of the mutated $Dicer^{DQCH}$ protein. (**H**) Schematic depiction of engineering of $Dicer^{\Delta HEL1}$ (ΔHEL1) and $Dicer^{SOM}$ (SOM) in the genomic sequence encoding HEL1 of the endogenous *Dicer* gene. Briefly, a fragment from exon 2 to exon 7 was removed using CRISPR /Cas9 and recombined with a $Dicer^{\Delta HEL1}$ recombination construct carrying exon 2 (5' UTR and start codon), HA-tag, and exon 7 coding sequence. $Dicer^{SOM}$, which was produced using the same strategy, was described previously[1]. Both alleles were validated by sequencing. (**I**) Western blotting of selected positive clones using anti-HA antibody, tubulin was used as a loading control. The heterozygous line 11 gave rise to $Dicer^{\Delta HEL1}$ mice. (**J**) Outline of the $Dicer^{\Delta HEL1}$ mouse strain production process. (**K**) Western blot analysis of $Dicer^{\Delta HEL1}$ expression in different tissues of a heterozygote $Dicer^{\Delta HEL1/wt}$ mouse. Tissues from $Dicer^{SOM/wt}$ mouse were used for comparison. 80 µg of total protein lysate were loaded per lane. Ponceau staining of the membrane shown below provides control for equal loading.

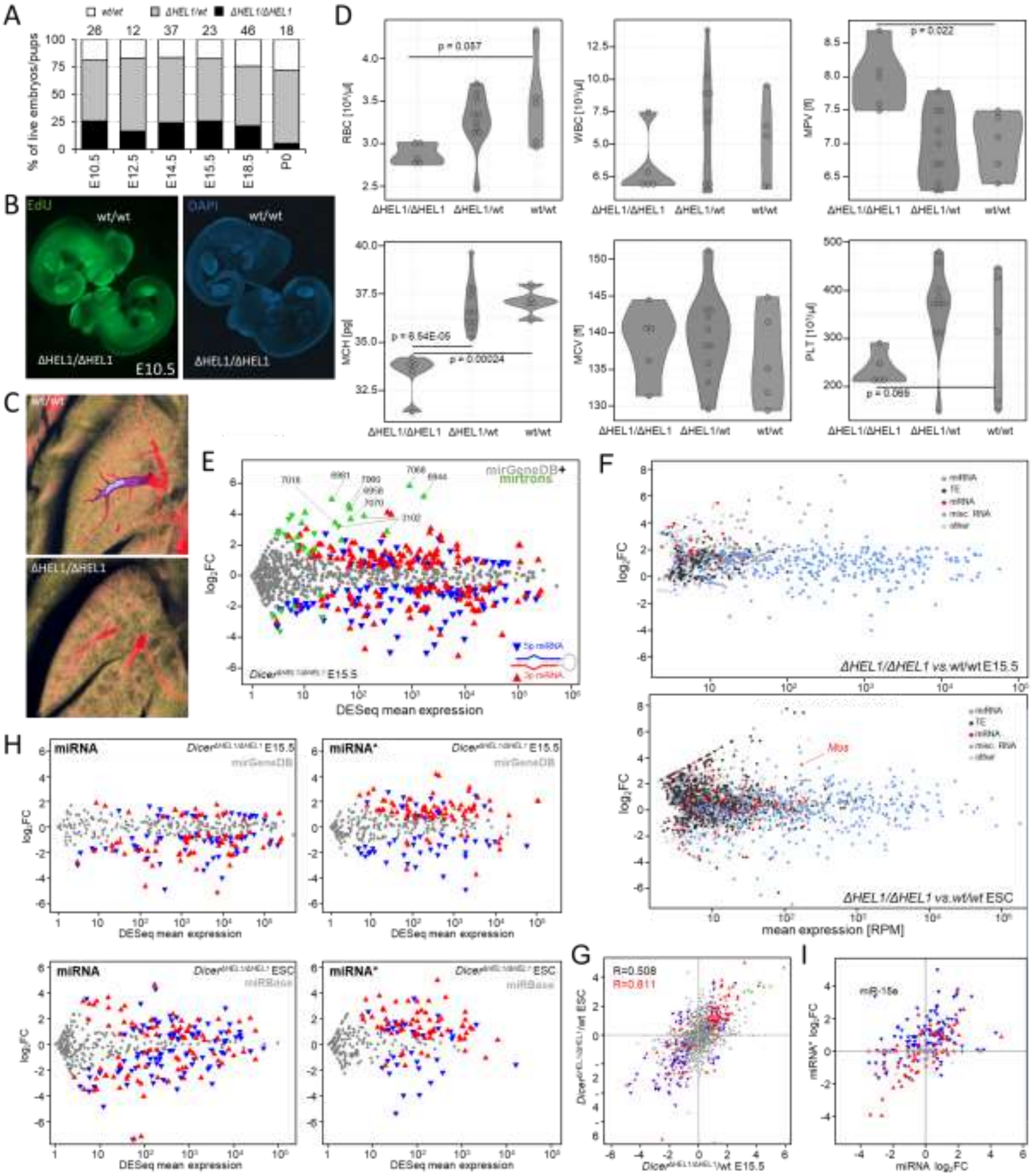

Figure S2 **Phenotype of *Dicer*$^{\Delta HEL1/\Delta HEL1}$ mouse mutants.** Related to Figure 1 and 2 (**A**) Genotype segregation in embryos from *Dicer*$^{\Delta HEL1/wt}$ parents. *Dicer*$^{\Delta HEL1/\Delta HEL1}$ embryos survive until birth but die soon after. (**B**) A combined EdU and DAPI staining of E10.5 embryos shows no apparent difference in general proliferation pattern suggesting more specific mechanism beyond the growth retardation phenotype. The experiment was performed twice with the same result. (**C**) MicroCT scans of lungs from E18.5 embryos. False coloring reveals reduced airway development and branching (red and purple colors) in *Dicer*$^{\Delta HEL1/\Delta HEL1}$ embryos. For each genotype a section with the highest signal was selected in corresponding anatomical positions. Scale bar = 5mm. (**D**) Defects in hematopoiesis found in *Dicer*$^{\Delta HEL1/\Delta HEL1}$ mice. Shown are: red blood cell count per µl (RBC, reduced by 17%), mean red blood cell volume (MCV), mean hemoglobin per cell (MCH, reduced by 10%), platelet count per µl (PLT), mean platelet volume (MPV), and white blood cell count (WBC) per µl. (**E**) Differential expression of miRNAs is not an artifact of using miRBase annotationa. MA plot shows differentially-expressed high-confidence annotated murine miRNAs from mirGeneDB database [2], which reveals highly similar miRNA dysregulation as shown in Fig. 2H except of mirtrons, which are not included in the mirGeneDB and had to be analyzed separately using mirtron annotation by Ladewig et al. [3]. Significantly

dysregulated 5p and 3p miRNAs (DESeq p-value 0.05) are shown as oriented blue ▼ and red ▲ triangles, respectively. Mirtrons are represented by green triangles whose orientation is the same as that of significantly dysregulated 5p and 3p miRNAs. Three embryos with the wild type and five with the mutant genotype were used. (**F**) Effect of $Dicer^{\Delta HEL1}$ expression on small RNAs in E15.5 embryos and in ESCs. Each MA plot shows results of small RNA-seq analysis of a *Dicer*$^{\Delta HEL1/\Delta HEL1}$ sample compared with the normal control (wild-type siblings or the parental ESC line). Each colored point represents a genomic region (cluster) producing 21-23 nt RNAs. Clusters were identified and categorized by a previously developed algorithm [4]. Cluster "expression" is defined as a fraction of small RNA reads mapping to it per million 21-23 nt small RNAs (RPM). (**G**) Relative changes of significantly differentially expressed miRNAs in *Dicer*$^{\Delta HEL1/\Delta HEL1}$ E15.5 embryos (shown as colored triangles, other miRNAs are depicted as grey circles) correlate with changes of these miRNAs in *Dicer*$^{\Delta HEL1/\Delta HEL1}$ ESCs. Axes depict log_2FC. (**H**) Unique 3p passenger strand bias in *Dicer*$^{\Delta HEL1/\Delta HEL1}$ samples (upper two MA plots) or embryonic stem cell mutants (lower two MA plots) is reproducible when using a mirGeneDB miRNA annotation. MA plots depict relative changes of dominant miRNAs (left) and passenger strands (miRNA*, right) in *Dicer*$^{\Delta HEL1/\Delta HEL1}$ mutants. 5p and 3p origins of significantly changed miRNAs or miRNA*s are distinguished by color and triangle orientation as depicted. (**I**) Relative changes of dominant miRNAs and their passenger strands in *Dicer*$^{\Delta HEL1/\Delta HEL1}$ ESCs. Each triangle depicts the strand (5p or 3p) of the dominant miRNA, its position corresponds to relative changes of the dominant miRNA (x-axis) and its corresponding miRNA* (y-axis). Deep color indicates significantly dysregulated miRNAs.

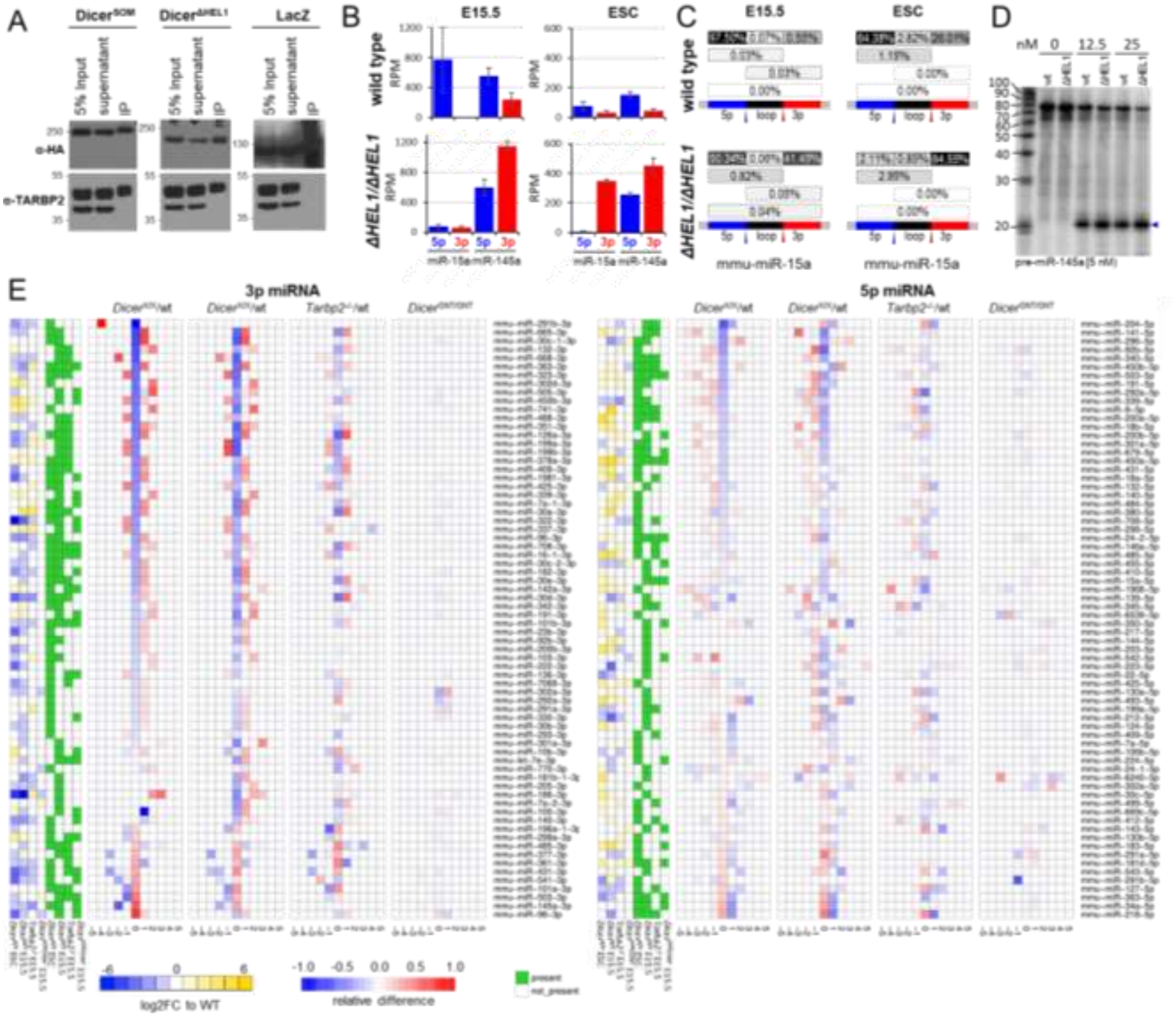

Figure S3 miRNome dysregulation in *Dicer*$^{\Delta HEL1/\Delta HEL1}$ mouse mutants. Related to Figure 3. (**A**) TARBP2 binds Dicer$^{\Delta HEL1}$. The western blots show TARBP2 presence in immunoprecipitates of Dicer^{SOM} and Dicer$^{\Delta HEL1}$ isoforms. HA-tagged Dicer^{SOM} or Dicer$^{\Delta HEL1}$ transiently expressed in NIH 3T3 cells were immunoprecipitated with α–HA antibody, and were analyzed by western blotting. The lower band in bottom western blots is a TARBP2 isoform, which does not interact with Dicer. (**B**) Expression of miR-15a and miR-145a miRNAs in *Dicer*$^{\Delta HEL1/\Delta HEL1}$ and *Tarbp2*$^{-/-}$ mutants. Expression is shown in reads per million (RPM) of 19-25 nt RNA fragments. Error bars = SD. (**C**) Products of asymmetric cleavage of pre-miR-15a are detectable in RNA-seq data from E15.5 and ESC samples. The blue-black-red lines at the bottom of each panel represent genomic pre-miRNA sequence with 5p miRNA, loop and 3p miRNA. Dicer cleavage positions are depicted by blue (3' end of 5p miRNA) and red (5' end of 3p miRNA) arrowheads. Above are shown RNA fragments corresponding to pre-miRNA, mature miRNAs, the loop, and fragments cleaved only at the 3' of 5p miRNA or 5' of 3p miRNA. Numbers correspond to percentages observed in RNA-seq data from ESCs. (**D**) miR-145a *in vitro* cleavage assay. 5 nM of *in vitro* synthesized P^{32} 5'-end labeled pre-miRNA were incubated with indicated concentrations of recombinant Dicer variants at 37°C for 60 minutes, resolved by PAGE, and visualized by phosphorimaging. Blue and red arrowheads point to products corresponding to cleavage sites giving rise to 5p and 3p miRNA, respectively. (**E**) Cleavage fidelity in *Dicer*$^{\Delta HEL1/\Delta HEL1}$ mouse mutants. Heatmaps depict analysis of cleavage sites at the 5' end of 3p miRNAs (left heatmap) and 3' end of 5p miRNAs (right heatmap) in 50 most affected miRNAs among all miRNAs (>100 DESeq RPMs) in Dicer$^{\Delta HEL1/\Delta HEL1}$ E15.5 embryos and ESCs. At the center is the annotated cleavage site. Each column of squares represents one nucleotide from the cleavage site in direction into the mature miRNA (to the right) or upstream of it (to the left). Red-blue colors indicate relative changes in a miRNA cleavage site relative to the wild type sample.

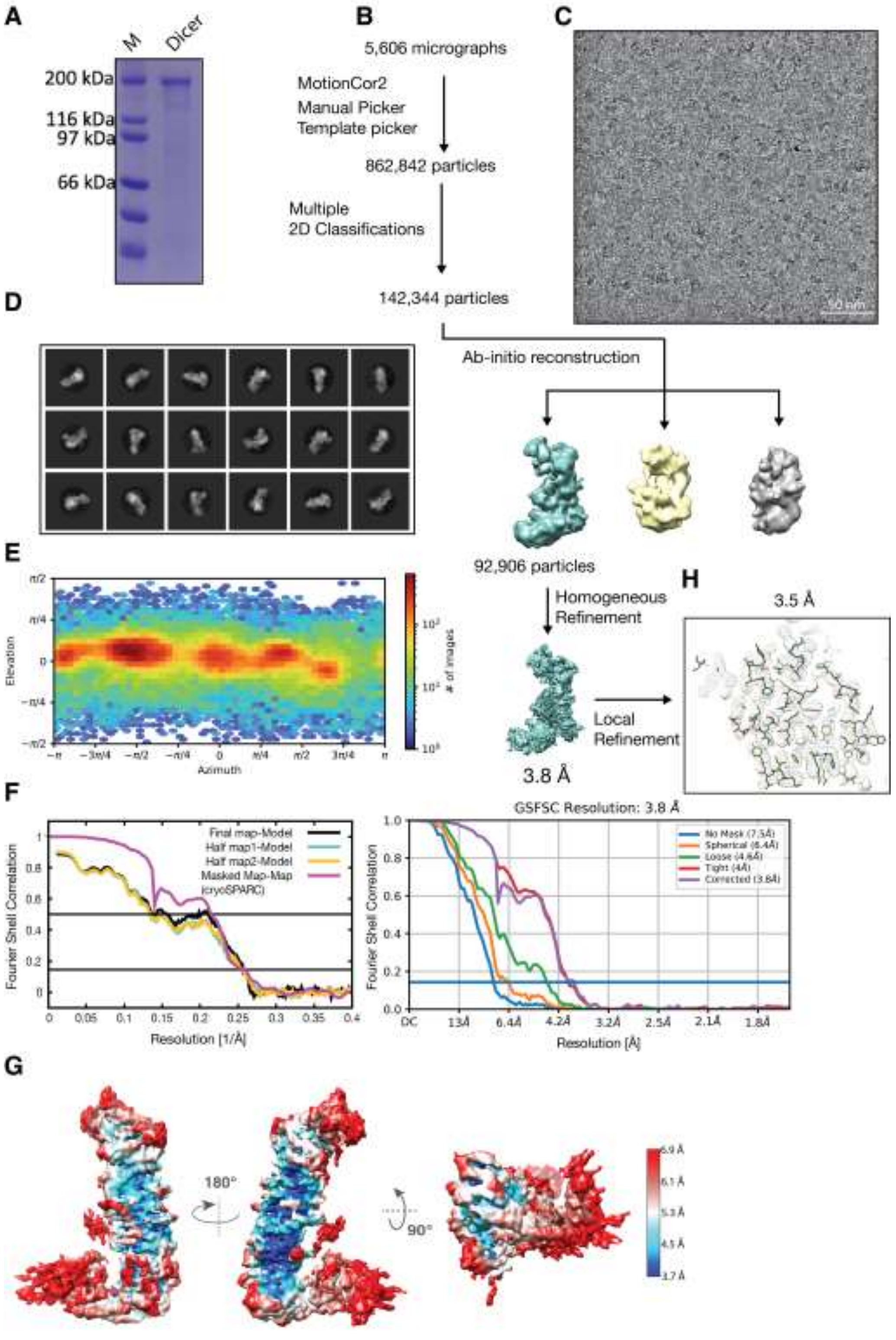

Figure S4 **Purification and cryo-EM analyses of apo-Dicer.** Related to Figure 4B (**A**) SDS-page analysis of apo-Dicer. (**B**) Outline of the image processing steps used to obtain the 3.8-Å-resolution cryo-EM reconstruction of apo-Dicer. 3D classes with no density of the helicase domain (due to inherent flexibility) were not used for the final reconstruction. (**C**) Representative Cryo-EM micrograph of apo-Dicer. (**D**) Gallery of reference-free 2D class averages. (**E**) Heat map for distribution of particles for the final 3D reconstruction. (**F**) Final map FSC (magenta) and map-to-model FSC for the full map (black), half map 1 (cyan), half map 2 (gold) curves (left). FSC curves and resolutions calculated in cryoSPARC during final refinement before and after applying soft masks (right). (**G**) Local resolution map of the final 3D reconstruction. (**H**) cryo-EM map from local refinement and fitting of coordinates.

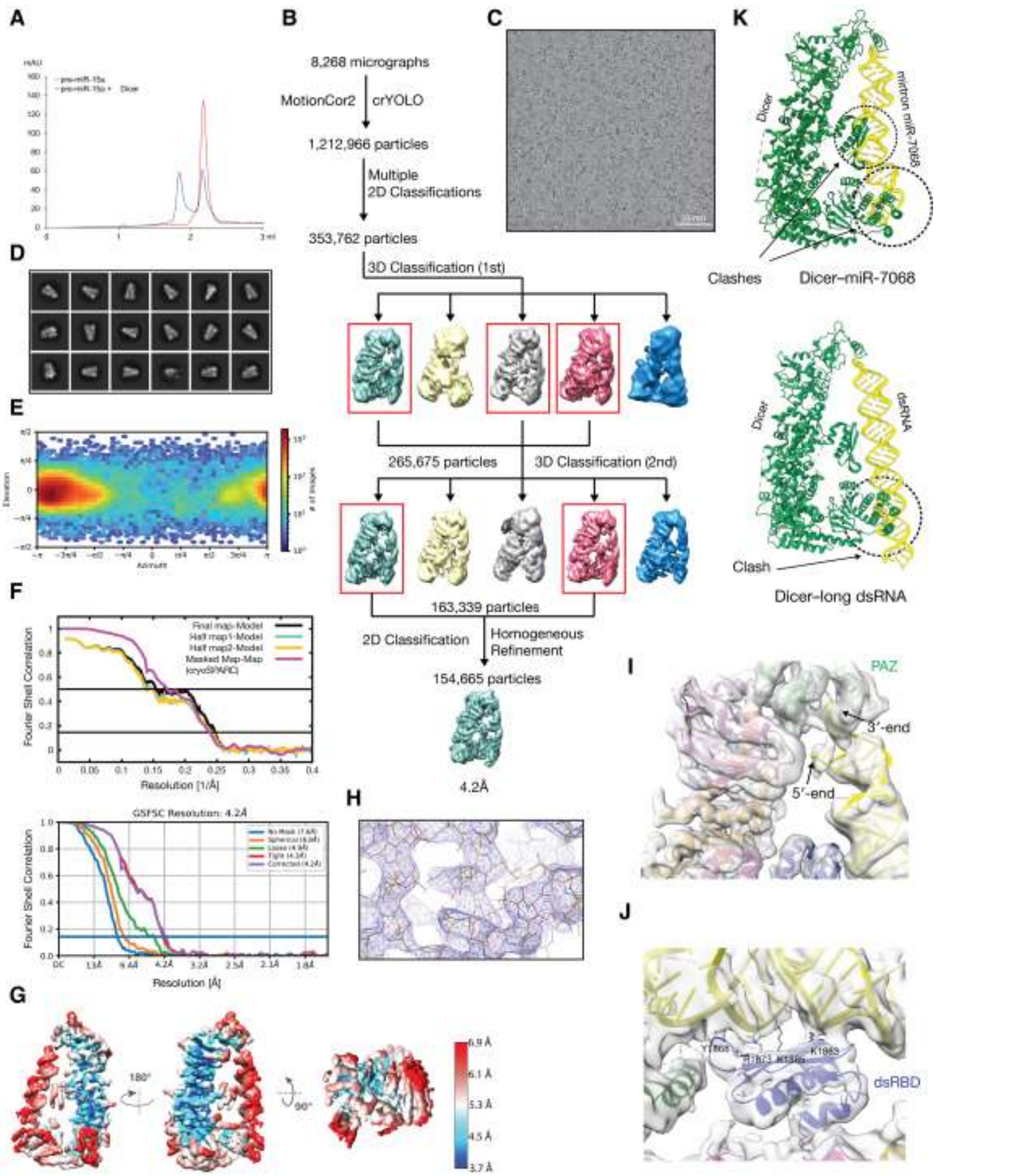

Figure S5 **Purification and cryo-EM analyses of Dicer–pre-miR-15a complex.** Related to Figure 4C (**A**) Gel filtration analysis of Dicer–pre-miR-15a complex. (**B**) Outline of the image processing steps used to obtain the 4.2-Å-resolution cryo-EM reconstruction of the Dicer–pre-miR-15a complex. (**C**) Representative cryo-EM micrograph of the Dicer–pre-miR-15a complex. (**D**) Gallery of reference-free 2D class averages. (**E**) Heat map for distribution of particles for the final 3D reconstruction. (**F**) Final map FSC (magenta) and map-to-model FSC for the full map (black), half map 1 (cyan), half map 2 (gold) curves (top). FSC curves and resolutions calculated in cryoSPARC during final refinement before and after applying soft masks (bottom) (**G**) Local resolution map of the final 3D reconstruction. (**H**) cryo-EM map and fitting of coordinates. (**I**) A close-up of the 3' end of the pre-miR-15a substrate recognition by the PAZ domain of Dicer. (**J**) A close-up of Dicer dsRBD–pre-miR-15a interface in the pre-cleavage state (putative interacting residues are indicated). (**K**) Dicer in the pre-cleavage state cannot optimally bind long mirtrons or dsRNA due to steric hindrance (indicated by arrows). The models are build based of the cryo-EM structure of Dicer–pre-miR-15a complex, in which pre-miR-15a was replaced by miR-7068 (top) or a 42-bp dsRNA (bottom).

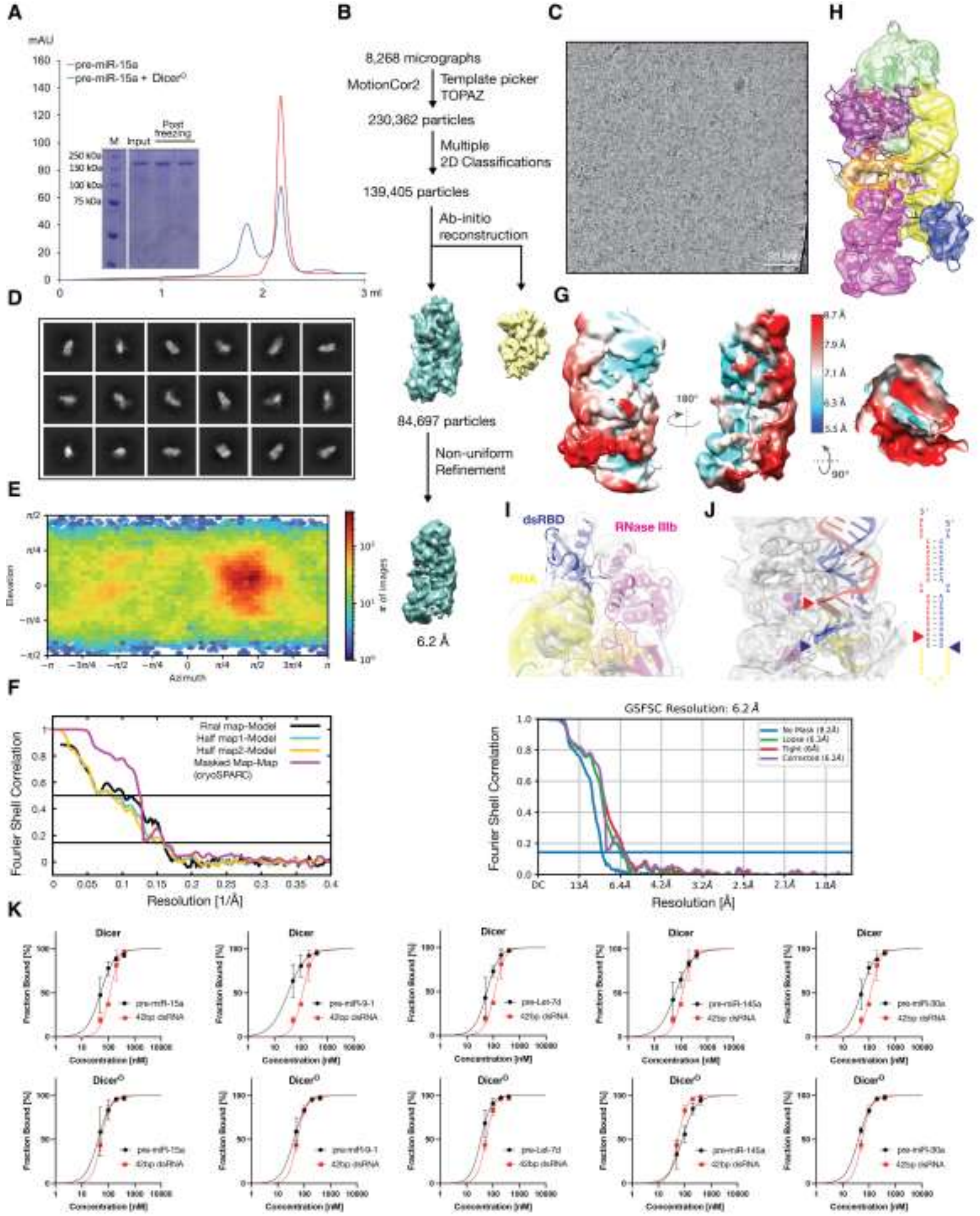

Figure S6 **Reconstitution and cryo-EM analyses of Dicer^{O}–pre-miR-15a complex.** Related to Figure 5. (**A**) Gel filtration and SDS analyses of Dicer^{O}–pre-miR-15a complex. (**B**) Outline of the image processing steps used to obtain the 6.2-Å-resolution cryo-EM reconstruction of the Dicer^{O}–pre-miR-15a complex. (**C**) Representative cryo-EM micrograph. (**D**) Gallery of reference-free 2D class averages. I Heat map for distribution of particles for the final 3D reconstruction. (**F**) Final map FSC (magenta) and map-to-model FSC for the full map (black), half map 1 (cyan), half map 2 (gold) curves (left). FSC curves and resolutions calculated in cryoSPARC during final refinement before and after applying soft masks (right). (**G**) Local resolution map of the final 3D reconstruction. (**H**) Structural models superposed to segmented cryo-EM densities and for the PAZ (green), Platform (violet), Ruler (red), RNase IIIa (orange), and RNase IIIb (magenta) domains of Dicer^{O} and pre-miR-15a (yellow) are shown. (**I**) A close-up of dsRBD/RNase IIIb–RNA interface. (**J**) A close-up of putative RNA cleavage sites (indicated by arrows) and their alignment with Dicer's catalytic sites (in magenta). (**K**) Quantification of electrophoretic mobility shift assays of Dicer isoforms with different mi-RNA precursors and a 42bp perfect hairpin. Data points, mean ± SD (n=2-3).

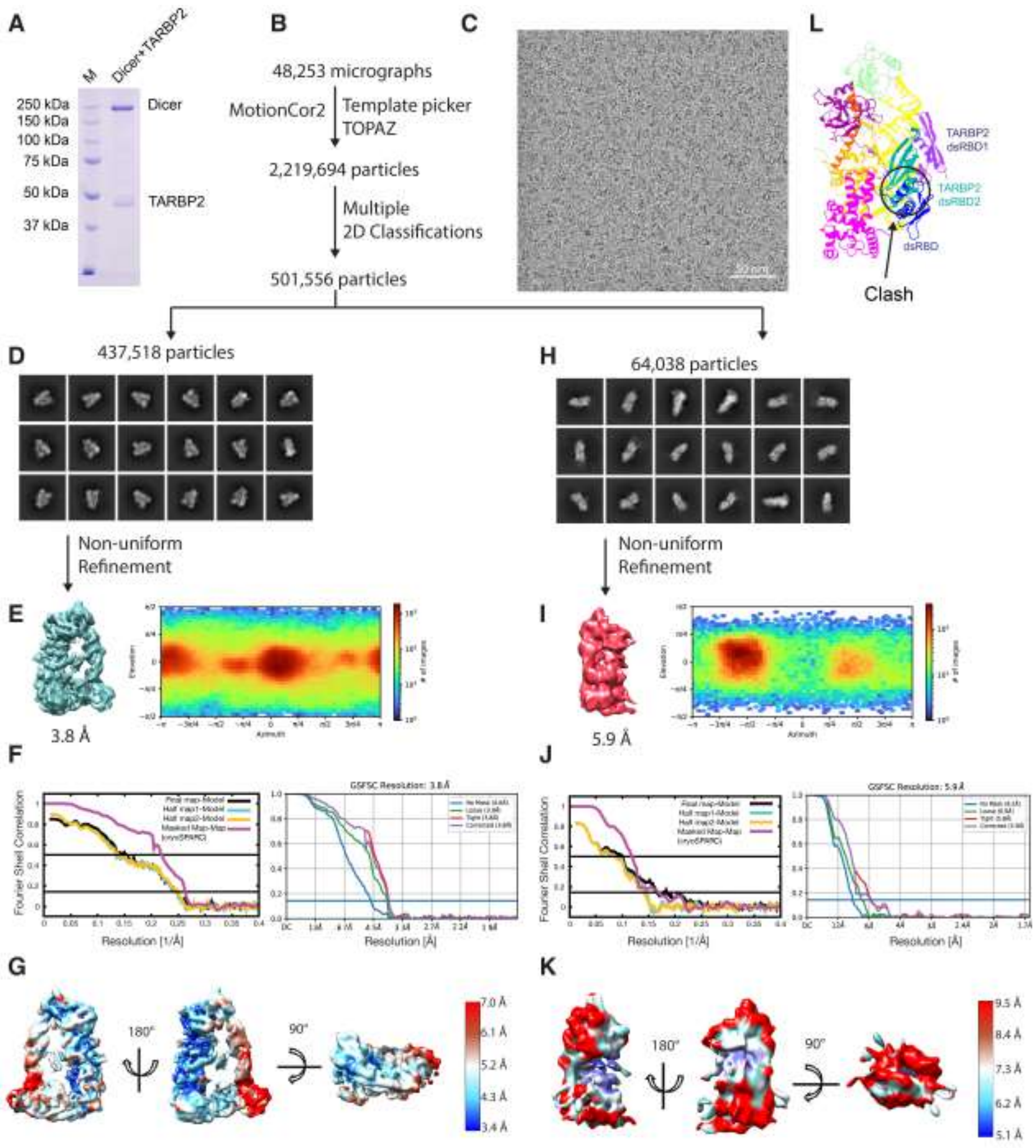

Figure S7 Reconstitution and cryo-EM analysis of Dicer–pre-miR-15a–TARBP2 complex. Related to Figure 6. (**A**) SDS-page analysis of Dicer–pre-miR-15a–TARBP2 complex. (**B**) Outline of the image processing steps of the Dicer–pre-miR-15a–TARBP2 complex. (**C**) Representative cryo-EM micrograph of the ternary complex. (**D**) Gallery of 2D class averages for the pre-cleavage state. (**E**) 3.8-Å-resolution cryo-EM reconstruction of the pre-cleavage state (left). Heat map for distribution of particles for the final 3D reconstruction (right) (**F**) Final map FSC (magenta) and map-to-model FSC for the full map (black), half map 1 (cyan), half map 2 (gold) curves (left). FSC curves and resolutions calculated in cryoSPARC during final refinement before and after applying soft masks (right). (**G**) Local resolution map of the final 3D reconstruction. (**H**) Gallery of 2D class averages for the cleavage state. (**I**) 5.9-Å-resolution cryo-EM reconstruction of the cleavage state (left). Heat map for distribution of particles for the final 3D reconstruction (right). (**J**) Final map FSC (magenta) and map-to-model FSC for the full map (black), half map 1 (cyan), half map 2 (gold) curves (left). FSC curves and resolutions calculated in cryoSPARC during final refinement before and after applying soft masks (right). (**K**) Local resolution map of the final 3D reconstruction. (**L**) Superimposition of pre-miR-15a with TARBP2 dsRBD12 from the pre-cleavage state on the Dicer–pre-miR-15a–TARBP2 structure in the cleavage state.

SUPPLEMENTARY TABLES

Table S2 Expression of host genes of most upregulated mirtrons in ESCs. Related to Figure 2.

host gene	host gene id	mRNA baseMean	log2FC	pvalue	padj	mirtron
Cherp	ENSMUSG00000052488.7	617.8	0.845	0.004	0.377	mmu-miR-7068
Dbn1	ENSMUSG00000034675.17	52.0	-0.304	0.542	1.000	mmu-miR-6944
Arap3	ENSMUSG00000024451.8	10.4	0.614	0.358	1.000	mmu-miR-6981
Fbrs	ENSMUSG00000042423.9	197.3	0.219	0.520	1.000	mmu-miR-7060
Dennd6b	ENSMUSG00000015377.9	163.9	0.105	0.791	1.000	mmu-miR-6958
Gfra4	ENSMUSG00000027316.15	11.4	-0.720	0.280	1.000	mmu-miR-6973b
Fbxw9	ENSMUSG00000008167.14	711.2	0.223	0.536	1.000	mmu-miR-7070
Hspg2	ENSMUSG00000028763.17	2418.0	-0.348	0.264	1.000	mmu-miR-7018
Nav1	ENSMUSG00000009418.15	451.2	0.807	0.006	0.499	mmu-miR-1231
Atp2b4	ENSMUSG00000026463.17	275.5	-1.178	0.003	0.351	mmu-miR-6903
Arhgef17	ENSMUSG00000032875.8	301.1	-0.549	0.085	1.000	mmu-miR-3102
Etfb	ENSMUSG00000004610.4	666.6	-0.477	0.121	1.000	mmu-miR-7051
Ptprs	ENSMUSG00000013236.17	602.1	0.012	0.971	1.000	mmu-miR-6977
Rrp1	ENSMUSG00000061032.9	1706.2	-0.101	0.757	1.000	mmu-miR-6907
Myh3	ENSMUSG00000020908.14	27.6	-0.389	0.473	1.000	mmu-miR-6923
Baiap3	ENSMUSG00000047507.12	4.4	-0.270	0.733	1.000	mmu-miR-3547
Ciao3	ENSMUSG00000002280.10	367.9	-0.313	0.296	1.000	mmu-miR-6966
Hip1r	ENSMUSG00000000915.15	189.2	0.241	0.499	1.000	mmu-miR-7032
Farsa	ENSMUSG00000003808.18	1774.8	0.373	0.228	1.000	mmu-miR-7069
Mst1	ENSMUSG00000032591.15	93.0	-0.334	0.458	1.000	mmu-miR-7088
Ap2a2	ENSMUSG00000002957.11	2406.3	0.011	0.972	1.000	mmu-miR-7063
Dnase1l1	ENSMUSG00000019088.13	27.5	-0.661	0.207	1.000	mmu-miR-7091

Table S3 Asymmetric cleavage of miRNAs. Related to Figure 3. Shown are frequencies of specific miRNA fragments in RNA-sequencing data from *Dicer*$^{\Delta HEL1/\Delta HEL1}$ ESCs. The fragment miR-5p+loop is produced by asymmetric cleave at the 5' end of 3p miRNA.

miRNA	miR-5p	loop	miR-3p	**miR-5p+loop**	miR-3p+loop
mmu-miR-7041	0.0000	0.0000	0.6667	**0.3333**	0.0000
mmu-miR-7067	0.0000	0.0000	0.3333	**0.3333**	0.0000
mmu-miR-667	0.5358	0.0037	0.0838	**0.3322**	0.0000
mmu-miR-31	0.5807	0.0000	0.0519	**0.2948**	0.0031
mmu-miR-29c	0.1667	0.0000	0.1667	**0.2222**	0.0000
mmu-miR-101b	0.0000	0.0000	0.8350	**0.1456**	0.0012
mmu-miR-465b	0.5317	0.0000	0.3394	**0.1106**	0.0000
mmu-miR-465b	0.5317	0.0000	0.3394	**0.1106**	0.0000
mmu-miR-539	0.2650	0.2222	0.0513	**0.0769**	0.0256
mmu-miR-154	0.2100	0.0284	0.6601	**0.0664**	0.0002
mmu-miR-3070	0.2405	0.0000	0.6881	**0.0476**	0.0000
mmu-miR-465c	0.5198	0.0000	0.4097	**0.0407**	0.0000
mmu-miR-465c	0.5198	0.0000	0.4097	**0.0407**	0.0000
mmu-miR-142a	0.3436	0.0082	0.3154	**0.0402**	0.0000
mmu-miR-377	0.5106	0.0000	0.4012	**0.0359**	0.0000
mmu-miR-367	0.0000	0.0827	0.7694	**0.0351**	0.0000
mmu-miR-878	0.6171	0.0000	0.2904	**0.0334**	0.0000
mmu-miR-293	0.2776	0.0000	0.6612	**0.0291**	0.0001
**mmu-miR-15a**	**0.0193**	**0.0078**	**0.8588**	**0.0270**	**0.0000**
mmu-miR-141	0.1689	0.1947	0.3683	**0.0264**	0.0034
mmu-miR-324	0.0620	0.0361	0.8431	**0.0250**	0.0000
mmu-miR-883b	0.5185	0.0000	0.4577	**0.0238**	0.0000
mmu-miR-376b	0.0291	0.0173	0.9008	**0.0235**	0.0000
mmu-miR-485	0.3695	0.0001	0.5528	**0.0214**	0.0146
mmu-miR-20b	0.9475	0.0006	0.0089	**0.0199**	0.0004
mmu-miR-743b	0.0394	0.0000	0.9235	**0.0185**	0.0000
mmu-miR-665	0.0400	0.0000	0.8164	**0.0176**	0.0820
mmu-miR-679	0.7761	0.0000	0.0568	**0.0167**	0.0000
mmu-miR-465a	0.0921	0.0000	0.8556	**0.0143**	0.0057
mmu-miR-181c	0.0812	0.0000	0.8520	**0.0140**	0.0000
mmu-miR-188	0.9352	0.0139	0.0000	**0.0139**	0.0000
mmu-miR-411	0.7924	0.0023	0.1631	**0.0139**	0.0000
mmu-miR-93	0.9053	0.0019	0.0396	**0.0135**	0.0000
mmu-miR-301a	0.5232	0.0067	0.1228	**0.0133**	0.0000
mmu-miR-362	0.4786	0.0000	0.4550	**0.0130**	0.0000
mmu-miR-341	0.0241	0.0000	0.8388	**0.0121**	0.0003
mmu-miR-380	0.3384	0.0000	0.5523	**0.0115**	0.0000
mmu-miR-211	0.8401	0.0000	0.1078	**0.0109**	0.0021
mmu-miR-29a	0.0085	0.0000	0.9267	**0.0096**	0.0000
mmu-miR-677	0.2121	0.0000	0.0004	**0.0089**	0.0025

Table S4 Cryo-EM data collection and refinement statistics. Related to Figure 4-6.

**Instrument**					
Microscope	FEI Titan Krios				
**Data collection**					
**Sample**	**Dicer**	**Dicer–RNA**	**Dicer^{O}–RNA**	**Dicer–RNA-TARBP2 (pre-cleavage)**	**Dicer–RNA-TARBP2 (cleavage)**
EMDB accession number	EMD-14387	EMD-14383	EMDB-14384	EMDB-14856	EMDB-14854
PDB accession number	7YZ4	7YYM	7YYN	7ZPK	7ZPI
Voltage (kV)	300	300	300	300	300
Detector (counting mode)	Gatan K2	Gatan K2	Gatan K2	Gatan K3	Gatan K3
Symmetry	C1	C1	C1	C1	C1
Electron dose (e^-/Å^2)	55.0	55.0	55.0	60.198	60.198
Defocus range (µm)	-0.8 to -3.5	-1 to -3.5	-1 to -3.5	-0.8 to -3.5	-0.8 to -3.5
Pixel size (Å)	0.828	0.828	0.828	0.835	0.835
Movies collected	6,354	16,601	9,956	48,253	48,253
Initial particles	862,842	1,212,966	230,362	2,219,694	2,219,694
Final particles	92,906	154,665	84,697	437,518	64,038
**Model composition**					
Protein residues	1234	1281	705	1513	705
**Refinement**					
Map resolution (cryoSPARC) 0.143/0.5 (Å)	3.8/4.6	4.2/5.8	6.2/7.9	3.8/4.5	5.9/8.4
Map-to-model FSC 0.143/0.5 (Å)	3.9/7.3	4.3/6.7	6.3/9.7	4.0/6.5	6.0/9.7
Combined map resolution range (Å)	3.5 – 4.2	-	-	-	-
Map sharpening *B*-factor (Å^2)	87.8	134.8	451.4	189.2	520.0
**R.m.s deviations**					
Bond lengths (Å)	0.004	0.004	0.013	0.005	0.004
Bond angles (°)	0.780	0.798	1.917	1.089	0.290
**Validation**					
MolProbity score	1.12	1.07	0.78	1.15	0.70
Clashscore	1.46	1.57	0.92	2.24	0.61
Poor rotamers (%)	0.92	0.18	0.64	0.68	0.64
**Ramachandran plot**					
Favored (%)	96.44	97.07	98.70	97.18	97.44
Allowed (%)	3.56	2.93	1.30	2.82	2.56
Disallowed (%)	0.00	0.00	0.00	0.00	0.00

Table S5 RNA-seq libraries used in the study. Related to Figure 1-3 and STAR Methods (Method Details, section Bioinformatics analyses).

stage	type	genotype	library name	note
ESC	small RNA	*Dicer*$^{wt/wt}$	s_ESC_WT+MosIR_RS7.1	transfected with MosIR
ESC	small RNA	*Dicer*$^{wt/wt}$	s_ESC_WT+MosIR_RS7.2	transfected with MosIR
ESC	small RNA	*Dicer*$^{wt/wt}$	s_ESC_WT+MosIR_RS7.3	transfected with MosIR
ESC	small RNA	*Dicer*$^{\Delta HEL1/\Delta HEL1}$	s_ESC_XHOM+MosIR_RS10.1	transfected with MosIR
ESC	small RNA	*Dicer*$^{\Delta HEL1/\Delta HEL1}$	s_ESC_XHOM+MosIR_RS10.2	transfected with MosIR
ESC	small RNA	*Dicer*$^{\Delta HEL1/\Delta HEL1}$	s_ESC_XHOM+MosIR_RS10.3	transfected with MosIR
E15.5	small RNA	*Dicer*$^{wt/wt}$	s_E15.5_WT_1	
E15.5	small RNA	*Dicer*$^{wt/wt}$	s_E15.5_WT_6	
E15.5	small RNA	*Dicer*$^{wt/wt}$	s_E15.5_WT_8B	
E15.5	small RNA	*Dicer*$^{\Delta HEL1/\Delta HEL1}$	s_E15.5_XHOM_10B_r2	
E15.5	small RNA	*Dicer*$^{\Delta HEL1/\Delta HEL1}$	s_E15.5_XHOM_2	
E15.5	small RNA	*Dicer*$^{\Delta HEL1/\Delta HEL1}$	s_E15.5_XHOM_3B	
E15.5	small RNA	*Dicer*$^{\Delta HEL1/\Delta HEL1}$	s_E15.5_XHOM_4	
E15.5	small RNA	*Dicer*$^{\Delta HEL1/\Delta HEL1}$	s_E15.5_XHOM_7B	
E15.5	small RNA	*Dicer*$^{wt/wt}$	s_E15.5_WT_11	
E15.5	small RNA	*Dicer*$^{wt/wt}$	s_E15.5_WT_14	
E15.5	small RNA	*Dicer*$^{wt/wt}$	s_E15.5_WT_16	
E15.5	small RNA	*Dicer*$^{GNT/GNT}$	s_E15.5_GNTHOM_3	
E15.5	small RNA	*Dicer*$^{GNT/GNT}$	s_E15.5_GNTHOM_4	
E15.5	small RNA	*Dicer*$^{GNT/GNT}$	s_E15.5_GNTHOM_9	
E15.5	small RNA	*Tarbp2*$^{+/+}$	SRS2781156 B6T2-65Tarbp2_WT_1	PRJNA423238 SRP127346
E15.5	small RNA	*Tarbp2*$^{+/+}$	SRS2781151 B6T2-65Tarbp2_WT_2	PRJNA423238 SRP127346
E15.5	small RNA	*Tarbp2*$^{+/+}$	SRS2781150B6T2-65Tarbp2_WT_3	PRJNA423238 SRP127346
E15.5	small RNA	*Tarbp2*$^{-/-}$	SRS2781155 B6T2-54Tarbp2_Mut_1	PRJNA423238 SRP127346
E15.5	small RNA	*Tarbp2*$^{-/-}$	SRS2781154 B6T2-60Tarbp2_Mut_2	PRJNA423238 SRP127346
E15.5	small RNA	*Tarbp2*$^{-/-}$	SRS2781157 B6T2-90Tarbp2_Mut_3	PRJNA423238 SRP127346

References

1. Taborska, E., Pasulka, J., Malik, R., Horvat, F., Jenickova, I., Jelic Matosevic, Z., and Svoboda, P. (2019). Restricted and non-essential redundancy of RNAi and piRNA pathways in mouse oocytes. PLoS Genet *15*, e1008261. 10.1371/journal.pgen.1008261.
2. Fromm, B., Hoye, E., Domanska, D., Zhong, X., Aparicio-Puerta, E., Ovchinnikov, V., Umu, S.U., Chabot, P.J., Kang, W., Aslanzadeh, M., et al. (2022). MirGeneDB 2.1: toward a complete sampling of all major animal phyla. Nucleic Acids Res *50*, D204-D210. 10.1093/nar/gkab1101.
3. Ladewig, E., Okamura, K., Flynt, A.S., Westholm, J.O., and Lai, E.C. (2012). Discovery of hundreds of mirtrons in mouse and human small RNA data. Genome Res *22*, 1634-1645. 10.1101/gr.133553.111.
4. Flemr, M., Malik, R., Franke, V., Nejepinska, J., Sedlacek, R., Vlahovicek, K., and Svoboda, P. (2013). A retrotransposon-driven dicer isoform directs endogenous small interfering RNA production in mouse oocytes. Cell *155*, 807-816. 10.1016/j.cell.2013.10.001.
