# Supplementary material for: Structural and functional basis of mammalian microRNA biogenesis by Dicer
Source: Mol Cell. 2022 Nov 3;82(21):4064–4079.e13. doi: 10.1016/j.molcel.2022.10.010 (PMC9645528; doi:10.1016/j.molcel.2022.10.010)
Supplement: Document S2. Article plus supplemental information [file mmc3.pdf]

Molecular Cell

Article

Structural and functional basis of mammalian microRNA biogenesis by Dicer

Graphical abstract

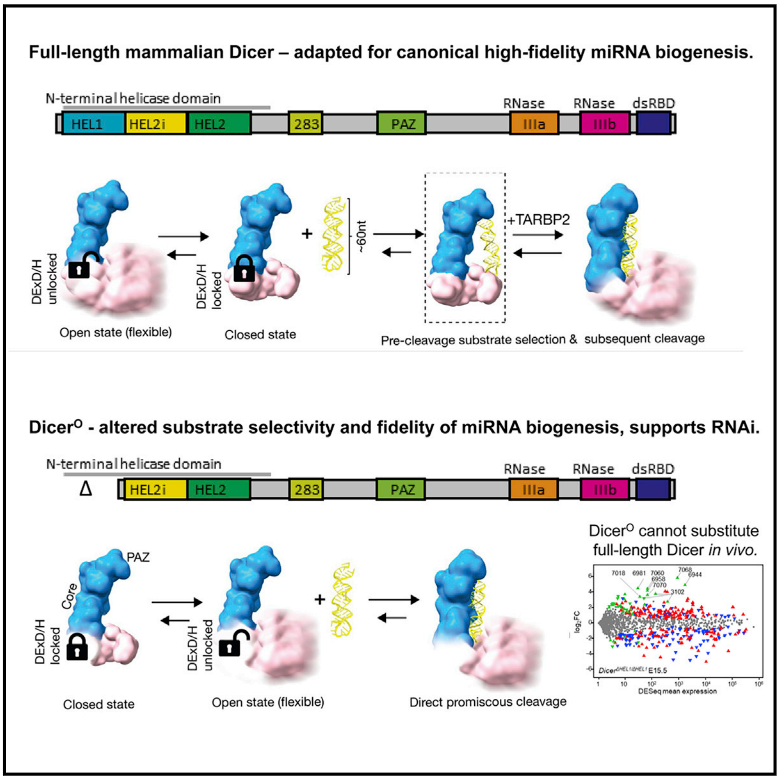

Highlights

- Structures of mouse Dicer•pre-miRNA complexes show pre-cleavage and cleavage states
- Dicer's DExD/H domain is essential in mice and has structural, not enzymatic role
- Dicer's DExD/H domain assures high fidelity of miRNA biogenesis *in vivo*
- Mammalian Dicer is committed to miRNA pathway through DExD/H-RNase IIIb interaction

Authors

David Zapletal, Eliska Taborska, Josef Pasulka, ..., Dónal O'Carroll, Richard Stefl, Petr Svoboda

Correspondence

richard.stefl@ceitec.muni.cz (R.S.), svobodap@img.cas.cz (P.S.)

In brief

Zapletal et al. provide structural and functional *in vivo* analyses of murine RNase III Dicer variants, which (1) explain how Dicer variants recognize and cleave substrates in mammalian miRNA and RNAi pathways and (2) show ATP-independent essential structural role of the DExD/H helicase domain in committing mammalian Dicer to high-fidelity miRNA biogenesis.

Zapletal et al., 2022, Molecular Cell *82*, 4064–4079
November 3, 2022 © 2022 The Author(s). Published by Elsevier Inc.
https://doi.org/10.1016/j.molcel.2022.10.010

CellPress

CellPress
OPEN ACCESS

Molecular Cell

Article

Structural and functional basis of mammalian microRNA biogenesis by Dicer

David Zapletal,[1,2,10] Eliska Taborska,[3,10] Josef Pasulka,[3,10] Radek Malik,[3] Karel Kubicek,[1,3] Martina Zanova,[1] Christian Much,[4,5] Marek Sebesta,[1] Valeria Buccheri,[3] Filip Horvat,[3,6] Irena Jenickova,[7] Michaela Prochazkova,[7] Jan Prochazka,[7] Matyas Pinkas,[1] Jiri Novacek,[1] Diego F. Joseph,[3] Radislav Sedlacek,[7] Carrie Bernecky,[8] Dónal O'Carroll,[4,5,9] Richard Stefl,[1,2,*] and Petr Svoboda[3,11,*]

[1]CEITEC-Central European Institute of Technology, Masaryk University, 625 00 Brno, Czech Republic
[2]National Centre for Biomolecular Research, Faculty of Science, Masaryk University, 625 00 Brno, Czech Republic
[3]Institute of Molecular Genetics of the Czech Academy of Sciences, v.v.i., Videnska 1083, 142 20 Prague 4, Czech Republic
[4]Centre for Regenerative Medicine, Institute for Regeneration and Repair, Institute for Stem Cell Research, School of Biological Sciences, University of Edinburgh, 5 Little France Drive, Edinburgh EH16 4UU, UK
[5]European Molecular Biology Laboratory (EMBL), Mouse Biology Unit, Via Ramarini 32, Monterotondo Scalo 00015, Italy
[6]Bioinformatics Group, Department of Biology, Faculty of Science, University of Zagreb, Horvatovac 102a, 10000 Zagreb, Croatia
[7]Czech Centre for Phenogenomics and Laboratory of Transgenic Models of Diseases, Institute of Molecular Genetics of the Czech Academy of Sciences, v.v.i., Prumyslova 595, 252 50 Vestec, Czech Republic
[8]Institute of Science and Technology Austria (ISTA), Am Campus 1, 3400 Klosterneuburg, Austria
[9]Wellcome Centre for Cell Biology, School of Biological Sciences, University of Edinburgh, Edinburgh EH9 3BF, UK
[10]These authors contributed equally
[11]Lead contact
*Correspondence: richard.stefl@ceitec.muni.cz (R.S.), svobodap@img.cas.cz (P.S.)
https://doi.org/10.1016/j.molcel.2022.10.010

SUMMARY

MicroRNA (miRNA) and RNA interference (RNAi) pathways rely on small RNAs produced by Dicer endonucleases. Mammalian Dicer primarily supports the essential gene-regulating miRNA pathway, but how it is specifically adapted to miRNA biogenesis is unknown. We show that the adaptation entails a unique structural role of Dicer's DExD/H helicase domain. Although mice tolerate loss of its putative ATPase function, the complete absence of the domain is lethal because it assures high-fidelity miRNA biogenesis. Structures of murine Dicer•–miRNA precursor complexes revealed that the DExD/H domain has a helicase-unrelated structural function. It locks Dicer in a closed state, which facilitates miRNA precursor selection. Transition to a cleavage-competent open state is stimulated by Dicer-binding protein TARBP2. Absence of the DExD/H domain or its mutations unlocks the closed state, reduces substrate selectivity, and activates RNAi. Thus, the DExD/H domain structurally contributes to mammalian miRNA biogenesis and underlies mechanistical partitioning of miRNA and RNAi pathways.

INTRODUCTION

Dicer endoribonucleases generate small RNAs for microRNA (miRNA) and RNA interference (RNAi) pathways (Paturi and Deshmukh, 2021). Both are fundamentally important eukaryotic mechanisms providing sequence-specific control of gene expression and protection against viruses and transposable elements (TEs). Although biogenesis of gene-regulating miRNAs require a single cleavage of genome-encoded small stem-loop precursors (pre-miRNA) (Bartel, 2018), RNAi entails processive cleavage of long double-stranded RNA (dsRNA) into small interfering RNAs (siRNAs) with gene-regulating or defensive roles against viruses or TEs (Ketting, 2011).

Vertebrate genomes carry a single highly conserved Dicer (*Dicer-1*) gene (Jia et al., 2017), which encodes a ~220 kDa multidomain protein that appears dedicated to the miRNA pathway. Cryoelectron microscopy (cryo-EM) of human Dicer revealed a protein architecture that resembles the letter "L," with a complex helicase domain at the base, tandem RNase III domains in the core, and Piwi/Argonaute/Zwille (PAZ)-platform domains at the cap (Lau et al., 2009, 2012; Taylor et al., 2013; Liu et al., 2018). During miRNA biogenesis, the PAZ domain, which has a strong affinity for substrates with blunt-ends or short 3′ protruding overhangs (Lingel et al., 2003; Song et al., 2003; Yan et al., 2003), binds the base of a pre-miRNA stem loop and the two RNase III domains function as catalytic "half sites," each cleaving one strand of the double-stranded substrate (Zhang et al., 2004). This yields a small RNA duplex whose length is determined by the distance of RNase III cleavage sites from the PAZ domain (MacRae et al., 2006). The helicase domain, which has a

 © 2022 The Author(s). Published by Elsevier Inc.
This is an open access article under the CC BY license (http://creativecommons.org/licenses/by/4.0/).

CellPress
OPEN ACCESS

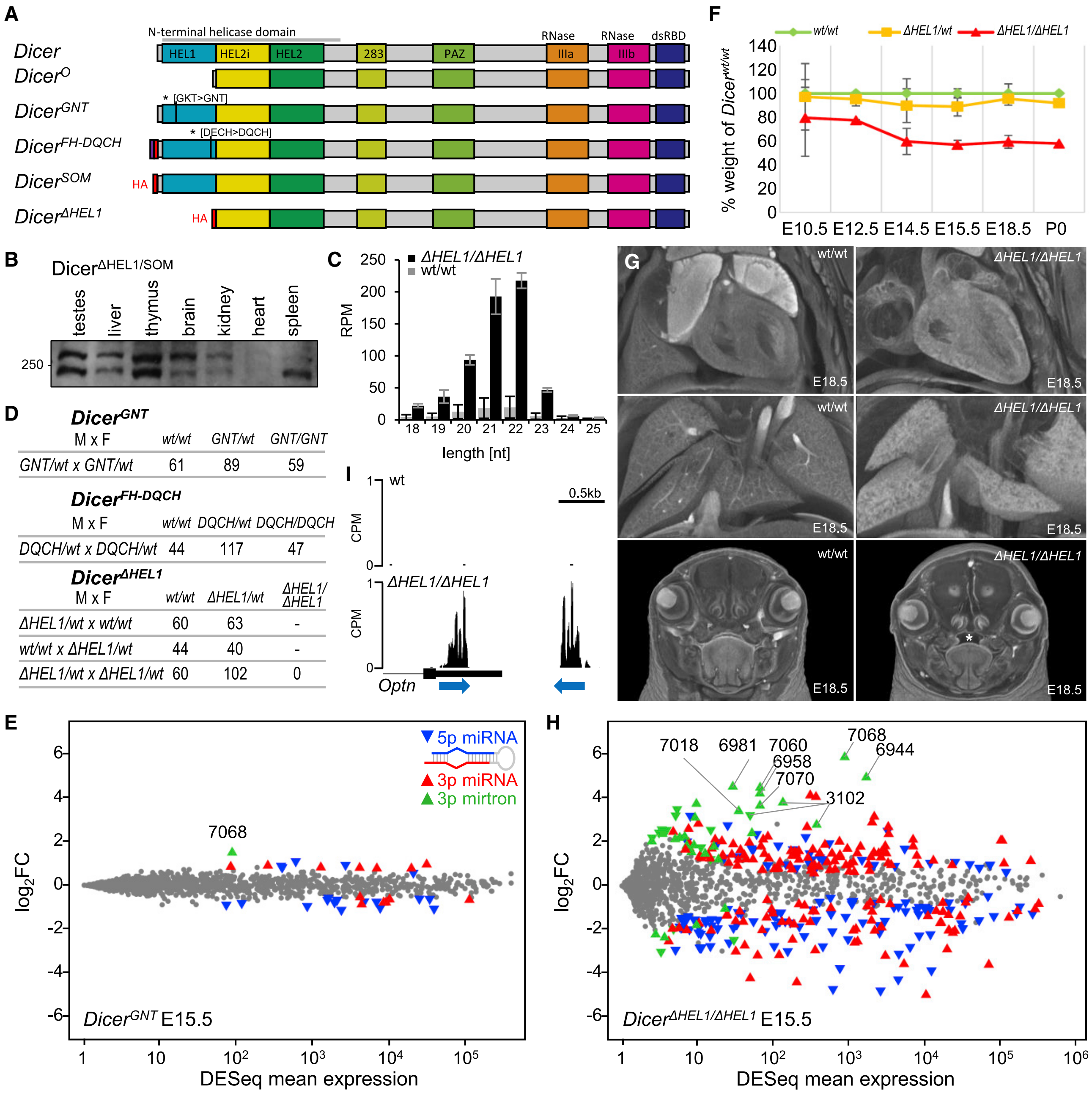

Figure 1. DExD/HEL1 domain of Dicer but not its ATPase activity is essential for miRNA homeostasis and normal mouse development

(A) Studied mouse Dicer protein variants and mutants. Dicer (full-length) and Dicer^{O} lacking HEL1 are endogenous isoforms. *Dicer*^{GNT} and *Dicer*$^{FH\text{-}DQCH}$ carry point mutations in HEL1 abolishing its function as ATPase. *Dicer*^{SOM} and *Dicer*$^{\Delta HEL1}$ are HA-tagged mutants with modified sequence encoding the N terminus. The engineered allele is designated *Dicer*$^{\Delta HEL1}$ to distinguish it from the endogenous *Dicer*^{O} isoform, which is transcribed from an oocyte-specific promoter.

(B) Western blot analysis of Dicer expression in different tissues of a heterozygote *Dicer*$^{\Delta HEL1/SOM}$ mouse.

(C) Production of endo-siRNAs from MosIR, a dsRNA-expressing plasmid (Flemr et al., 2013) in *Dicer*$^{\Delta HEL1/\Delta HEL1}$ ESCs. The y axis depicts reads per million (RPMs) per small RNA sequencing library. Data points, mean ± SD.

(D) Breeding performance of different heterozygous mutants.

(E) MA plot of small RNA-seq analysis of whole *Dicer*$^{GNT/GNT}$ E15.5 embryos compared with wild-type embryos (n = 3 for each genotype). Depicted are changes in levels of annotated murine miRNAs (miRBase 22.1; Kozomara et al., 2019). Significantly dysregulated 5p and 3p miRNAs (DESeq p value 0.05) are shown as oriented blue and red triangles, respectively. Mirtron non-canonical miRNAs are depicted by green triangles.

(F) Weight of embryos normalized to wild-type littermates shows relative retardation of *Dicer*$^{\Delta HEL1/\Delta HEL1}$ embryos—the growth retardation does not appear to be a simple proliferation defect (Figure S2B). Although a lower weight in *Dicer*$^{\Delta HEL1/\Delta HEL1}$ embryos is apparent at E10.5, the main reduction of growth appears between stages E12.5 and E14.5. Data points, mean ± SD.

(legend continued on next page)

clamp-like architecture of the RIG-I family of RNA helicases (Fairman-Williams et al., 2010), is located near RNase III domains and is composed of three globular subdomains: an N-terminal DExD/H subdomain (HEL1), which is separated by an insertion subdomain (HEL2i) from a helicase superfamily C-terminal subdomain (HEL2) (Figure 1A). The helicase also contacts the substrate (Lau et al., 2009, 2012; Taylor et al., 2013; Liu et al., 2018), but its exact function in the mammalian Dicer is enigmatic.

In animals and plants, the helicase domain and its ATPase activity appear linked to evolution of specialized Dicer variants and divergence of small RNA pathways. The miRNA-producing Dicer-1 in *Drosophila* does not hydrolyze ATP and its helicase domain is degenerated (Tsutsumi et al., 2011). In contrast, animal Dicers supporting RNAi, such as DCR-1 from *C. elegans* and DCR-2 from *Drosophila* have an intact helicase domain and hydrolyze ATP (Ketting et al., 2001; Liu et al., 2003; Cenik et al., 2011; Welker et al., 2011). ATP hydrolysis enables threading dsRNA substrates through Dicer's helicase (Sinha et al., 2018; Wei et al., 2021). In mammals, the highly conserved DExD/H domain in the miRNA-producing Dicer has invariantly preserved residues that would be necessary for ATPase activity (Jia et al., 2017; Cordin et al., 2006), but it does not exhibit the activity (Provost et al., 2002; Zhang et al., 2002) and inhibits RNAi instead (Ma et al., 2008; Kennedy et al., 2015). This paradox has not been resolved and the role of Dicer's helicase domain within mammalian small RNA pathways remains unclear.

Mice offer an outstanding model to study partitioning of miRNA and RNAi pathways as both pathways have essential roles relying on distinct Dicer isoforms expressed from a single gene. The miRNA pathway employs the full-length Dicer and is essential for gene control in embryo development and cell differentiation (reviewed in Park et al., 2010). RNAi is essential for oocytes and is supported by an oocyte-specific Dicer^{O} isoform, which lacks the DExD/H subdomain and generates miRNAs and siRNAs (Murchison et al., 2007; Tang et al., 2007; Flemr et al., 2013; Stein et al., 2015). Here, we provide evidence explaining how the DExD/H domain functions in an ATP-independent manner, segregates miRNA from the RNAi pathway *in vivo*, and makes Dicer an essential gatekeeper in miRNA biogenesis.

RESULTS

HEL1 structure, but not its ATPase activity, is necessary for viability and intact miRNome

To understand the importance of Dicer's helicase function *in vivo*, we produced mice carrying point mutations in the conserved HEL1 motifs, Walker A (^{69}GNT) and Walker B (^{175}DQCH), and mice lacking HEL1 entirely (*Dicer*$^{\Delta HEL1}$ mutant; Figures 1A and S1). The *Dicer*$^{\Delta HEL1}$ allele essentially encodes an HA-tagged Dicer^{O} protein. A control allele, designated *Dicer*^{SOM}, was produced previously (Taborska et al., 2019) to express HA-tagged full-length Dicer but lacking introns 2–6 like the *Dicer*$^{\Delta HEL1}$ allele (Figures 1A and S1H). The *Dicer*$^{\Delta HEL1}$ allele expressed the expected truncated Dicer variant (Figures 1B and S1K), and its functionality was confirmed in embryonic stem cells (ESCs), where it generated ~10× more siRNAs from long dsRNA than normal Dicer (Figure 1C).

The catalytically inactive *Dicer*$^{GNT/GNT}$ and *Dicer*$^{DQCH/DQCH}$ mutant mice were born in the expected Mendelian ratios (Figure 1D), appeared normal and were fertile. Small RNA analysis of *Dicer*$^{GNT/GNT}$ E15.5 embryos revealed minimal changes in the miRNome (Figure 1E). In contrast, mating of *Dicer*$^{\Delta HEL1/+}$ animals did not yield weaned *Dicer*$^{\Delta HEL1/\Delta HEL1}$ progeny (Figure 1D). *Dicer*$^{\Delta HEL1/\Delta HEL1}$ mutants showed embryonic growth retardation (Figure 1F) and died perinatally (Figure S2A), whereas *Dicer*$^{SOM/SOM}$ animals have normal viability (Taborska et al., 2019). Recovered *Dicer*$^{\Delta HEL1/\Delta HEL1}$ newborns were cyanotic, had breathing difficulties, and a body weight ~60% of heterozygous and wild-type siblings. *Dicer*$^{\Delta HEL1/\Delta HEL1}$ mice had anatomical aberrations including heart defects (Figure 1G) and underdeveloped lungs with reduced branching (Figures 1G and S2C). A contributing factor to the lethal phenotype could be reduced number of red blood cells and hemoglobin amount per red blood cell (Figure S2D).

Detrimental effects of Dicer$^{\Delta HEL1}$ protein could be either associated with toxicity of endogenous RNAi or with aberrant miRNA homeostasis. Small RNA analysis of *Dicer*$^{\Delta HEL1/\Delta HEL1}$ E15.5 embryos showed strong miRNome dysregulation (Figures 1H and S2E). At the same time, analysis of 21- to 23-nt-long RNAs in *Dicer*$^{\Delta HEL1/\Delta HEL1}$ E15.5 and ESCs did not find genomic loci giving rise to abundant pools of siRNAs from long dsRNA (Figure S2F). siRNAs from an inverted repeat in *Optn* 3′ region were increased but their abundance in *Dicer*$^{\Delta HEL1/\Delta HEL1}$ embryos was negligible (Figure 1I). Thus, miRNAs were the most affected abundant Dicer-derived small RNAs in *Dicer*$^{\Delta HEL1/\Delta HEL1}$ mutants.

At E15.5, homozygous loss of HEL1 altered the expression of ~1/4 embryonic miRNAs (386 of 1,199 miRNAs with abundance >1 read per million [RPM]; Figure 1H; Table S1) with approximately equal numbers of upregulated and downregulated miRNAs. Relative miRNA expression changes correlated well between *Dicer*$^{\Delta HEL1/\Delta HEL1}$ embryos and *Dicer*$^{\Delta HEL1/\Delta HEL1}$ ESCs (correlation coefficient 0.811, Figure S2G), suggesting that the miRNome remodeling considerably reflects direct effects of Dicer$^{\Delta HEL1}$ on miRNA biogenesis.

HEL1 inhibits mirtron biogenesis and regulates strand selection and miRNA sequence fidelity

Strikingly, a half of the 50 most upregulated miRNAs in *Dicer*$^{\Delta HEL1/\Delta HEL1}$ E15.5 embryos were mirtrons (Figure 1H;

(G) MicroCT scans of E18.5 embryos revealed in *Dicer*$^{\Delta HEL1/\Delta HEL1}$ embryos morphological aberrations in heart (right ventricle hypertrophy and loose hypomorphic myocardial walls in both atria), underdeveloped lungs, and cleft palate (asterisk, in 4/6 animals), which could also contribute to the perinatal lethality through maternal infanticide of non-feeding pups.

(H) MA plot of small RNA-seq analysis of whole *Dicer*$^{\Delta HEL1/\Delta HEL1}$ E15.5 embryos compared with wild-type embryos (three wild-type and five mutant embryos were sequenced). Data were plotted as in the (E).

(I) UCSC browser snapshot of E15.5 small RNAs from the *Optn* locus, which carries a transcribed inverted repeat (indicated by blue arrows) producing siRNAs in oocytes and embryonic stem cells (Flemr et al., 2013; Tam et al., 2008; Watanabe et al., 2008). CPMs, counts per million.

CellPress
OPEN ACCESS

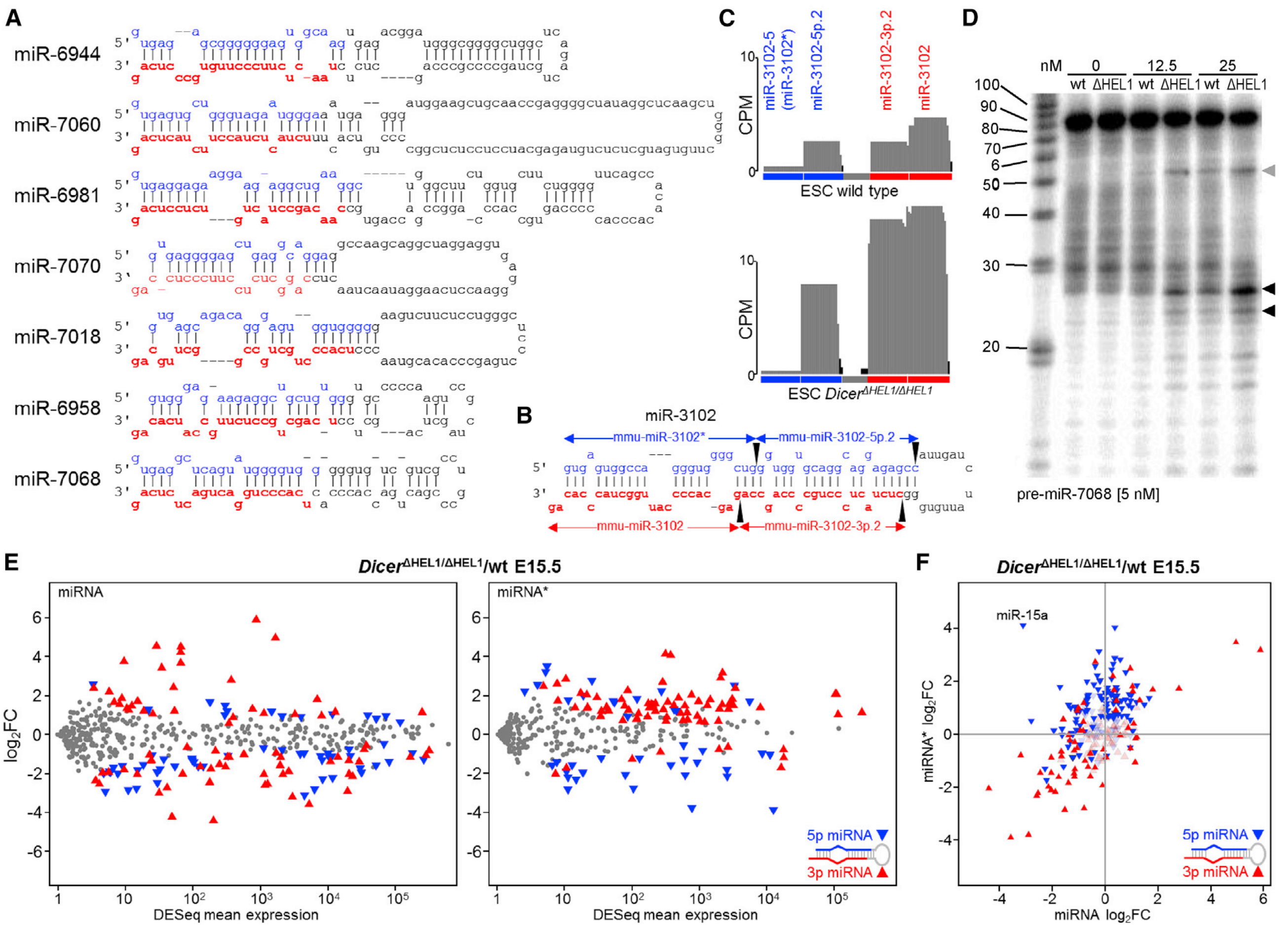

Figure 2. HEL1 restricts processing of mirtrons and 3p passenger strand loading

(A) Strongly upregulated mirtrons have extended stems and larger loops. Secondary structures were adopted from miRBase (Kozomara et al., 2019).

(B) miR-3102 is a unique mirtron cleaved by Dicer twice at points indicated by black arrowheads.

(C) Changes of miR-3102 levels in *Dicer*$^{\Delta HEL1/\Delta HEL1}$ ESCs. Relative expressions are shown in counts per million (CPMs) estimated by DESeq; 21–23 nt small RNA sequencing data were mapped on the genomic sequence (represented as the genomic locus in 5p–3p orientation), collapsed, and normalized per million of 21–23 nt reads. Gray columns represent sequences aligned with the annotated mature miRNA sequence; black represent sequences outside the annotated miRNA sequence.

(D) *In vitro* cleavage of miR-7068 precursor. Radiolabeled *in vitro* synthesized precursor (final conc. 5 nM) was incubated for 30 min with full-length Dicer (WT) or with Dicer$^{\Delta HEL1}$ (ΔHEL1). Reaction was resolved by PAGE and visualized by phosphorimaging. Black arrowheads depict two cleavage products corresponding to cleavage positions at the 3′ end of 5p miRNAs, a gray arrowhead depicts a product of an asymmetric cleavage. The experiment was repeated 3 times; a representative gel is shown. Apparent higher size of mir-7068 and cleavage products is caused by altered migration due to high G content.

(E) MA plots depicting relative changes of dominant miRNAs (left) and passenger strands (miRNA*, right) in *Dicer*$^{\Delta HEL1/\Delta HEL1}$ E15.5 embryos. 5p and 3p origins of significantly changed miRNAs or miRNA*s are distinguished by color and triangle orientation as depicted.

(F) Relative changes of dominant miRNAs and their passenger strands in *Dicer*$^{\Delta HEL1/\Delta HEL1}$ E15.5 embryos. Each triangle represents one miRNA:miRNA* pair, its color corresponds to the dominant strand (blue = 5p and red = 3p main strand). Triangle positions indicate relative changes of the dominant miRNA (x axis) and its miRNA* (y axis). Deep colors indicate significantly dysregulated miRNAs.

Table S1), non-canonical miRNAs whose precursors are spliced out specific small introns (Berezikov et al., 2007; Ladewig et al., 2012). Upregulated mirtron precursors featured relatively long stems and/or loops (Figures 2A–2C), miR-3102 comprising such a long stem that it carries two consecutive miRNAs (Chiang et al., 2010). The increase in mirtron expression was not transcriptional as mirtron-encoding host genes were not upregulated in *Dicer*$^{\Delta HEL1/\Delta HEL1}$ ESCs (Table S2). Consistent with RNA sequencing (RNA-seq) data, Dicer$^{\Delta HEL1}$ cleaved the 5′ radiolabeled pre-miR-7068 (the most upregulated mirtron) *in vitro* more efficiently than normal Dicer (Figure 2D). Notably, both Dicers cleaved pre-miR-7068 *in vitro* also in non-canonical ways, producing a fragment corresponding to a partial precursor cleavage at the 5′ end of a 3p miRNA (Figure 2D). Taken together, Dicer$^{\Delta HEL1}$ is more tolerant of extended pre-miRNA stems and loops of mirtrons than the full-length enzyme, suggesting that HEL1 physiologically restricts biogenesis of small RNA from such substrates.

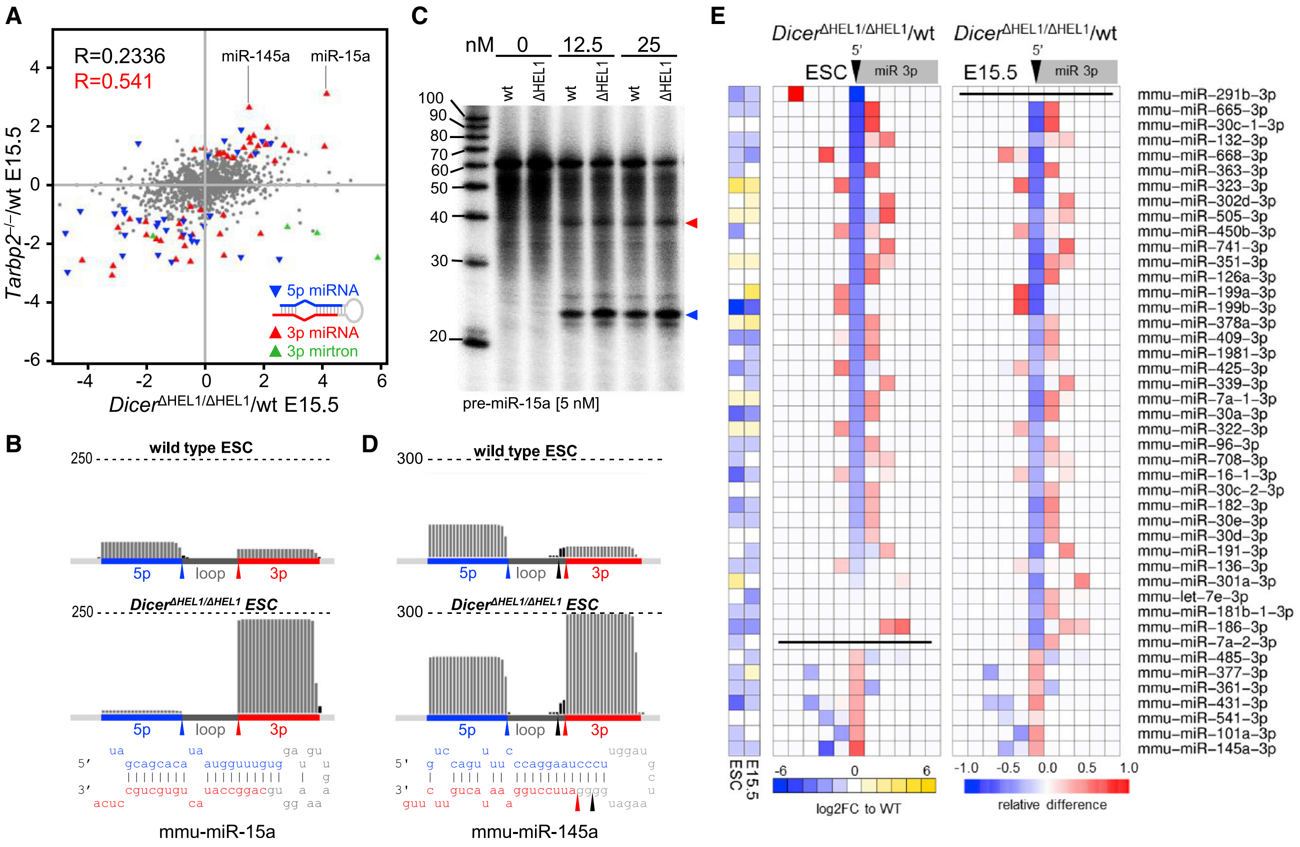

Figure 3. HEL1 is important for pre-miRNA cleavage fidelity

(A) Comparison of relative changes of miRNAs in *Tarbp2*$^{-/-}$ and *Dicer*$^{\Delta HEL1/\Delta HEL1}$ E15.5 embryos. Highlighted are miRNAs significantly differentially expressed in *Tarbp2*$^{-/-}$. Most miRNAs differentially expressed in *Tarbp2*$^{-/-}$ E15.5 (Pullagura et al., 2018) showed changes in the same direction in *Dicer*$^{\Delta HEL1/\Delta HEL1}$ E15.5 embryos.

(B) miR-15a exhibits a strong bias toward 3p strand selection in *Dicer*$^{\Delta HEL1/\Delta HEL1}$ ESCs. Relative miRNA expression is shown in counts per million (CPMs) estimated by DESeq. The graph construction was the same as in Figure 2C.

(C) miR-15a *in vitro* cleavage assay. 5 nM *in vitro* synthesized P^{32} 5′ end labeled pre-miRNA was incubated with indicated concentrations of recombinant Dicer variants at 37°C for 60 min, resolved by PAGE, and visualized by phosphorimaging. Blue and red arrowheads point to products corresponding to cleavage sites giving rise to 5p and 3p miRNA, respectively.

(D) miR-145a exhibits a strong bias toward 3p strand selection in *Dicer*$^{\Delta HEL1/\Delta HEL1}$ ESCs and variability of the 5′ end of the 3p miRNA. The graph construction was the same as in Figure 3B.

(E) The heatmap depicts analysis of the cleavage site at the 5′ end of 3p miRNAs in 50 most affected 3p miRNAs in E15.5 embryos and ESCs. The cleavage site is indicated by a black arrowhead. Each column of squares represents one nucleotide from the cleavage site in direction into the mature 3p miRNA (to right) or upstream of it (to left). Red-blue colors indicate relative changes in the 3p miRNA cleavage site when compared with the wild-type sample.

Another impact of Dicer$^{\Delta HEL1}$ on miRNome concerned passenger strands (miRNA*), the miRNA strands less likely to be loaded onto AGO effector protein. There was a striking preferential upregulation of miRNA* from the downstream strand of the stem loop precursor (denoted 3p) and downregulation of miRNA* from the upstream strand (5p) in both, *Dicer*$^{\Delta HEL1/\Delta HEL1}$ E15.5 embryos and *Dicer*$^{\Delta HEL1/\Delta HEL1}$ ESCs (Figures 2E and S2H). A slight opposing effect was observed for many leading (much more abundant) miRNA counterparts, but in some cases, the opposing effect was stronger, and exceptionally strong in case of miR-15a (Figures 2F and S2I). Since passenger strands typically have much lower abundance than main strands, their high relative increase would be expected to cause a minor, if experimentally detectable, reduction of corresponding 5p leading miRNAs. To sum up, the loss of HEL1 affects the thermodynamic sensing of the 5′ end of 3p miRNA and facilitates its selection for AGO loading.

Strand selection has been associated with Dicer's binding partner TARBP2 (Noland et al., 2011). Since TARBP2 binds the HEL2i subdomain adjacent to HEL1 (Liu et al., 2018; Wilson et al., 2015), we examined whether the loss of HEL1 impairs binding of TARBP2 to Dicer$^{\Delta HEL1}$. Co-immunoprecipitation of TARBP2 with Dicer showed that TARBP2 remains associated with Dicer$^{\Delta HEL1}$ (Figure S3A), suggesting that miRNome remodeling in *Dicer*$^{\Delta HEL1/\Delta HEL1}$ E15.5 embryos is not caused by the loss of interaction between Dicer and TARBP2. Importantly, analysis of miRNome in *Tarbp2*$^{-/-}$ E15.5 embryos (Pullagura et al., 2018) identified 84 differentially regulated miRNAs (>1 RPM, DESeq p value < 0.05, Table S1), majority of which followed a similar trend also in *Dicer*$^{\Delta HEL1/\Delta HEL1}$ E15.5 embryos (Figure 3A). Therefore,

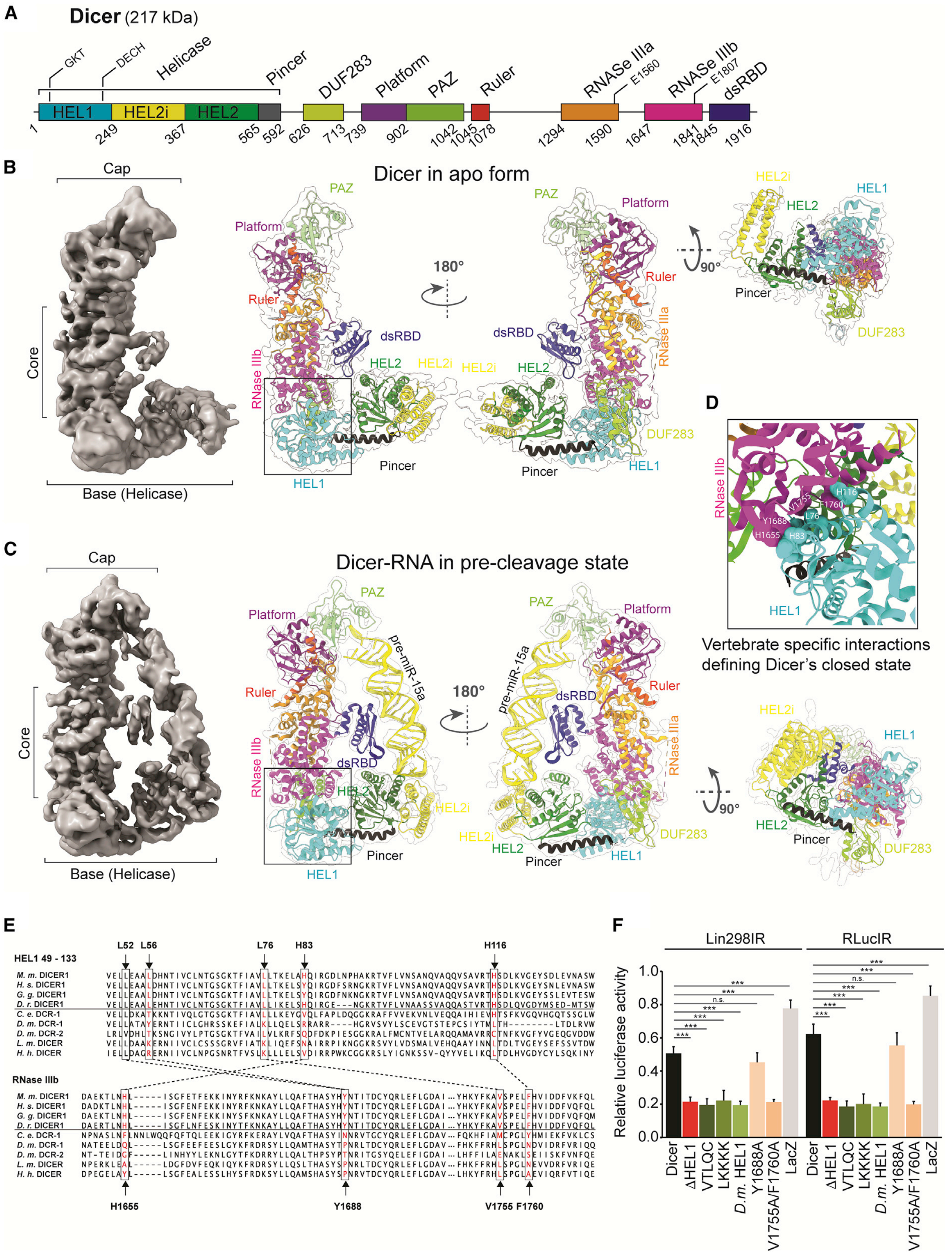

(legend on next page)

we hypothesize that HEL1 and TARBP2 exert similar but non-redundant thermodynamic sensing, which controls selection of the 5′ end of a 3p miRNA.

RNA-seq data revealed two features of miRNA biogenesis present in subsets of differentially expressed miRNAs:partial precursor cleavage and fidelity of mature miRNA biogenesis. These features can be demonstrated on miR-15a and miR-145a, two miRNAs exhibiting increased 3p miRNA* levels in *Dicer*$^{ΔHEL1/ΔHEL1}$ and *Tarbp2*$^{-/-}$ E15.5 embryos (Figure 3A) and displaying a strong opposing effect on 5p miRNA and its 3p miRNA* levels (Figures 3B and S3B).

In case of miR-15a, RNA-seq data revealed an asymmetric pre-miR-15a cleavage, where Dicer would cleave at the 5′ end of a 3p miRNA, whereas the concurrent cleavage at the 3′ end of a 5p miRNA would not occur. A partially cleaved pre-miR-15a fragment is produced by the full-length Dicer, whereas the relative amount of the pre-miR-15a fragment is higher in *Dicer*$^{ΔHEL1/ΔHEL1}$ samples (Figure S3C). In depth analysis of RNA-seq data identified tens of miRNAs having a miR-15a-like frequency of fragments cleaved just at the 5′ end of a 3p miRNA (Table S3). The partial cleavage by Dicer is also observed for pre-miR-15a (Figure 3C) but not for miR-145a *in vitro* (Figure S3D). Since the partial cleavage was also made by the full-length Dicer (Figures 3C and S3C), it appears to be a miRNA-specific feature pronounced by Dicer^{ΔHEL1} because of its higher activity and altered thermodynamic sensing. Whether the intrinsic partial cleavage by Dicer^{ΔHEL1} facilitates 3p miRNA* strand selection similarly to defective RNase IIIb mutations (Anglesio et al., 2013) requires further investigation.

In case of miR-145a, the strand switch correlated with an apparent cleavage position shift at the 5′ end of miR-145a-3p resulting in high abundance of a two-nucleotide shorter miR-145a-3p isomiR in *Dicer*$^{ΔHEL1/ΔHEL1}$ mutants (Figure 3D). The loss of two G:C base pairs and presence of a 5′ A nucleotide should favor the shorter miR-145-3p strand selection (Medley et al., 2021). This observation prompted a systematic analysis of the 5′-terminal nucleotide fidelity because the cleavage position defining 5′-terminal nucleotides in 3p miRNAs affects nucleotides 2–7, known as the "seed sequence" guiding target recognition and binding (Brennecke et al., 2005; Lewis et al., 2003). A change in the seed sequence would be biologically significant even if miRNA abundance would not change (Mencía et al., 2009). Shifts in the 5′ end of 3p miRNAs had a similar pattern in *Dicer*$^{ΔHEL1/ΔHEL1}$ E15.5 embryos and ESCs (Figures 3E and S3E), suggesting that most of them are a direct consequence of the loss of HEL1. However, RNA-seq data do not allow to distinguish a truly altered cleavage point of Dicer^{ΔHEL1} from altered strand selection among isomiRs. In any case, a 5′ end terminal nucleotide shift was found in at least 20% of abundant 3p miRNAs in *Dicer*$^{ΔHEL1/ΔHEL1}$ E15.5 embryos and ESCs. In contrast, terminal nucleotide fidelity in *Dicer*$^{GNT/GNT}$ E15.5. mutant was essentially unaffected (Figure S3E). Notably, terminal nucleotide fidelity was found to be also affected in *Tarbp2*$^{-/-}$ embryos (Pullagura et al., 2018), and approximately, a half of the cleavage alterations in *Dicer*$^{ΔHEL1/ΔHEL1}$ samples were observed in *Tarbp2*$^{-/-}$ embryos (Figure S3E). It is likely that the loss of TARBP2 affects miRNA biogenesis through thermodynamic sensing/strand selection of variably cleaved pre-miRNAs, but we cannot exclude that absence of TARBP2 also affects cleavage fidelity.

HEL1-RNase IIIb interaction stabilizes Dicer's closed conformation and shapes substrate selection

To obtain further insights into the role of HEL1, we determined the 3.8-Å-resolution cryo-EM structure of mouse full-length Dicer in the apo form and the 4.2-Å-resolution structure of the complex of the full-length mouse Dicer with a 59-nt pre-miR-15a (Figures 4A–4C, S4, and S5; Table S4); this miRNA was selected for its unique behavior in *Dicer*$^{ΔHEL1/ΔHEL1}$ mutants (Figures 2 and 3B).

Akin to human Dicer, the overall structure of mouse Dicer shows an identical "L shape" architecture (Liu et al., 2018), adopting a "closed" state (Figure 4B). Cryo-EM data also suggest that the helicase domain is flexible around HEL1 to some extent, which is consistent with previous observations (Taylor et al., 2013; Liu et al., 2018; Figure S4). The highest resolution of the full-length metazoan Dicer determined so far allowed us to dissect the molecular details of the closed state. We identified the residues at the interface between DExD/H and RNase IIIb that lock the closed state of Dicer (Figure 4D). Interestingly, these aliphatic and aromatic amino acids residues are conserved across vertebrates but not in invertebrates (Figure 4E).

Similarly as reported for human Dicer-RNA structure (Liu et al., 2018), the full-length Dicer●pre-miR-15a structure captured

Figure 4. Cryo-EM structures of mouse full-length Dicer alone and in complex with Dicer●pre-miRNA reveal the molecular basis of locking Dicer in the closed state

(A) Domain architecture of full-length mouse Dicer numbered at boundaries.

(B) Overall structure of the full-length mouse Dicer, shown as 3.8-Å cryo-EM density map and ribbon representations in two orthogonal views. Interface between the HEL1 and RNase IIIb domains is highlighted by a box.

(C) Overall structure of the full-length mouse Dicer-RNA complex, shown as 4.2-Å cryo-EM density map and ribbon representations in two orthogonal views.

(D) A close-up of HEL1-RNase IIIb domain interface with interacting amino acid residues.

(E) Multiple sequence alignments of HEL1 and RNase IIIb. Conserved residues in vertebrates depicted in red and their contacts in dotted lines. *M.m.*, *Mus musculus*; *H.s.*, *Homo sapiens*; *G.g. Gallus*; *D.r., Danio rerio*; *C.g.*, *Crassostrea gigas* (oyster); *C.e.*, *Caenorhabditis elegans*; *D.m.*, *Drosophila melanogaster*; and *T.c., Tribolium castaneum*.

(F) RNAi assay performed in *Pkr*$^{-/-}$ NIH 3T3 cells expressing mouse Dicer variants. Mutated Dicer variants included ΔHEL1 variant, mutations of the five amino acids in HEL1 (labeled in the E) to amino acids aligning with *Drosophila* DCR-2 (VTLQC) or amino acids antagonizing the interaction (LKKKK), and substitution of the murine HEL1 with the HEL1 from *Drosophila* DCR-2 (*D.m.* HEL1). We also substituted RNase IIIb residues predicted to interact with HEL1: Y1688A and V1755A/F1760A (labeled in the E). RNAi was induced with two different long dsRNA hairpins targeting *Renilla* luciferase reporter carrying sequences complementary to these dsRNAs (Demeter et al., 2019). LacZ sample level corresponds to endogenous RNAi activity observed in *Pkr*$^{-/-}$ 3T3 cells. Data points, mean ± SD. ***$p < 0.001$; n.s. (non-significant).

CellPress
OPEN ACCESS

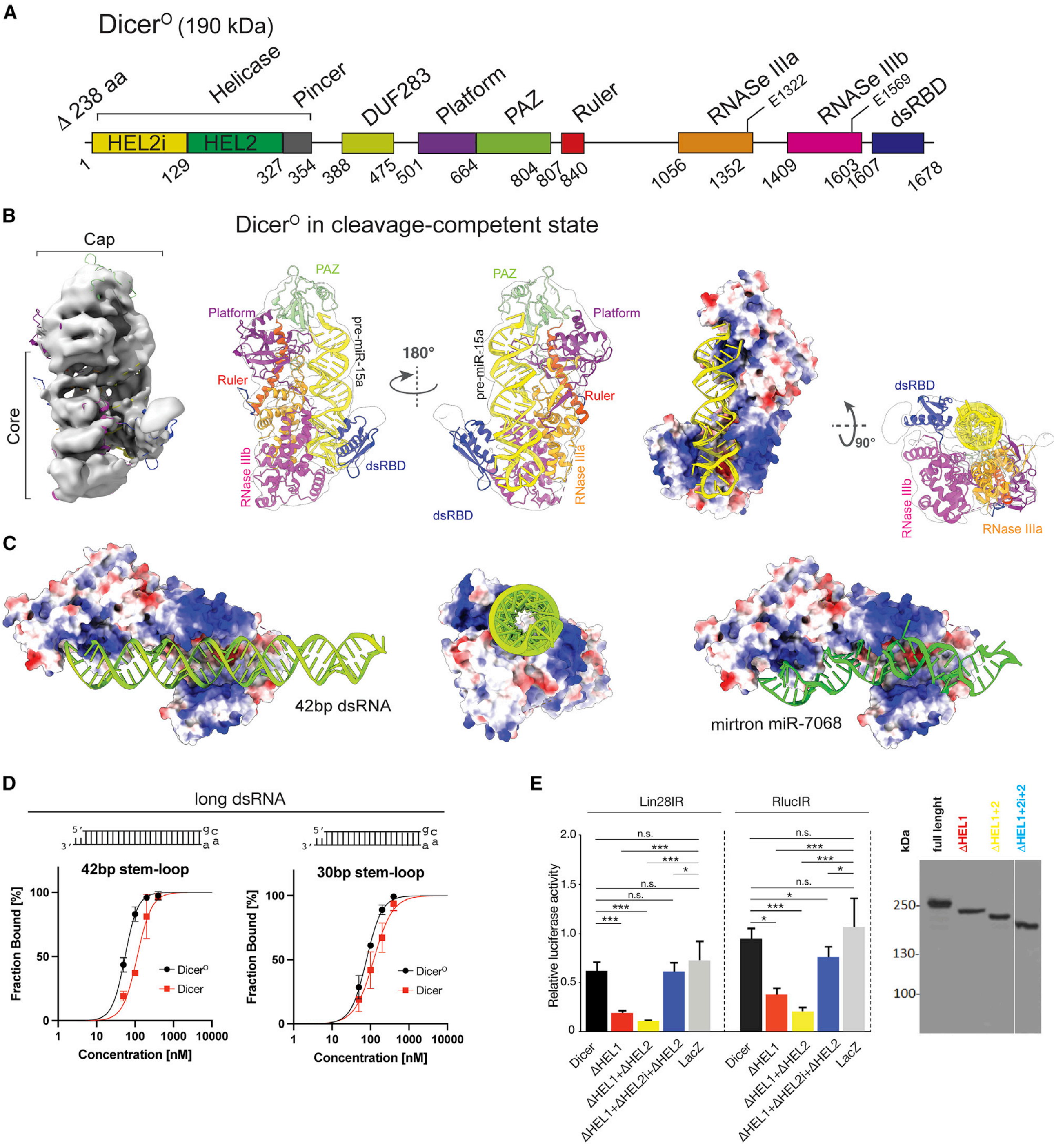

Figure 5. Cryo-EM structure of mouse Dicer^{O}●RNA complex reveals why the absence of HEL1 makes Dicer active and promiscuous

(A) Domain architecture of Dicer^{O} numbered at boundaries.

(B) Overall structure of the Dicer^{O}●RNA complex in cleavage-competent state shown as 6.2-Å cryo-EM density map, ribbon representations in orthogonal views, and electrostatic surface view.

(C) Rigid-body docking of long dsRNA (left, side view; middle, top view) and mirtron miR-7068 (right) into the Dicer^{O} structure in cleavage-competent state shown as electrostatic surface view.

(D) Quantification of electrophoretic mobility shift assays of Dicer isoforms with long dsRNA hairpins carrying a GCAA-tetraloop. $K_D^{42bp\ stem\text{-}loop}$ = 55 ± 4 and 115 ± 20 nM for Dicer^{O} and Dicer, respectively; $K_D^{30bp\ stem\ loop}$ = 79 ± 7 and 120 ± 27 nM for Dicer^{O} and Dicer, respectively. Data points, mean ± SD (n = 2–3).

(legend continued on next page)

Dicer only in the pre-cleavage state in our cryo-EM data (Figures 4C and S5A–S5H). In the pre-cleavage state, the PAZ domain anchors the 3′ end of the pre-miR-15a, but not the 5′ end, in contrast to the human enzyme binding pre-let-7 (Liu et al., 2018; Tian et al., 2014), likely reflecting the absence of the human-specific α helix in the PAZ domain (Figure S5I). The β sheet face of the dsRNA-binding domain (dsRBD) binds the central double-helical region of the pre-miR-15a (Figures 4C and S5J), in contrast to other members of the dsRBD family, which typically interact with RNA via their α-helical face (Stefl et al., 2005). The terminal loop of pre-miR-15a binds to the outer rim of the helicase subdomains HEL2i and HEL2 (Figure 4C). Overall, these interactions are characteristic of the Dicer closed state and position pre-miRNA away from the RNase III catalytic sites. The closed state may allow Dicer to recognize specific structural features of miRNA precursors, whereas it would impair processing of mirtrons and dsRNAs because they cannot be optimally recognized due to steric hindrance in the pre-cleavage state (Figure S5K).

Importantly, the RNA-bound full-length Dicer closed state is virtually identical to that of the apo form and is stabilized by the same residues at the DExD/H and RNase IIIb interface (Figure 4D). We thus examined their functional significance in Dicer variants where the five key residues (LLLHH, Figure 4E) in HEL1 were changed to LKKKK or to VTLQC (residues in aligned *Drosophila* DCR-2 sequence). In addition, we replaced the entire HEL1 with the HEL1 from *Drosophila* DCR-2 (*D.m*. HEL1 variant). On the RNase IIIb side, we substituted residues Y1688 and V1775 together with F1760 to alanines. All variants were expressed in $Pkr^{-/-}$ NIH 3T3 cells, and their effect on RNAi was tested using long dsRNA expression targeting a luciferase reporter described previously (Demeter et al., 2019). The LKKKK, VTLQC, and *D.m*. HEL1 variants as well as the V1755A/F1650A RNase IIIb variant stimulated RNAi indistinguishably from ΔHEL1 (Figure 4F), suggesting that these substitutions unlock the closed state equally well as the loss of the entire HEL1 subdomain. Notably, the V1755A/F1650A variant highlights the significance of the interface between DExD/H and RNase IIIb because it has high RNAi activity in the presence of the intact HEL1 domain. These data imply that the equilibrium between the closed and open states is sensitive to structural alterations at the interface between DExD/H and RNase.

Dicer^{O} is structurally primed to form a cleavage-competent substrate binding

To understand the structural mechanism by which Dicer^{O} can support both RNAi and miRNA pathways, we used cryo-EM to analyze this murine Dicer isoform in its apo form and in complex with a miRNA precursor. Although the structure of Dicer^{O} in the apo form could not be determined due to its inherent flexibility, we were able to determine the 6.2-Å-resolution cryo-EM structure of Dicer^{O} in complex with pre-miR-15a (Figures 5A, 5B, and S6A–S6G; Table S4). Importantly, our Dicer^{O}-RNA structure captured Dicer^{O} exclusively in a cleavage-competent state (Figure S6). The overall structure of the Dicer^{O}●pre-miR-15a complex shows that the helicase and DUF283 domains had faint densities in the cryo-EM data and could not be built into the model (Figure S6). A control experiment showed that Dicer^{O} on the grid was intact suggesting that the weak and missing protein density is due to their inherent flexibility in the cleavage state (Figure S6). This is in contrast to the structural observations for DCR-1 from *Drosophila* (Jourevleva et al., 2022) where the helicase and DUF283 domains do not exhibit such flexibility.

In the cleavage-competent state, the PAZ-Platform cassette anchors the 3′ and 5′ ends of the pre-miR-15a and the RNA is accommodated in the positively charged groove formed by the RNase IIIa/b domains (Figure 5B). The dsRBD of Dicer interacts with the RNA using its α-helical face and contacts the minor and major grooves of the pre-miRNA, whereas the β1-β2 loop binds to the terminal loop of the pre-miRNA (Figures 5B and S6I). The dsRBD clamps the RNA in the catalytic sites of Dicer and the alignment of the RNA with the catalytic sites suggests that mouse Dicer^{O} cleaves pre-miR-15a between bases G22 and G23 and between bases G37 and C38, producing a 22-nt miRNA duplex (Figure S6J). This matches pre-miR-15a cleavage sites annotated in the miRbase (Kozomara et al., 2019). Interestingly, the terminal loop of pre-miR-15a interacts with the dsRBD and the RNase IIIb domains but exhibits imperfect alignment with the RNase IIIb catalytic site, which is consistent with the asymmetric cleavage of pre-miR-15a at the 5′ end of 3p miRNA described above (Figures 3C and S3C).

Modeling of different substrates in the cleavage state of Dicer^{O} revealed accommodation of miR-7068 and long dsRNA without steric hindrance (Figure 5D). This is consistent with higher affinity of Dicer^{O} for longer perfectly complementary dsRNAs (Figure 5D), higher affinity of full-length Dicer to pre-miRNAs compared with longer perfectly complementary dsRNAs (Figure S6K), and stimulation of RNAi in human cells by expressing the human equivalent of Dicer^{O} (Figure 5E). We conclude that Dicer^{O} without HEL1 exists in an open state that allows direct loading of precursors for both RNAi and miRNA pathways.

Dicer●pre-miR-15a●TARBP2 complex structure shows that TARBP2 promotes transition to the cleavage-competent state

It has been reported that Dicer's accessory proteins, such as TARBP2 and ADAR1, associate with the DExD/H helicase domain and stimulate cleavage of pre-miRNAs by Dicer (Liu et al., 2018; Wilson et al., 2015; Chendrimada et al., 2005; Ota et al., 2013). We hypothesized that TARBP2 may stimulate the transition from the pre-cleavage to cleavage state and increase the probability to capture the latter state by cryo-EM. To this end, we reconstituted the ternary complex between Dicer, pre-miR-15a, and TARBP2 by direct mixing and omitting size

(E) Relative RNAi-mediated repression in $Pkr^{-/-}$ U-2 OS cells expressing different N-terminally truncated human Dicer variants and the two different long dsRNA hairpins targeting *Renilla* luciferase reporter carrying sequences complementary to these dsRNAs (Demeter et al., 2019). LacZ sample level corresponds to endogenous RNAi activity observed in $Pkr^{-/-}$ U-2-OS cells. Comparable expression of truncated Dicer proteins was confirmed by western blotting shown right. Loss of HEL1 or HEL1+HEL2 (but retaining HEL2i) yields similar robust RNAi effect with both dsRNAs. Data points, mean ± SD (n = 4–5; in triplicate transfections). *p < 0.05; **p < 0.01; ***p < 0.001; p > 0.05, n.s. (non-significant).

CellPress
OPEN ACCESS

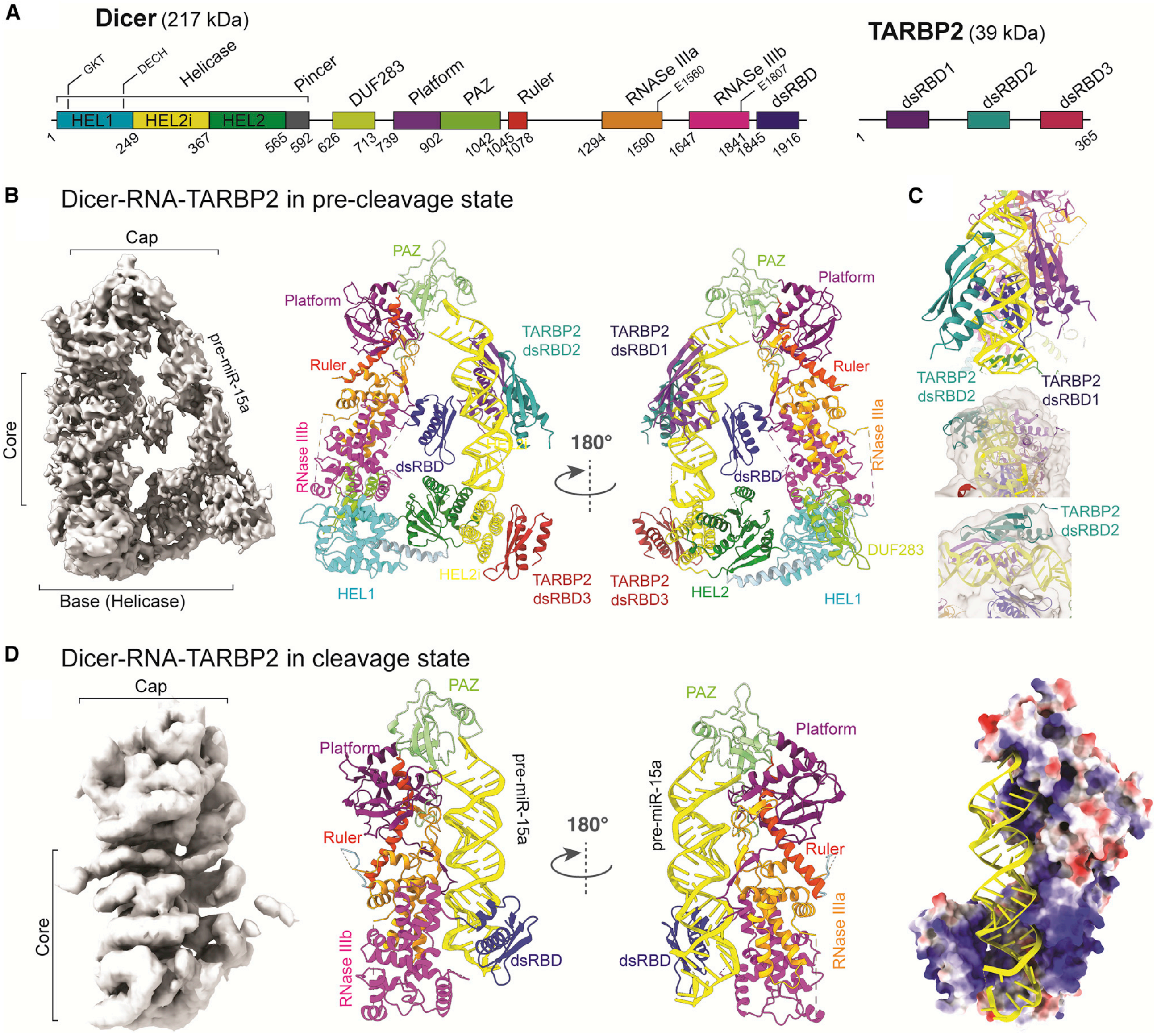

Figure 6. Cryo-EM structures of mouse Dicer●RNA●TARBP2 complexes reveal that TARBP2 allosterically stimulates the transition from pre-cleavage to cleavage state

(A) Domain architecture of Dicer (left) and TARBP2 (right) numbered at boundaries.

(B) Overall structure of the Dicer●pre-miR-15a●TARBP2 complex in the pre-cleavage state shown as 3.81-Å cryo-EM density map and ribbon representations.

(C) A close-up of TARBP2 dsRBD12-RNA interface showing mutual asymmetrical binding of dsRBDs (top). Cryo-EM density maps and the superimposed models of TARBP2 dsRBD12 are shown as transparent surfaces and ribbons, respectively (middle, bottom).

(D) Overall structure of the Dicer●pre-miR-15a●TARBP2 complex in the cleavage-competent state shown as 5.91-Å cryo-EM density map, ribbon representations, and electrostatic surface view.

exclusion chromatography to preclude selection of a given state and to preserve the conformational diversity of the sample. Cryo-EM analysis of the ternary complex revealed the co-existence of two states that resembled the pre-cleavage and cleavage states based on the 2D class averages and 3D classification (Figures S7A–S7K).

The pre-cleavage state dominates the single-particle population, and we determined its 3.81-Å-resolution cryo-EM structure (Figures 6A, 6B, and S7A–S7G). The Dicer●pre-miR-15a●TABRP2 structure in the pre-cleavage state showed an identical closed structure as that of the Dicer●pre-miR-15a complex (Figure 4C). As for TARBP2 in the ternary complex, our cryo-EM reconstruction clearly resolves the interaction between the dsRBD3 of TARBP2 with the HEL2i helicase subdomain (Figure 6B). The densities of the dsRBD1 and dsRBD2 of TARBP2 are weaker, which suggests these two dsRBDs do not bind pre-miR-15a specifically but rather bind in multiple registers, with a slight preference for specific shape in the central regions

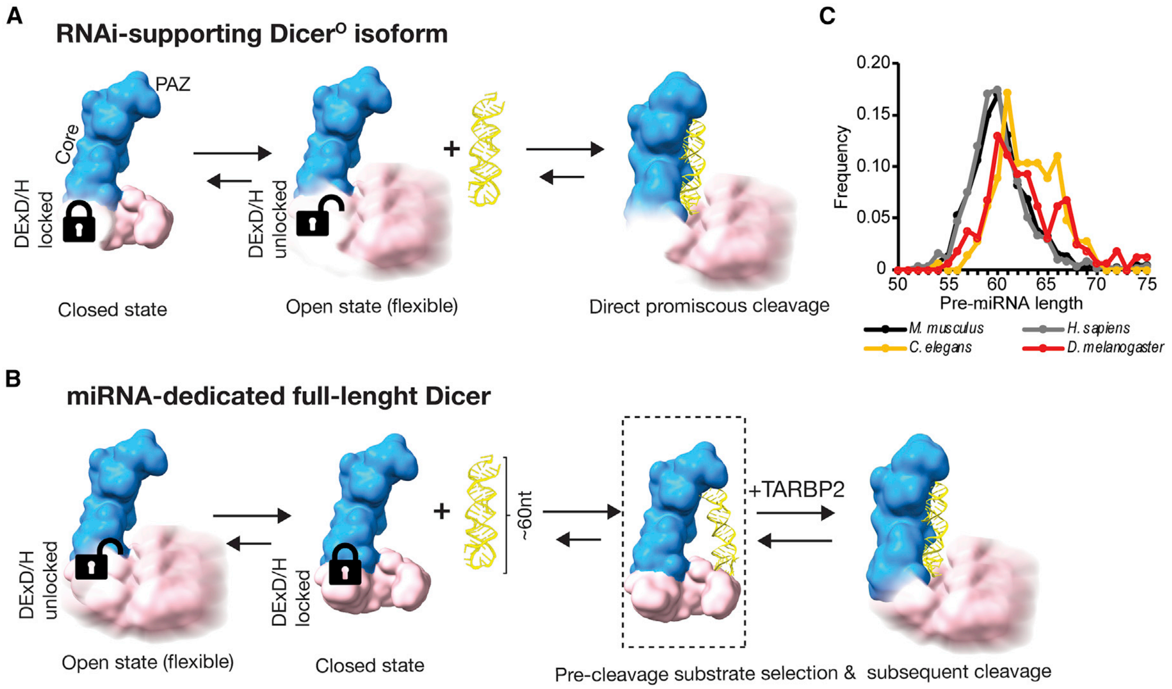

Figure 7. Model of Dicer function and miRNA and RNAi pathway partitioning in mammals

(A) Dicer exhibits conformational dynamics of its helicase domain, which exists in two conformations: a major conformation, the closed state, and a minor conformation, the open state. The absence of HEL1 in the RNAi-supporting Dicer^{O} isoform shifts the equilibrium to favor the open state, allowing direct loading of RNA substrates to form a cleavage-competent state.

(B) miRNA-dedicated full-length Dicer favors the closed state that is locked by the interaction between the DExD/H and RNase IIIb domains. The closed state is used to form a pre-cleavage complex with the miRNA precursor substrates. Subsequently, Dicer switches into the open state (allosterically activated by TARBP2) and the pre-bound substrate is transferred to the catalytic site of Dicer for cleavage. Models are based on experimental data and are shown in lower resolution for clarity. dsRBD is omitted from the models for clarity.

(C) Distribution of pre-miRNA lengths in four species based on high-confidence miRNA annotation in MirGeneDB (Fromm et al., 2022).

where we observed weak densities (Stefl et al., 2010; Wang et al., 2011). Nonetheless, we could unambiguously fit the dsRBDs to these densities (Figure 6C). TARBP2 dsRBD1 and 2 (dsRBD12) bind pre-miR-15a in mutual asymmetric arrangement in the context of the Dicer●pre-miR-15a●TABRP2 ternary complex, in contrast to isolated siRNA, which has been shown to be recognized symmetrically by TARBP2 dsRDB12. In the pre-cleavage state, the dsRBD of Dicer binds RNA using its β-sheet face which is a non-canonical arrangement (Stefl et al., 2005).

A minor population of about 15% of particles in our cryo-EM data resembled the cleavage state (Figure S7) that we observed for Dicer^{O} (Figures 5 and S6). Using these particles, we determined the 5.91-Å-resolution cryo-EM structure of the Dicer●pre-miR-15a●TABRP2 complex (Figures 6D and S7H–S7K). Overall, the structure of the Dicer●pre-miR-15a●TABRP2 complex in the cleavage state (Figure 6D) is strikingly similar to the structure of Dicer^{O}●pre-miR-15a complex (Figure 5B); the dsRBD of Dicer interacts via its α-helical face and clamps pre-miRNA in the positively charged groove formed by the RNase IIIa/b domains (Figure 6D). Notably, the structure of this ternary complex lacks not only the helicase and DUF283 domains but no density was also found for TARBP2. Furthermore, the binding register of TARBP2 dsRBD2 of the pre-cleavage state is incompatible with the RNA-binding register of Dicer dsRBD (Figure S7L). This implies that TARBP2 binding may promote unlocking the closed state of Dicer, an effect similar to the removal of HEL1. Subsequent accommodation of pre-miRNA in the dicing state may dismantle TARBP2-RNA interactions. At the same time, TARBP2 could hold the pre-miRNA in place when Dicer oscillates between its common closed and rare open states. We conclude that mammalian Dicer likely functions by a two-step mechanism (Figure 7): (1) binding of enzyme to substrate forms an inactive closed complex that facilitates substrate selection and (2) upon binding of the substrate and TARBP2, Dicer switches into an active open state that allows repositioning of the substrate into the catalytic site of Dicer.

DISCUSSION

Understanding the molecular principles governing co-existence and partitioning of miRNA and RNAi pathways is important since both pathways are of great biological, medical, and biotechnological importance. Different mechanistic and functional partitioning of miRNA and RNAi pathways exists in *Metazoa*: *C. elegans* utilizes a single Dicer for both pathways, whereas two dedicated Dicer paralogs evolved in *Drosophila*, the ATP-dependent DCR-2 for RNAi, and the miRNA pathway-supporting Dicer-1 with degenerated DExD/H helicase domain. The

mammalian Dicer supports the miRNA pathway, although it is ATP-independent (Provost et al., 2002; Zhang et al., 2002) and does not efficiently process dsRNA into siRNA *in vivo* (Demeter et al., 2019; Nejepinska et al., 2012). Our results reveal how mammalian Dicer is specifically adapted to produce small RNAs, how it is committed to the miRNA pathway, and how it suppresses endogenous RNAi.

We propose a model (Figure 7) where the DExD/H (HEL1) evolved into an ATP-independent critical structural element of mammalian Dicer's architecture. An interaction between the DExD/H and RNase IIIb domains locks the rest of the helicase domain (HEL2 and HEL2i) in a stable closed state in which Dicer recognizes miRNA precursors by anchoring three elements: the RNA ends, the central region, and the terminal loop. Substrate loading into this pre-cleavage state was observed also for the human Dicer bound to let-7a (Liu et al., 2018). The pre-cleavage state may serve as a kinetic trap for diffusion-driven screening of optimal substrates and suppress biogenesis of small RNAs from substrates such as long dsRNA or mirtrons, which deviate from conventional miRNA precursors. This cleavage-incompetent arrangement of the Dicer-substrate complex appears a specific feature of mammalian miRNA biogenesis because such a structural arrangement is not observed in *Drosophila* or in plant Dicer-substrate structures (Liu et al., 2018; Wei et al., 2021; Jourevleva et al., 2022; Wang et al., 2021). Notably, high confidence mammalian miRNAs (Fromm et al., 2022) have a pre-miRNA length distribution, which is distinct from *Drosophila* or *C. elegans* (Figure 7C). This may reflect an impact of Dicer's architecture on evolution of its substrates where the highly conserved Dicer's rigid architecture would offer a stable structural "mold" for adaptive evolution of vertebrate miRNAs precursors. This could be a significant factor behind extraordinary expansion of vertebrate miRNAs (Campo-Paysaa et al., 2011), which stochastically evolved into Dicer substrates from random RNA structures (Meunier et al., 2013).

Analysis of miRNAs in *Dicer*$^{\Delta HEL1/\Delta HEL1}$ mice and ESCs suggests that the DExD/H domain also has an important function in thermodynamic sensing and strand selection after the substrate cleavage. Multiple factors ensure guide strand selection, including Dicer itself, TARBP2, and AGO proteins (Noland et al., 2011) and properties of the RNA duplex itself (Noland and Doudna, 2013). Our results imply that DExD/H contributes to sensing RNA duplex thermodynamic asymmetry in a similar but non-redundant fashion as TARBP2 and that this may be the major function of DExD/H post-cleavage.

The high conservation of the mammalian DExD/H domain thus may originate from the need to preserve structural integrity of the domain to perform its non-canonical role in miRNA biogenesis while its ATPase activity became irrelevant. Substitutions of the conserved amino acid residues in the DExD/H domain, which mediate interaction with the RNase IIIb domain, increase Dicer's RNAi activity in cultured cells to the level achieved with the ΔHEL1 mutant (Figure 4F). It suggests that these mutations destabilize the closed state of Dicer and shift the equilibrium toward the open state. Notably, the K70N (GNT) mutation had measurable effects on miRNome as the abundance of miR-7068 mirtron, the most upregulated miRNA in *Dicer*$^{\Delta HEL1/\Delta HEL1}$ E15.5 embryos, increased 2-fold in *Dicer*$^{GNT/GNT}$ E15.5 embryos (Figures 1E and 1H). These data support the notion that Dicer function is sensitive to the structural integrity of the domain.

Structural analyses of Dicer-RNA complexes from plants and animals showed that there are subtle variations in the RNA ends recognition by the PAZ-platform domain, miRNA length measurement, and strand-biased cleavage (Liu et al., 2018; Sinha et al., 2018; Wei et al., 2021; Jourevleva et al., 2022; Wang et al., 2021). However, there are fundamental differences in how Dicer employs its helicase domain in different model species. DCR-1 from *Drosophila* also exists in an equilibrium between closed and open conformations but utilizes a conformational selection mechanism in which a rare, open conformation recognizes authentic pre-miRNA (Jourevleva et al., 2022). This contrasts with mammalian miRNA biogenesis in which the closed state of Dicer forms a stable pre-cleavage complex incorporating pre-miRNA architecture. Furthermore, the helicase domain in DCR-1 in *Drosophila* exhibits relatively low flexibility at different stages of miRNA processing when compared with the open state of mammalian Dicer where the unlocked helicase domain is highly flexible. Cryo-EM structures of DCR-2 from *Drosophila* and DCL1 from *Arabidopsis* showed that the dsRNA substrates are threaded through the helicase domain in ATP-dependent fashion and that the helicase domain clamps dsRNA (Sinha et al., 2018; Wei et al., 2021).

In contrast, our cryo-EM data show that murine Dicer supports miRNA biogenesis by the aforementioned two-step mechanism: (1) Dicer locked in the closed state recognizes a miRNA precursor and forms the pre-cleavage state and (2) Dicer switches into the open state that allows loading of the substrate into the catalytic site of Dicer. TARBP2 is able to shift the equilibrium from the closed toward the open state of Dicer as suggested by capturing the cleavage state in the presence of TARBP2 but not in its absence. Alternatively, TARBP2 can support the formation of the cleavage state by facilitating the accommodation of pre-miRNA into a rare occurring open state.

The DExD/H helicase appears to be involved only in the first step to recognize the substrate. In the second step the helicase dissociates from the core of Dicer to enable formation of the cleavage state. The first step of this mechanism appears to be a common feature of mammalian Dicers (Liu et al., 2018), whereas the second step is consistent with cryo-EM data for DCL-3-siRNA (Wang et al., 2021) and with suggestion that TARBP2 may facilitate conformational changes in human Dicer upon RNA binding (Taylor et al., 2013).

Our model also explains how absence of DExD/H activates efficient siRNA biogenesis (Figure 7A). However, the lethal phenotype of *Dicer*$^{\Delta HEL1/\Delta HEL1}$ mice demonstrates that Dicer^{O} cannot substitute the full-length Dicer *in vivo* because Dicer^{O} does not support miRNA biogenesis equally well. This implies caution and careful assessment of miRNome remodeling should a truncated Dicer variant be considered a therapeutic agent, such as the proposed gene therapy for Dicer deficiency in macular degeneration based on an N-terminally truncated Dicer variant termed OptiDicer (Wright et al., 2020). This may also explain why biologically important endogenous RNAi may have evolved in mouse oocytes where miRNAs are biologically irrelevant (Ma et al., 2010; Suh et al., 2010) and why this mechanism of

activation of RNAi pathway did not occur more frequently during mammalian evolution.

Limitations of the study

Analysis of small RNAs in ΔHEL1 mutant mice and ESCs revealed increased abundance of mirtrons, biased strand selection, and altered terminal nucleotide fidelity. However, pre-cleavage, cleavage, and post-cleavage effects could only be partially distinguished from the RNA-seq data. RNA-seq data suggest that the DExD/H domain also functions in thermodynamic sensing and strand selection. This notion is supported by similar effects observed for a set of miRNAs in ΔHEL1 and *Tarbp2* mutants. There are two possible scenarios to be examined: (1) DExD/H and TARBP2 have similar but independent functions in restricting thermodynamic sensing of the 5′ end of a set of 3p miRNAs. The loss of either DExD/H or TARBP2 is then sufficient to shift the balance toward 3p strand loading. (2) DExD/H and TARBP2 are functionally coupled in restricting the thermodynamic sensing; hence, the loss of either DExD/H or TARBP2 disrupts this functional coupling and yields increased 3p strand loading. However, this function cannot be resolved using existing RNA-seq and structural data. Furthermore, additional structures are needed. First, higher resolution of Dicer in the cleavage state is needed to reveal additional important structural details. Second, the post-cleavage structures of Dicer will shed light on the release of cleavage products and strand selection associated with the RISC-loading complex, where the DExD/H domain also appears to play a role.

STAR★METHODS

Detailed methods are provided in the online version of this paper and include the following:

- KEY RESOURCES TABLE
- RESOURCE AVAILABILITY
  - Lead contact
  - Materials availability
  - Data and code availability
- EXPERIMENTAL MODEL AND SUBJECT DETAILS
  - Animals
  - Cell culture and transfection
- METHOD DETAILS
  - Phenotype analyses
  - RNAi activity in cultured cells assay
  - Western blotting
  - Immunoprecipitation
  - RNA sequencing
  - Bioinformatic analyses
  - Luciferase assay
  - Recombinant plasmid preparation
  - Preparation of recombinant proteins
  - *In vitro* cleavage assay
  - *In vitro* reconstitution of the Dicer–pre-miR-15a complex
  - Cryo-EM specimen preparation and data acquisition
  - Image processing of electron micrographs
  - Cryo-EM model building and refinement
  - Data visualization
- QUANTIFICATION AND STATISTICAL ANALYSIS

SUPPLEMENTAL INFORMATION

Supplemental information can be found online at https://doi.org/10.1016/j.molcel.2022.10.010.

ACKNOWLEDGMENTS

We thank Kristian Vlahovicek (University of Zagreb) for support of bioinformatics analyses and Vladimir Benes (EMBL Sequencing Facility) and Genomics and Bioinformatics Core Facility at the Institute of Molecular Genetics for help with RNA sequencing. The main funding was provided by the Czech Science Foundation (EXPRO grant 20-03950X to P.S. and 22-19896S to R. Stefl). Early stages of the work were supported by European Research Council grants under the European Union's Horizon 2020 Research and Innovation Programme (grants 647403 to P.S. and 649030 to R. Stefl). V.B., D.F.J., and F.H. were in part supported by PhD student fellowships from the Charles University; this work will be in part fulfilling requirements for a PhD degree as "school work." Funding of D.Z. included the OP RDE project "Internal Grant Agency of Masaryk University" no. CZ.02.2.69/0.0/0.0/19_073/0016943. The Ministry of Education, Youth, and Sports of the Czech Republic (MEYS CR) provided institutional support for CEITEC 2020 project LQ1601. For technical support, we acknowledge EMBL Monterotondo's genome engineering and transgenic core facilities, the Czech Centre for Phenogenomics at the Institute of Molecular Genetics (supported by RVO 68378050 from the Czech Academy of Sciences and LM2018126 and CZ.02.1.01/0.0/0.0/18_046/0015861 CCP Infrastructure Upgrade II from MEYS CR), the Cryo-EM and Proteomics Core Facilities (CEITEC, Masaryk University) supported by the CIISB research infrastructure (LM2018127 from MEYS CR), and support from the Scientific Service Units of ISTA through resources from the Electron Microscopy Facility. Computational resources included e-Infrastruktura CZ (LM2018140) and ELIXIR-CZ (LM2018131) projects by MEYS CR and the Croatian National Centres of Research Excellence in Personalized Healthcare (#KK.01.1.1.01.0010) and Data Science and Advanced Cooperative Systems (#KK.01.1.1.01.0009) projects funded by the European Structural and Investment Funds grants.

AUTHOR CONTRIBUTIONS

Conceptualization, D.O'C., R. Stefl, and P.S.; investigation, D.Z., E.T., J. Pasulka, R.M., K.K., M.Z., C.M., M.S., V.B., F.H., I.J., M. Prochazkova, J. Prochazka, M. Pinkas, J.N., D.F.J., and C.B.; visualization, D.Z., J. Pasulka, J. Prochazka, F.H., R. Stefl, and P.S.; funding acquisition, D.Z., R. Sedlacek, R. Stefl, and P.S.; project administration, J.N., R. Sedlacek, D.O'C., R. Stefl, and P.S.; supervision, K.K., M.S., J.N., R. Sedlacek, D.O'C., R. Stefl, and P.S.; writing – original draft, R. Stefl and P.S.; writing – review & editing, D.Z., E.T., J. Pasulka, F.H., I.J., J. Prochazka, R.M., D.O'C., C.B., R. Stefl, and P.S.

DECLARATION OF INTERESTS

The authors declare no competing interests.

Received: April 21, 2022
Revised: July 21, 2022
Accepted: October 7, 2022
Published: November 3, 2022

REFERENCES

Anglesio, M.S., Wang, Y., Yang, W., Senz, J., Wan, A., Heravi-Moussavi, A., Salamanca, C., Maines-Bandiera, S., Huntsman, D.G., and Morin, G.B. (2013). Cancer-associated somatic DICER1 hotspot mutations cause defective miRNA processing and reverse-strand expression bias to predominantly mature 3p strands through loss of 5p strand cleavage. J. Pathol. *229*, 400–409. https://doi.org/10.1002/path.4135.

Antczak, M., Popenda, M., Zok, T., Sarzynska, J., Ratajczak, T., Tomczyk, K., Adamiak, R.W., and Szachniuk, M. (2016). New functionality of RNAComposer: an application to shape the axis of miR160 precursor structure. Acta Biochim. Pol. *63*, 737–744. https://doi.org/10.18388/abp.2016_1329.

Bartel, D.P. (2018). Metazoan microRNAs. Cell *173*, 20–51. https://doi.org/10.1016/j.cell.2018.03.006.

Bepler, T., Morin, A., Rapp, M., Brasch, J., Shapiro, L., Noble, A.J., and Berger, B. (2019). Positive-unlabeled convolutional neural networks for particle picking in cryo-electron micrographs. Nat. Methods *16*, 1153–1160. https://doi.org/10.1038/s41592-019-0575-8.

Berezikov, E., Chung, W.J., Willis, J., Cuppen, E., and Lai, E.C. (2007). Mammalian mirtron genes. Mol. Cell *28*, 328–336. https://doi.org/10.1016/j.molcel.2007.09.028.

Berman, H., Henrick, K., and Nakamura, H. (2003). Announcing the worldwide Protein Data Bank. Nat Struct Mol Biol *10*, 980. https://doi.org/10.1038/nsb1203-980.

Brennecke, J., Stark, A., Russell, R.B., and Cohen, S.M. (2005). Principles of microRNA-target recognition. PLoS Biol. *3*, e85.

Campo-Paysaa, F., Sémon, M., Cameron, R.A., Peterson, K.J., and Schubert, M. (2011). microRNA complements in deuterostomes: origin and evolution of microRNAs. Evol. Dev. *13*, 15–27. https://doi.org/10.1111/j.1525-142X.2010.00452.x.

Cenik, E.S., Fukunaga, R., Lu, G., Dutcher, R., Wang, Y., Tanaka Hall, T.M., and Zamore, P.D. (2011). Phosphate and R2D2 restrict the substrate specificity of Dicer-2, an ATP-driven ribonuclease. Mol. Cell *42*, 172–184. https://doi.org/10.1016/j.molcel.2011.03.002.

Chendrimada, T.P., Gregory, R.I., Kumaraswamy, E., Norman, J., Cooch, N., Nishikura, K., and Shiekhattar, R. (2005). TRBP recruits the Dicer complex to Ago2 for microRNA processing and gene silencing. Nature *436*, 740–744. https://doi.org/10.1038/nature03868.

Chiang, H.R., Schoenfeld, L.W., Ruby, J.G., Auyeung, V.C., Spies, N., Baek, D., Johnston, W.K., Russ, C., Luo, S., Babiarz, J.E., et al. (2010). Mammalian microRNAs: experimental evaluation of novel and previously annotated genes. Genes Dev. *24*, 992–1009. https://doi.org/10.1101/gad.1884710.

Comazzetto, S., Di Giacomo, M., Rasmussen, K.D., Much, C., Azzi, C., Perlas, E., Morgan, M., and O'Carroll, D. (2014). Oligoasthenoteratozoospermia and infertility in mice deficient for miR-34b/c and miR-449 loci. PLoS Genet. *10*. e1004597. https://doi.org/10.1371/journal.pgen.1004597.

Cordin, O., Banroques, J., Tanner, N.K., and Linder, P. (2006). The DEAD-box protein family of RNA helicases. Gene *367*, 17–37. https://doi.org/10.1016/j.gene.2005.10.019.

Croll, T.I. (2018). Isolde: a physically realistic environment for model building into low-resolution electron-density maps. Acta Crystallogr. D Struct. Biol. *74*, 519–530. https://doi.org/10.1107/S2059798318002425.

Davis, I.W., Leaver-Fay, A., Chen, V.B., Block, J.N., Kapral, G.J., Wang, X., Murray, L.W., Arendall, W.B., Snoeyink, J., Richardson, J.S., et al. (2007). MolProbity: all-atom contacts and structure validation for proteins and nucleic acids. Nucleic Acids Res *35*, W375–W383. https://doi.org/10.1093/nar/gkm216.

Davis, I.W., Murray, L.W., Richardson, J.S., and Richardson, D.C. (2004). MOLPROBITY: structure validation and all-atom contact analysis for nucleic acids and their complexes. Nucleic Acids Res *32*, W615–W619. https://doi.org/10.1093/nar/gkh398.

Demeter, T., Vaskovicova, M., Malik, R., Horvat, F., Pasulka, J., Svobodova, E., Flemr, M., and Svoboda, P. (2019). Main constraints for RNAi induced by expressed long dsRNA in mouse cells. Life Sci. Alliance *2*, 2. https://doi.org/10.26508/lsa.201800289.

Dobin, A., Davis, C.A., Schlesinger, F., Drenkow, J., Zaleski, C., Jha, S., Batut, P., Chaisson, M., and Gingeras, T.R. (2013). STAR: ultrafast universal RNA-seq aligner. Bioinformatics *29*, 15–21. https://doi.org/10.1093/bioinformatics/bts635.

Emsley, P., Lohkamp, B., Scott, W.G., and Cowtan, K. (2010). Features and development of coot. Acta Crystallogr. D Biol. Crystallogr. *66*, 486–501. https://doi.org/10.1107/S0907444910007493.

Fairman-Williams, M.E., Guenther, U.P., and Jankowsky, E. (2010). SF1 and SF2 helicases: family matters. Curr. Opin. Struct. Biol. *20*, 313–324. https://doi.org/10.1016/j.sbi.2010.03.011.

Farley, F.W., Soriano, P., Steffen, L.S., and Dymecki, S.M. (2000). Widespread recombinase expression using FLPeR (flipper) mice. Genesis *28*, 106–110.

Flemr, M., Malik, R., Franke, V., Nejepinska, J., Sedlacek, R., Vlahovicek, K., and Svoboda, P. (2013). A retrotransposon-driven dicer isoform directs endogenous small interfering RNA production in mouse oocytes. Cell *155*, 807–816. https://doi.org/10.1016/j.cell.2013.10.001.

Frankish, A., Diekhans, M., Ferreira, A.M., Johnson, R., Jungreis, I., Loveland, J., Mudge, J.M., Sisu, C., Wright, J., Armstrong, J., et al. (2019). GENCODE reference annotation for the human and mouse genomes. Nucleic Acids Res. *47*, D766–D773. https://doi.org/10.1093/nar/gky955.

Fromm, B., Høye, E., Domanska, D., Zhong, X., Aparicio-Puerta, E., Ovchinnikov, V., Umu, S.U., Chabot, P.J., Kang, W., Aslanzadeh, M., et al. (2022). MirGeneDB 2.1: toward a complete sampling of all major animal phyla. Nucleic Acids Res. *50*, D204–D210. https://doi.org/10.1093/nar/gkab1101.

Hampf, M., and Gossen, M. (2006). A protocol for combined Photinus and Renilla luciferase quantification compatible with protein assays. Anal. Biochem. *356*, 94–99. https://doi.org/10.1016/j.ab.2006.04.046.

Jia, H., Kolaczkowski, O., Rolland, J., and Kolaczkowski, B. (2017). Increased affinity for RNA targets evolved early in animal and plant dicer lineages through different structural mechanisms. Mol. Biol. Evol. *34*, 3047–3063. https://doi.org/10.1093/molbev/msx187.

Jourevleva, K., Golovenko, D., Demo, G., Dutcher, R.C., Hall, T.M.T., Zamore, P.D., and Korostelev, A.A. (2022). Structural basis of microRNA biogenesis by Dicer-1 and its partner protein Loqs-PB. Mol. Cell *82*, 4049–4063.

Jumper, J., Evans, R., Pritzel, A., Green, T., Figurnov, M., Ronneberger, O., Tunyasuvunakool, K., Bates, R., Žídek, A., Potapenko, A., et al. (2021). Highly accurate protein structure prediction with AlphaFold. Nature *596*, 583–589. https://doi.org/10.1038/s41586-021-03819-2.

Kennedy, E.M., Whisnant, A.W., Kornepati, A.V., Marshall, J.B., Bogerd, H.P., and Cullen, B.R. (2015). Production of functional small interfering RNAs by an amino-terminal deletion mutant of human Dicer. Proc. Natl. Acad. Sci. USA *112*, E6945–E6954. https://doi.org/10.1073/pnas.1513421112.

Kent, W.J., Zweig, A.S., Barber, G., Hinrichs, A.S., and Karolchik, D. (2010). BigWig and BigBed: enabling browsing of large distributed datasets. Bioinformatics *26*, 2204–2207.

Ketting, R.F. (2011). The many faces of RNAi. Dev. Cell *20*, 148–161. https://doi.org/10.1016/j.devcel.2011.01.012.

Ketting, R.F., Fischer, S.E., Bernstein, E., Sijen, T., Hannon, G.J., and Plasterk, R.H. (2001). Dicer functions in RNA interference and in synthesis of small RNA involved in developmental timing in C. elegans. Genes Dev. *15*, 2654–2659. https://doi.org/10.1101/gad.927801.

Kozomara, A., Birgaoanu, M., and Griffiths-Jones, S. (2019). miRBase: from microRNA sequences to function. Nucleic Acids Res. *47*, D155–D162. https://doi.org/10.1093/nar/gky1141.

Ladewig, E., Okamura, K., Flynt, A.S., Westholm, J.O., and Lai, E.C. (2012). Discovery of hundreds of mirtrons in mouse and human small RNA data. Genome Res. *22*, 1634–1645. https://doi.org/10.1101/gr.133553.111.

Lau, P.W., Guiley, K.Z., De, N., Potter, C.S., Carragher, B., and MacRae, I.J. (2012). The molecular architecture of human Dicer. Nat. Struct. Mol. Biol. *19*, 436–440. https://doi.org/10.1038/nsmb.2268.

Lau, P.W., Potter, C.S., Carragher, B., and MacRae, I.J. (2009). Structure of the human Dicer-TRBP complex by electron microscopy. Structure *17*, 1326–1332. https://doi.org/10.1016/j.str.2009.08.013.

Lewis, B.P., Shih, I.H., Jones-Rhoades, M.W., Bartel, D.P., and Burge, C.B. (2003). Prediction of mammalian microRNA targets. Cell *115*, 787–798.

Liao, Y., Smyth, G.K., and Shi, W. (2014). featureCounts: an efficient general purpose program for assigning sequence reads to genomic features. Bioinformatics *30*, 923–930. https://doi.org/10.1093/bioinformatics/btt656.

Liebschner, D., Afonine, P.V., Baker, M.L., Bunkóczi, G., Chen, V.B., Croll, T.I., Hintze, B., Hung, L.W., Jain, S., McCoy, A.J., et al. (2019). Macromolecular structure determination using X-rays, neutrons and electrons: recent developments in Phenix. Acta Crystallogr. D Struct. Biol. *75*, 861–877. https://doi.org/10.1107/S2059798319011471.

Lingel, A., Simon, B., Izaurralde, E., and Sattler, M. (2003). Structure and nucleic-acid binding of the Drosophila Argonaute 2 PAZ domain. Nature *426*, 465–469. https://doi.org/10.1038/nature02123.

Liu, Q., Rand, T.A., Kalidas, S., Du, F., Kim, H.E., Smith, D.P., and Wang, X. (2003). R2D2, a bridge between the initiation and effector steps of the Drosophila RNAi pathway. Science *301*, 1921–1925. https://doi.org/10.1126/science.1088710.

Liu, Z., Wang, J., Cheng, H., Ke, X., Sun, L., Zhang, Q.C., and Wang, H.W. (2018). Cryo-EM structure of human dicer and its complexes with a pre-miRNA substrate. Cell *173*, 1191–1203.e12. https://doi.org/10.1016/j.cell.2018.03.080.

Love, M.I., Huber, W., and Anders, S. (2014). Moderated estimation of fold change and dispersion for RNA-seq data with DESeq2. Genome Biol. *15*, 550. https://doi.org/10.1186/s13059-014-0550-8.

Ma, E., MacRae, I.J., Kirsch, J.F., and Doudna, J.A. (2008). Autoinhibition of human dicer by its internal helicase domain. J. Mol. Biol. *380*, 237–243. https://doi.org/10.1016/j.jmb.2008.05.005.

Ma, J., Flemr, M., Stein, P., Berninger, P., Malik, R., Zavolan, M., Svoboda, P., and Schultz, R.M. (2010). MicroRNA activity is suppressed in mouse oocytes. Curr. Biol. *20*, 265–270.

MacRae, I.J., Zhou, K., Li, F., Repic, A., Brooks, A.N., Cande, W.Z., Adams, P.D., and Doudna, J.A. (2006). Structural basis for double-stranded RNA processing by Dicer. Science *311*, 195–198. https://doi.org/10.1126/science.1121638.

Marini, F., and Binder, H. (2019). pcaExplorer: an R/Bioconductor package for interacting with RNA-seq principal components. BMC Bioinformatics *20*, 331. https://doi.org/10.1186/s12859-019-2879-1.

Martin, M. (2011). Cutadapt removes adapter sequences from high-throughput sequencing reads. EMBnet. j. *17*, 3. https://doi.org/10.14806/ej.17.1.200.

Mastronarde, D.N. (2005). Automated electron microscope tomography using robust prediction of specimen movements. J Struct Biol *152*, 36–51. https://doi.org/10.1016/j.jsb.2005.07.007.

Medley, J.C., Panzade, G., and Zinovyeva, A.Y. (2021). microRNA strand selection: unwinding the rules. Wiley Interdiscip. Rev. RNA *12*, e1627. https://doi.org/10.1002/wrna.1627.

Mencía, A., Modamio-Høybjør, S., Redshaw, N., Morín, M., Mayo-Merino, F., Olavarrieta, L., Aguirre, L.A., del Castillo, I., Steel, K.P., Dalmay, T., et al. (2009). Mutations in the seed region of human miR-96 are responsible for nonsyndromic progressive hearing loss. Nat. Genet. *41*, 609–613. https://doi.org/10.1038/ng.355.

Meunier, J., Lemoine, F., Soumillon, M., Liechti, A., Weier, M., Guschanski, K., Hu, H., Khaitovich, P., and Kaessmann, H. (2013). Birth and expression evolution of mammalian microRNA genes. Genome Res. *23*, 34–45. https://doi.org/10.1101/gr.140269.112.

Murchison, E.P., Stein, P., Xuan, Z., Pan, H., Zhang, M.Q., Schultz, R.M., and Hannon, G.J. (2007). Critical roles for Dicer in the female germline. Genes Dev. *21*, 682–693.

Nagy, A., Rossant, J., Nagy, R., Abramow-Newerly, W., and Roder, J.C. (1993). Derivation of completely cell culture-derived mice from early-passage embryonic stem cells. Proc. Natl. Acad. Sci. USA *90*, 8424–8428. https://doi.org/10.1073/pnas.90.18.8424.

Nejepinska, J., Malik, R., Filkowski, J., Flemr, M., Filipowicz, W., and Svoboda, P. (2012). dsRNA expression in the mouse elicits RNAi in oocytes and low adenosine deamination in somatic cells. Nucleic Acids Res. *40*, 399–413. https://doi.org/10.1093/nar/gkr702.

Noland, C.L., and Doudna, J.A. (2013). Multiple sensors ensure guide strand selection in human RNAi pathways. RNA *19*, 639–648. https://doi.org/10.1261/rna.037424.112.

Noland, C.L., Ma, E., and Doudna, J.A. (2011). siRNA repositioning for guide strand selection by human Dicer complexes. Mol. Cell *43*, 110–121. https://doi.org/10.1016/j.molcel.2011.05.028.

Ota, H., Sakurai, M., Gupta, R., Valente, L., Wulff, B.E., Ariyoshi, K., Iizasa, H., Davuluri, R.V., and Nishikura, K. (2013). ADAR1 forms a complex with Dicer to promote microRNA processing and RNA-induced gene silencing. Cell *153*, 575–589. https://doi.org/10.1016/j.cell.2013.03.024.

Park, C.Y., Choi, Y.S., and McManus, M.T. (2010). Analysis of microRNA knockouts in mice. Hum. Mol. Genet. *19*, R169–R175. https://doi.org/10.1093/hmg/ddq367.

Paturi, S., and Deshmukh, M.V. (2021). A glimpse of "dicer biology" Through the structural and functional perspective. Front. Mol. Biosci. *8*, 643657. https://doi.org/10.3389/fmolb.2021.643657.

Pettersen, E.F., Goddard, T.D., Huang, C.C., Couch, G.S., Greenblatt, D.M., Meng, E.C., and Ferrin, T.E. (2004). UCSF Chimera–a visualization system for exploratory research and analysis. J. Comput. Chem. *25*, 1605–1612. https://doi.org/10.1002/jcc.20084.

Pettersen, E.F., Goddard, T.D., Huang, C.C., Meng, E.C., Couch, G.S., Croll, T.I., Morris, J.H., and Ferrin, T.E. (2021). UCSF ChimeraX: structure visualization for researchers, educators, and developers. Protein Sci. *30*, 70–82. https://doi.org/10.1002/pro.3943.

Popenda, M., Szachniuk, M., Antczak, M., Purzycka, K.J., Lukasiak, P., Bartol, N., Blazewicz, J., and Adamiak, R.W. (2012). Automated 3D structure composition for large RNAs. Nucleic Acids Res. *40*, e112. https://doi.org/10.1093/nar/gks339.

Poueymirou, W.T., Auerbach, W., Frendewey, D., Hickey, J.F., Escaravage, J.M., Esau, L., Doré, A.T., Stevens, S., Adams, N.C., Dominguez, M.G., et al. (2007). F0 generation mice fully derived from gene-targeted embryonic stem cells allowing immediate phenotypic analyses. Nat. Biotechnol. *25*, 91–99. https://doi.org/10.1038/nbt1263.

Provost, P., Dishart, D., Doucet, J., Frendewey, D., Samuelsson, B., and Rådmark, O. (2002). Ribonuclease activity and RNA binding of recombinant human Dicer. EMBO J. *21*, 5864–5874. https://doi.org/10.1093/emboj/cdf578.

Pullagura, S.R.N., Buaas, B., Gray, N., Krening, L.C., Srivastava, A., and Braun, R.E. (2018). Functional redundancy of DICER cofactors TARBP2 and PRKRA During murine embryogenesis does not involve miRNA biogenesis. Genetics *208*, 1513–1522. https://doi.org/10.1534/genetics.118.300791.

Punjani, A., Rubinstein, J.L., Fleet, D.J., and Brubaker, M.A. (2017). cryoSPARC: algorithms for rapid unsupervised cryo-EM structure determination. Nat. Methods *14*, 290–296. https://doi.org/10.1038/nmeth.4169.

Ran, F.A., Hsu, P.D., Wright, J., Agarwala, V., Scott, D.A., and Zhang, F. (2013). Genome engineering using the CRISPR-Cas9 system. Nat. Protoc. *8*, 2281–2308. https://doi.org/10.1038/nprot.2013.143.

Scheres, S.H. (2012). RELION: implementation of a Bayesian approach to cryo-EM structure determination. J. Struct. Biol. *180*, 519–530. https://doi.org/10.1016/j.jsb.2012.09.006.

Scheres, S.H.W. (2016). Processing of Structurally Heterogeneous Cryo-EM Data in RELION. Methods Enzymol *579*, 125–157. https://doi.org/10.1016/BS.MIE.2016.04.012.

Schwenk, F., Baron, U., and Rajewsky, K. (1995). A cre-transgenic mouse strain for the ubiquitous deletion of loxP-flanked gene segments including deletion in germ cells. Nucleic Acids Res. *23*, 5080–5081. https://doi.org/10.1093/nar/23.24.5080.

Sinha, N.K., Iwasa, J., Shen, P.S., and Bass, B.L. (2018). Dicer uses distinct modules for recognizing dsRNA termini. Science *359*, 329–334. https://doi.org/10.1126/science.aaq0921.

Smit, A.F.A., Hubley, R., and Green, P. (2013–2015). RepeatMasker Open-4.0. http://www.repeatmasker.org.

CellPress
OPEN ACCESS

Song, J.J., Liu, J., Tolia, N.H., Schneiderman, J., Smith, S.K., Martienssen, R.A., Hannon, G.J., and Joshua-Tor, L. (2003). The crystal structure of the Argonaute2 PAZ domain reveals an RNA binding motif in RNAi effector complexes. Nat. Struct. Biol. *10*, 1026–1032. https://doi.org/10.1038/nsb1016.

Stefl, R., Oberstrass, F.C., Hood, J.L., Jourdan, M., Zimmermann, M., Skrisovska, L., Maris, C., Peng, L., Hofr, C., Emeson, R.B., and Allain, F.H. (2010). The solution structure of the ADAR2 dsRBM-RNA complex reveals a sequence-specific readout of the minor groove. Cell *143*, 225–237. https://doi.org/10.1016/j.cell.2010.09.026.

Stefl, R., Skrisovska, L., and Allain, F.H. (2005). RNA sequence- and shape-dependent recognition by proteins in the ribonucleoprotein particle. EMBO Rep. *6*, 33–38. https://doi.org/10.1038/sj.embor.7400325.

Stein, P., Rozhkov, N.V., Li, F., Cárdenas, F.L., Davydenko, O., Vandivier, L.E., Gregory, B.D., Hannon, G.J., and Schultz, R.M. (2015). Essential Role for endogenous siRNAs during meiosis in mouse oocytes. PLoS Genet. *11*. e1005013. https://doi.org/10.1371/journal.pgen.1005013.

Suh, N., Baehner, L., Moltzahn, F., Melton, C., Shenoy, A., Chen, J., and Blelloch, R. (2010). MicroRNA function is globally suppressed in mouse oocytes and early embryos. Curr. Biol. *20*, 271–277.

Taborska, E., Pasulka, J., Malik, R., Horvat, F., Jenickova, I., Jelić Matošević, Z., and Svoboda, P. (2019). Restricted and non-essential redundancy of RNAi and piRNA pathways in mouse oocytes. PLoS Genet. *15*. e1008261. https://doi.org/10.1371/journal.pgen.1008261.

Tam, O.H., Aravin, A.A., Stein, P., Girard, A., Murchison, E.P., Cheloufi, S., Hodges, E., Anger, M., Sachidanandam, R., Schultz, R.M., and Hannon, G.J. (2008). Pseudogene-derived small interfering RNAs regulate gene expression in mouse oocytes. Nature *453*, 534–538.

Tang, F., Kaneda, M., O'Carroll, D., Hajkova, P., Barton, S.C., Sun, Y.A., Lee, C., Tarakhovsky, A., Lao, K., and Surani, M.A. (2007). Maternal microRNAs are essential for mouse zygotic development. Genes Dev. *21*, 644–648.

Tang, G., Peng, L., Baldwin, P.R., Mann, D.S., Jiang, W., Rees, I., and Ludtke, S.J. (2007). EMAN2: an extensible image processing suite for electron microscopy. J. Struct. Biol. *157*, 38–46. https://doi.org/10.1016/j.jsb.2006.05.009.

Taylor, D.W., Ma, E., Shigematsu, H., Cianfrocco, M.A., Noland, C.L., Nagayama, K., Nogales, E., Doudna, J.A., and Wang, H.W. (2013). Substrate-specific structural rearrangements of human Dicer. Nat. Struct. Mol. Biol. *20*, 662–670. https://doi.org/10.1038/nsmb.2564.

Tian, Y., Simanshu, D.K., Ma, J.B., Park, J.E., Heo, I., Kim, V.N., and Patel, D.J. (2014). A phosphate-binding pocket within the platform-PAZ-connector helix cassette of human Dicer. Mol. Cell *53*, 606–616. https://doi.org/10.1016/j.molcel.2014.01.003.

Toni, L.S., Garcia, A.M., Jeffrey, D.A., Jiang, X., Stauffer, B.L., Miyamoto, S.D., and Sucharov, C.C. (2018). Optimization of phenol-chloroform RNA extraction. MethodsX *5*, 599–608. https://doi.org/10.1016/j.mex.2018.05.011.

Tsutsumi, A., Kawamata, T., Izumi, N., Seitz, H., and Tomari, Y. (2011). Recognition of the pre-miRNA structure by Drosophila Dicer-1. Nat. Struct. Mol. Biol. *18*, 1153–1158. https://doi.org/10.1038/nsmb.2125.

Varadi, M., Anyango, S., Deshpande, M., Nair, S., Natassia, C., Yordanova, G., Yuan, D., Stroe, O., Wood, G., Laydon, A., et al. (2022). AlphaFold Protein Structure Database: massively expanding the structural coverage of protein-sequence space with high-accuracy models. Nucleic Acids Res *50*, D439–D444. https://doi.org/10.1093/NAR/GKAB1061.

Wagner, T., Merino, F., Stabrin, M., Moriya, T., Antoni, C., Apelbaum, A., Hagel, P., Sitsel, O., Raisch, T., Prumbaum, D., et al. (2019). SPHIRE-crYOLO is a fast and accurate fully automated particle picker for cryo-EM. Commun. Biol. *2*, 218. https://doi.org/10.1038/s42003-019-0437-z.

Wang, Q., Xue, Y., Zhang, L., Zhong, Z., Feng, S., Wang, C., Xiao, L., Yang, Z., Harris, C.J., Wu, Z., et al. (2021). Mechanism of siRNA production by a plant Dicer-RNA complex in dicing-competent conformation. Science *374*, 1152–1157. https://doi.org/10.1126/science.abl4546.

Wang, Z., Hartman, E., Roy, K., Chanfreau, G., and Feigon, J. (2011). Structure of a yeast RNase III dsRBD complex with a noncanonical RNA substrate provides new insights into binding specificity of dsRBDs. Structure *19*, 999–1010. https://doi.org/10.1016/j.str.2011.03.022.

Watanabe, T., Totoki, Y., Toyoda, A., Kaneda, M., Kuramochi-Miyagawa, S., Obata, Y., Chiba, H., Kohara, Y., Kono, T., Nakano, T., et al. (2008). Endogenous siRNAs from naturally formed dsRNAs regulate transcripts in mouse oocytes. Nature *453*, 539–543.

Wei, X., Ke, H., Wen, A., Gao, B., Shi, J., and Feng, Y. (2021). Structural basis of microRNA processing by Dicer-like 1. Nat. Plants *7*, 1389–1396. https://doi.org/10.1038/s41477-021-01000-1.

Welker, N.C., Maity, T.S., Ye, X., Aruscavage, P.J., Krauchuk, A.A., Liu, Q., and Bass, B.L. (2011). Dicer's helicase domain discriminates dsRNA termini to promote an altered reaction mode. Mol. Cell *41*, 589–599. https://doi.org/10.1016/j.molcel.2011.02.005.

Williams, C.J., Headd, J.J., Moriarty, N.W., Prisant, M.G., Videau, L.L., Deis, L.N., Verma, V., Keedy, D.A., Hintze, B.J., Chen, V.B., et al. (2018). MolProbity: More and better reference data for improved all-atom structure validation. Protein Science *27*, 293–315. https://doi.org/10.1002/pro.3330.

Wilson, R.C., Tambe, A., Kidwell, M.A., Noland, C.L., Schneider, C.P., and Doudna, J.A. (2015). Dicer-TRBP complex formation ensures accurate mammalian microRNA biogenesis. Mol. Cell *57*, 397–407.

Wright, C.B., Uehara, H., Kim, Y., Yasuma, T., Yasuma, R., Hirahara, S., Makin, R.D., Apicella, I., Pereira, F., Nagasaka, Y., et al. (2020). Chronic Dicer1 deficiency promotes atrophic and neovascular outer retinal pathologies in mice. Proc. Natl. Acad. Sci. USA *117*, 2579–2587. https://doi.org/10.1073/pnas.1909761117.

Yan, K.S., Yan, S., Farooq, A., Han, A., Zeng, L., and Zhou, M.M. (2003). Structure and conserved RNA binding of the PAZ domain. Nature *426*, 468–474. https://doi.org/10.1038/nature02129.

Zhang, H., Kolb, F.A., Brondani, V., Billy, E., and Filipowicz, W. (2002). Human Dicer preferentially cleaves dsRNAs at their termini without a requirement for ATP. EMBO J. *21*, 5875–5885. https://doi.org/10.1093/emboj/cdf582.

Zhang, H., Kolb, F.A., Jaskiewicz, L., Westhof, E., and Filipowicz, W. (2004). Single processing center models for human Dicer and bacterial RNase III. Cell *118*, 57–68. https://doi.org/10.1016/j.cell.2004.06.017.

Zhang, K. (2016). Gctf: real-time CTF determination and correction. J. Struct. Biol. *193*, 1–12. https://doi.org/10.1016/j.jsb.2015.11.003.

Zheng, S.Q., Palovcak, E., Armache, J.P., Verba, K.A., Cheng, Y., and Agard, D.A. (2017). MotionCor2: anisotropic correction of beam-induced motion for improved cryo-electron microscopy. Nat. Methods *14*, 331–332. https://doi.org/10.1038/nmeth.4193.

STAR★METHODS

KEY RESOURCES TABLE

REAGENT or RESOURCE	SOURCE	IDENTIFIER
**Antibodies**		
α-HA Rat monoclonal antibody (clone 3F10)	Roche	Cat# 11867431001; RRID:AB_390919
α-HA	Cell Signaling	Cat# 3724
Anti-HA Magnetic Beads	ThermoFisher Scientific	Cat# 88836; RRID:AB_2749815
α-TUBA4A	Sigma-Aldrich	Cat# T6074; RRID:AB_477582
α-TARBP2	ThermoFisher Scientific	Cat# LF-MA0209; RRID:AB_1875916
α-FLAG	Sigma-Aldrich	Cat# F3165; RRID:AB_259529
mouse anti-rabbit IgG-HRP	Santa-Cruz	Cat# sc-2357; RRID:AB_628497
HRP-conjugated anti-mouse IgG binding protein	Santa-Cruz	Cat# sc-525409
goat anti-Rat IgG-HPR	ThermoFisher Scientific	Cat# 31470; RRID:AB_228356
**Bacterial and virus strains**		
One Shot™ TOP10 Chemically Competent cells	ThermoFisher Scientific	Cat# C404006
NEB 5-alpha Competent E. coli	New England Biolabs	part of Cat# E0554S
MAX Efficiency™ DH10Bac Competent Cells	ThermoFisher Scientific	Cat# 10361012
P1 virus	ThermoFisher Scientific	N/A
Sf9 cells in Sf-900™ II SFM	ThermoFisher Scientific	Cat# 11496015
High Five™ Cells in Express Five™ Medium	ThermoFisher Scientific	Cat# B85502
**Chemicals, peptides, and recombinant proteins**		
[γ-^{32}P]-ATP	HARTMANN ANALYTIC	tCat# FP-501
2-Mercaptoethanol (50 mM)	ThermoFisher Scientific	Cat# 31350010
30% Acrylamide/Bis Solution, 29:1	Bio-Rad	Cat# 161-0156
ALLin™ HiFi DNA Polymerase	HighQu	Cat# HLE0201
Benzonase® Nuclease	Sigma-Merck	Cat# E1014-25KU
CHIR-99021 (CT99021) HCl	Selleck Chemicals	Cat# S2924
DAPI	Sigma-Aldrich	Cat# 10236276001
Decade™ Markers System	ThermoFisher Scientific	Cat# AM7778
DMEM	Sigma-Aldrich	Cat# D6429
dNTP Mix (10 mM each)	ThermoFisher Scientific	Cat# R0192
Fetal Bovine Serum (FCS)	Sigma-Aldrich	Cat# F7524
FuGENE HD Transfection Reagent	Promega	Cat# E2311
Gelatin from cold water fish skin	Sigma-Aldrich	Cat# G7765
Immobilon-P PVDF Membrane	Sigma-Aldrich	Cat# IPVH00010
KnockOut DMEM	ThermoFisher Scientific	Cat# 10829018
L-Glutamin solution	Sigma-Aldrich	Cat# G7513
LIF	Isokine	Cat# 01-A1140-100
Lipofectamine 3000 Transfection Reagent	ThermoFisher Scientific	Cat# L3000015
Lugol's Iodine: Potassium iodide; Iodine	Penta	Cat# 7681-11-0<br>Cat# 17570-30500
MEM Non-Essential Amino Acids Solution (100X)	ThermoFisher Scientific	Cat# 11140068
Mirdametinib (PD0325901)	Selleck Chemicals	Cat# S1036
mouse Dicer^{SOM}	this paper	N/A

(*Continued on next page*)

Continued		
REAGENT or RESOURCE	SOURCE	IDENTIFIER
mouse Dicer$^{\Delta HEL1}$	this paper	N/A
NiNTA-agarose	Qiagen	Cat# 30210
Penicillin-Streptomycin	Sigma-Aldrich	Cat# P0781
PFA	Penta	Cat# 23700-31000
Pierce Anti-HA Magnetic Beads	ThermoFisher Scientific	Cat# 88836
Protease Inhibitor Cocktail Set III, Animal-Free	Sigma-Aldrich	Cat# 535140
Qiazol lysis reagent	Qiagen	Cat# 79306
RevertAid Reverse Transcriptase (200 U/μL)	ThermoFisher Scientific	Cat# EP0441
RNase Inhibitor, Murine	New England BioLabs	Cat# M0314L
SuperSignal™ West Femto Maximum Sensitivity Substrate	ThermoFisher Scientific	Cat# 34096
SYBR™ Green PCR Master Mix	ThermoFisher Scientific	Cat# 4309155
T4 Polynucleotide Kinase	New England BioLabs	Cat# M0201L
V-53D Diluent	Mindray	Cat# 105-000146-00
Vivaspin Turbo15	Sartorius	Cat# VS15T41
Critical commercial assays		
Click-it EdU Imaging Kit	ThermoFisher Scientific	Cat# C10337
Fuji imaging plate BAS-IP MS 2025	VWR	Cat# 28-9564-75
In Situ Cell Death Detection Kit, TMR red	Roche	Cat# 12156792910
Monarch® Genomic DNA Purification Kit	New England Biolabs	Cat# T1030S
NEBNext® Multiplex Small RNA Library Prep Set for Illumina®	NEB	Cat# E7300S
NEBNext® Ultra™ II RNA Library Prep Kit for Illumina®	NEB	Cat# E7760S
NEXTflex Small RNA-Seq Kit v3	BiooScientific	Cat# NOVA-5132-06
PCR Genotyping Kit	Top-Bio	Cat# D227
Protein Assay Kit I (Bradford assay)	Bio-Rad	Cat# 500-0006
Q5 Site-Directed Mutagenesis kit	New England Biolabs	Cat# E0554S
Ribo-Zero® plus rRNA depletion Kit	Illumina	Cat# 20040526
RNeasy Mini Kit	Qiagen	Cat# 74104
Strep-Tactin™XT Superflow™ High Capacity Resin	IBA Lifesciences	Cat# 2-4030-010
Superdex® 75 Increase 10/300 GL	Cytiva	Cat# 29-1487-21
Superose® 6 Increase 10/300 GL	Cytiva	Cat# 29-0915-96
X-ray film Blue	Cole-Parmer	Cat# 21700-03
Deposited data		
Coordinates of Arabidopsis DCL1 in complex with pre-miRNA 166f	Wei et al., 2021	PDB: 7ELE
Coordinates of Arabidopsis DCL3 in complex with a 40-bp RNA	Wang et al., 2021	PDB: 7VG2
Coordinates of human Dicer●TARBP2 complex	Liu et al., 2018	PDB: 5ZAK
Coordinates of human Dicer●TARBP2●pre-let-7 complex	Liu et al., 2018	PDB: 5ZAL
Coordinates of mouse Dicer	this paper, Table S4	PDB: 7YZ4
Coordinates of mouse Dicer in complex with pre-miR-15a	this paper, Table S4	PDB: 7YYM
Coordinates of mouse Dicer^{O} in complex with pre-miR-15a	this paper, Table S4	PDB: 7YYN

(*Continued on next page*)

Continued

REAGENT or RESOURCE	SOURCE	IDENTIFIER
Coordinates of mouse Dicer in complex with pre-miR-15a and TARBP2 (pre-cleavage)	this paper, Table S4	PDB: 7ZPK
Coordinates of mouse Dicer in complex with pre-miR-15a and TARBP2 (cleavage)	this paper, Table S4	PDB: 7ZPI
RNA-seq data	this paper, Table S5	GEO: GSE196310
Experimental models: Cell lines		
DicerX/X and Pkr-/- ESC strain	this paper	N/A
DicerX/X ESC strain	this paper	N/A
Hi5 cells	ThermoFisher Scientific	Cat# B85502
Human osteosarcoma U-2 OS	ATCC	Cat# HTB-96
Human osteosarcoma U-2 OS (PKR knock-out exons 3-8)	this paper	N/A
NIH 3T3 cells	ATCC	Cat# CRL-1658
NIH 3T3 cells (PKR knock-out exons 3-8)	this paper	N/A
RS7 parental ESC strain	this paper	Czech Centre for Phenogenomics
Sf9 cells	ThermoFisher Scientific	Cat# 11496015
Experimental models: Organisms/strains		
DicerGNT mouse strain	this paper	N/A
DicerDQCH mouse strain	this paper	N/A
DicerX mouse strain	this paper	N/A
DicerSOM mouse strain	Taborska et al., 2019	N/A
Oligonucleotides		
GUCCAGUUUUCCCAGGAAUCCC UUGGAUGCUAAGAUGGGGAUUC CUGGAAAUACUGUUCUUG	this paper; RNA oligonucleotide	Sigma-Aldrich, pre-miR-145a
UAGCAGCACAUAAUGGUUUGUGG AUGUUGAAAAGGUGCAGGCCAUA CUGUGCUGCCUCA	this paper; RNA oligonucleotide	Sigma-Aldrich, pre-miR-15a
GUGAGGCUCAGUAUGGGGUGGG GGUGUCGUCGCCUGCCCGACUG ACCACCCACUCACCCUGGACUG ACUCUCAG	this paper; RNA oligonucleotide	Sigma-Aldrich, pre-miR-7068
AGAGGAGAGGGACAAUCAUAAA GGCCACUCGCAAGAGUGGCCU UUAUGAUUGUCCCUCUCCUCUUU	this paper; RNA oligonucleotide	Sigma-Aldrich, 30bp stem-loop ,
AGAGGAGAGGGACAAUAGAG GAGAGGGACAAUCAUAAAG GCCGCAAGGCCUUUAUGAUU GUCCCUCUCCUCUAUUGUC CCUCUCCUCUUU	this paper; RNA oligonucleotide	Sigma-Aldrich, 42bp stem-loop
GTACCCAAATGGATAGAA	this paper; sgRNA target site in intron 2	mDcr_i2a
GTTGGGATGGAGGTTGTT	this paper; sgRNA target site in intron 2	mDcr_i2b
GAGATGAGTCCTATAAAGGGG	this paper; sgRNA target site in intron 2, No.1	mDcr_i2-1
CCCCTCTGTCTCCTAAACTGC	this paper; sgRNA targeting intron 2, No.2:	mDcr_i2-2
ACGGGAAGAAGAAATGGCTGG	this paper; sgRNA target site in intron 2, No.3	mDcr_i2-3
ACTACGCTAGGTGTAAACAG	this paper; sgRNA target site in intron 6	mDcr_i6a
TGCAGTCCCCGGACGTTAAAT	this paper; sgRNA target site in intron 6	mDcr_i6b
GCCATCTAGATATACAGGAGG	his paper, sgRNA target site in intron 8, No.1	mDcr_i8-1

(*Continued on next page*)

CellPress
OPEN ACCESS

Continued

REAGENT or RESOURCE	SOURCE	IDENTIFIER
CCTTACCCTTCCACACGTCAC	this paper; sgRNA target site in intron 8, No.2	mDcr_i8-2
CCTTCTTTAACACTTGGCTTC	this paper; sgRNA target site in intron 1	Pkr_i1a
CCTGTGGTGGGTTGGAAACAC	this paper; sgRNA target site in intron 1	Pkr_i1b
GTGGAGTTGGTGGCCACGGGG	this paper; sgRNA target site in intron 5	Pkr_i5a
CCTGTGTACCAACAATGATCC	this paper; sgRNA target site in intron 5	Pkr_i5b
GCCTTGTTTTGACCATAAATGCCG	this paper; PKR genotyping primer	Pkr.fwd
GTGACAACGCTAGAGGATGTTCCG	this paper; PKR genotyping primer	Pkr.rev
GATATAACCAGCTCAAGTGTTTGC	this paper; Dicer genotyping primer (1st round of nested PCR)	mDcr_i1_Fwd
GAGCAAAAAGTTCATCAGGAACC	Dicer genotyping primer (1st round of nested PCR)	mDcr_i7_Rev
GCCTGGTTGGGTATAGACTGCTTG	this paper; Dicer genotyping primer (2nd round of nested PCR)	mDcr_i1_Fwd2
CAGAGGGCTAGAGCATACAAACAC	this paper; Dicer genotyping primer (2nd round of nested PCR)	mDcr_i7_Rev2
CAAGCCCGCCTCTTCTGATT	this paper; DicerGNT genotyping primer	Dicer_26720F
ATGGCACGAATGACTGAACC	this paper; DicerGNT genotyping primer	Dicer_28976R
CAGGTCTCATCTGCCAAGGT	this paper; DicerDQCH genotyping primer	DQCH_30860F
TGGAAGCAAGGCTTAGGAAA	this paper; DicerDQCH genotyping primer	DQCH_33000R
cacgacatcgactacaaggacg acgacgacaagTGAAGCGGC CGCTTCCCT	this paper; cloning oligo	2xFLAG_F
gtccttgtagtcaccgtcgtggtcc ttgtagtcGCTATTGGGAACCTG AGGTTGATTAGC	this paper; cloning oligo	2xFLAG_Rev
GGGCTTTATGAAAGACTGC	this paper; cloning oligo	dHEL1_F
TTGCAAAGCAGGGCTTTT	this paper; cloning oligo	dDExD_R & dHEL1
AACACGGCCATTGGACAC	this paper; cloning oligo	dDExD_F & dHEL2_F
TAAGACAACTGCTGTGTATCTTC	this paper; cloning oligo	dHEL2_R
GAAGATGTGGAAATCAAGCCTCGCG	this paper; cloning oligo	dmHEL1_F
GGACACCATGACCTCTGTGGGCTTG	this paper; cloning oligo	dmHEL1_R
GTGGAAGCAGCTACCGACCA TAACACAATTGTGTGCTTGAA CACTGGCTCAGGGAAGACGT TCATCGCGGTCCTGCTCACC AAAGAGCTGGCCCAGCAGA TCAGGG	this paper; cloning oligo	VTLQC_F
CAAGTTCTGACGGCTGACAC TTGTTGAGCAACCTGGTTTGC AGAGTTGACGAGGAACAC GGTCCTTTTTGCATGCGGG TTGAGGTCGCCCCTGATCT GCTGGGCCAGCTCTTTGGT	this paper; cloning oligo	VTLQC_R
AAGGACCATAACACAATTGT GTGCTTGAACACTGGCTCAG GGAAGACGTTCATCGCGGTC AAGCTCACCAAAGAGCTGGC CAAGCAGATCAGGGGCGACC TCAACC	this paper; cloning oligo	LKKKK_F

(*Continued on next page*)

Continued

REAGENT or RESOURCE	SOURCE	IDENTIFIER
CTTAGTTCTGACGGCTGACA CTTGTTGAGCAACCTGGTTT GCAGAGTTGACGAGGAACAC GGTCCTTTTTGCATGCGGGT TGAGGTCGCCCCTGATCTG CTTGG	this paper; cloning oligo	LKKKK_R
CCGTTCATTTCCCAGCCTGT	this paper; genotyping	Deletion confirmation - forward primer
AAAACAGCCCAATTCCTTGCC	this paper; genotyping	Deletion confirmation - reverse primer
ATCTACGGATCCACCATGGTATGGA GCCATCCTCAATTTGAAAAGGG TGGCGGGTCCGGCGGTGGGTC TGGCGGTAGCGCTTGGTCCCA CCCCCAGTTCG	this paper; cloning oligo	Twin-HA-TEV_Fwd
GTAGATGTCGACCAGGCCCTGAA AATACAGGTTTTCGGTACCAGCGT AATCTGGAACATCGTATGGGTAGT CACCCTTCTCGAACTGGGGGTGG GACCAA	this paper; cloning oligo	Twin-HA-TEV_Rev
TCTACAGCGGCCGCGGCGAGA ATCTCTACTTCCAAGGCGCTAG CGACTATAAGGACCACGACGG AGACTA	this paper; cloning oligo	C_TEV-FLAG-His_Fwd
GTAGATAAGCTTAGTGATGGTG ATGGTGATGGTGGTGGGACCC ATCATGATCCTTGTAGTCTCCG TCGTGGTCCTT	this paper; cloning oligo	C_TEV-FLAG-His_Rev
ATGTCGACGCAGGCCTGCAGC TCATGACCCC	this paper; cloning oligo	mDicer_SalI_Fwd
CAGTCGACAGCCGTGATACAG AAGTATACAC	this paper; cloning oligo	mDicerO_SalI_Fwd
ATGCGGCCGCTGTTAGGAACCT GAGGCTGGTTAGC	this paper; cloning oligo	mDicer-NotI_Rev
GCTGACAAGAGCATAGCGGAC TGTGTTGCTGCACTGCTGGGC TGCTACTTAACCAGC	this paper; cloning oligo	mDicer E1560A Forward
GCTGGTTAAGTAGCAGCCCAG CAGTGCAGCAACACAGTCCGC TATGCTCTTGTCAGC	this paper; cloning oligo	mDicer E1560A Reverse
CAAGGCCATGGGGGACATTT TTGCATCTCTTGCTGGTGCC ATTTATAT	this paper; cloning oligo	mDicer E1807A Forward
ATATAAATGGCACCAGCAAG AGATGCAAAAATGTCCCCCATGGCCTTG	this paper; cloning oligo	mDicer E1807A Reverse
**Recombinant DNA**		
CRISPR-Cas9 plasmid	Taborska et al., 2019	N/A
DicerSOM expression plasmid	Addgene	Cat# 120540
DicerX expression plasmid	Addgene	Cat# 120541
Firefly luciferase reporter – FL plasmid	Addgene	Cat# 120522
Hairpin-expressing plasmid CAG-EGFP-Elavl2IR	Addgene	Cat# 120518
Hairpin-expressing plasmid CAG-EGFP-Lin28IR	Addgene	Cat# 120517
Hairpin-expressing plasmid CAG-EGFP-MosIR	Addgene	Cat# 120516

(*Continued on next page*)

Continued

REAGENT or RESOURCE	SOURCE	IDENTIFIER
Hairpin-expressing plasmid CAG-EGFP-MosMos	Addgene	Cat# 120515
Hairpin-expressing plasmid CAG-EGFP-RlucIR	this paper	N/A
MosIR plasmid	Addgene	Cat# 120516
pCIneo 5'-DICER1(dHEL1)-2xFLAG	this paper	N/A
pCIneo 5'-DICER1(dHEL2)-2xFLAG	this paper	N/A
pCIneo 5'-DICER1(dDExD)-2xFLAG	this paper	N/A
pEF1-MH.BI-mDcr^{OO}	Addgene	Cat# 120541
pEF1-MH.BI-mDcr^{SOM}	Addgene	Cat# 120540
pFastBACT1 5'-TwinStrep-HA-TEV-Dicer^{O}-TEV-2xFLAG-8xHis	this paper	N/A
pFastBACT1 5'-TwinStrep-HA-TEV-Dicer-TEV-2xFLAG-8xHis	this paper	N/A
pFastBACT1 TwinStrep-HA-TEV-Dicer(E1560A, E1807A)-TEV-2xFLAG-8xHis	this paper	N/A
pFastBACT1 TwinStrep-HA-TEV-Dicer^{O}(E1560A, E1807A)-TEV-2xFLAG-8xHis	this paper	N/A
pFastBACT1 plasmid	Invitrogen	N/A
puromycin selection plasmids	Taborska et al., 2019	N/A
Renilla luciferase reporter - RL-Lin28 plasmid	Addgene	Cat# 120520
**Software and algorithms**		
Alphafold	Jumper et al., 2021; Varadi et al., 2022	https://alphafold.ebi.ac.uk/
Coot 0.9.6.2	Emsley et al., 2010	https://www2.mrc-lmb.cam.ac.uk/personal/pemsley/coot/
crYOLO 1.7.6	Wagner et al., 2019	https://cryolo.readthedocs.io/en/stable/
cryoSPARC	Punjani et al., 2017	https://cryosparc.com/
cutadapt version 1.8.3	Martin, 2011	N/A
DESeq2	Love et al., 2014	N/A
EMAN2	Tang et al., 2007	https://blake.bcm.edu/emanwiki/EMAN2
fastx-toolkit version 0.0.14	http://hannonlab.cshl.edu/fastx_toolkit	N/A
featureCounts v.2.0.0	Liao et al., 2014	N/A
GCTF	Zhang, 2016	https://www2.mrc-lmb.cam.ac.uk/research/locally-developed-software/zhang-software/
GraphPad Prism 9.1.0	GraphPad Software	https://www.graphpad.com/scientific-software/prism/
ISOLDE 1.1.0	Croll, 2018	https://isolde.cimr.cam.ac.uk/static/isolde/doc/isolde.html
Molprobity	Davis et al., 2004, 2007; Williams et al., 2018	http://molprobity.biochem.duke.edu/?fbclid=IwAR23Tilo_fFJl0iW0JjnMBtSo2JRdRKoxNt2tsD4m7hPt3FzRzvJG08IDpU
MotionCor2	Zheng et al., 2017	https://emcore.ucsf.edu/ucsf-software
Multi Gauge v3.2	Fujifilm, Tokyo, Japan	N/A
pcaExplorer	Marini and Binder, 2019	N/A
PHENIX 1.19.2-4158-000	Liebschner et al., 2019	https://phenix-online.org/

(Continued on next page)

Continued		
REAGENT or RESOURCE	SOURCE	IDENTIFIER
Relion 3.1	Scheres, 2012, 2016	https://www3.mrc-lmb.cam.ac.uk/relion/index.php/Main_Page
RNAComposer	Antczak et al., 2016; Popenda et al., 2012	https://rnacomposer.cs.put.poznan.pl/
SerialEM	Mastronarde, 2005	N/A
STAR 2.7.3a	Dobin et al., 2013	N/A
TOPAZ	Bepler et al., 2019	http://cb.csail.mit.edu/cb/topaz/
UCSC tools	Kent et al., 2010	N/A
UCSF Chimera 1.16	Pettersen et al., 2004	https://www.rbvi.ucsf.edu/chimera/
UCSF ChimeraX 1.3	Pettersen et al., 2021	https://www.rbvi.ucsf.edu/chimerax
Validation report at wwPDB (PDB Validation tool)	Berman et al., 2003	https://validate-rcsb-1.wwpdb.org/
original codes	this paper	DOI: https://doi.org/10.5281/zenodo.7154385
Other		
Lacey carbon M300	SPI supplies	Cat# 3830C-MB
Mindray 5300 Vet	Mindray	N/A
SkyScan 1272 high-resolution microCT	Bruker	N/A
UltraAuFoil M300 (R1.2/1.3)	Quantifoil	Cat# Q350AR13A

RESOURCE AVAILABILITY

Lead contact

Further information and requests for resources and reagents should be directed to and will be fulfilled by the lead contact Petr Svoboda (svobodap@img.cas.cz).

Materials availability

Animals and plasmids are available upon request from the lead contact.

Data and code availability

- The accession numbers of EM maps and their corresponding coordinates reported in this paper can be found in Table S4 and the key resources table. RNA sequencing data were deposited to Gene Expression Omnibus (GEO) with the following accession numbers GSE196310, their overview is provided in Table S5.
- All original codes have been deposited at Zenodo: https://doi.org/10.5281/zenodo.7154385 and are also available from GitHub: https://github.com/fhorvat/2022.DicerX_invivo.
- Any additional information required to reanalyze the data reported in this paper is available from the lead contact upon request.

EXPERIMENTAL MODEL AND SUBJECT DETAILS

Animals

Mus musculus genetically modified strains Dicer^{GNT}, Dicer^{DQCH}, Dicer$^{\Delta HEL1}$, and Dicer^{SOM}

Animal experiments concerning *Dicer^{GNT}* and *Dicer^{DQCH}* model were carried out in accordance with the Italian law under a license from the Italian Ministry of Health. Animal experiments concerning *Dicer$^{\Delta HEL1}$* and *Dicer^{SOM}* models were carried out in accordance with the Czech law and were approved by the Institutional Animal Use and Care Committee (approval no. 34-2014).

Dicer ^{SOM} and Dicer $^{\Delta HEL1}$ mutant mice

Production of *Dicer$^{\Delta HEL1}$* model was analogous to production of *Dicer^{SOM}* described previously (Taborska et al., 2019). We first produced ESCs with the *Dicer$^{\Delta HEL1}$* allele and then used those for producing chimeric mice and establishing *Dicer*$^{\Delta HEL1}$ line upon germline transmission of the *Dicer*$^{\Delta HEL1}$ allele. *Dicer*$^{\Delta HEL1}$ allele in ESCs (Nagy et al., 1993) was generated using CRISPR-Cas9 (Ran et al., 2013) mediated modification of the endogenous *Dicer* locus. Pairs of sgRNAs were designed to cleave *Dicer* genomic sequence in intron 2 (sequence of DNA targets: mDcr_i2a 5′-GTACCCAAATGGATAGAA-3′, mDcr_i2b 5′-GTTGGGATGGAGGTTGTT-3′) and intron 6 (sequence of DNA targets: mDcr_i6a 5′-ACTACGCTAGGTGTAAACAG-3′, mDcr_i6b 5′-TGCAGTCCCCGGACGTTAAAT-3′). A template for homologous recombination was designed to contain an HA-tag at the N-terminus of *Dicer* coding sequence fused

to exon 7 of Dicer and ~ 1.5 kb overhangs on both ends (Figure S1A). Final genomic sequence of *Dicer*$^{\Delta HEL1}$ mice is provided in Document S1.

To produce *Dicer*$^{\Delta HEL1}$ mouse strain, we first produced mouse chimeras by ESC microinjection into eight-cell – stage embryos (Poueymirou et al., 2007); host embryos were isolated from C57Bl/6NCrl mice (Figure S1). We used two ESC lines with C57Bl/6NCrl background (commonly used JM8A3.N1 and homemade RS7) and one in 129 strain (R1). For the first three rounds of chimera production, we used homozygous and heterozygous mutant ESCs and obtained mice with varying degree mosaicism, but we failed to obtain transmission of the mutant allele into the next generation. During the fourth round, a heterozygous ESC clone D11 derived from R1 ESC line yielded a male with > 80% chimeric fur. Breeding of this male with ICR females finally lead to germline transmission of *Dicer*$^{\Delta HEL1}$ allele into the next generation and establishment of the *Dicer*$^{\Delta HEL1}$ mouse line. Sequences of the engineered *Dicer* locus in the mouse genome are provided in the File S1. Phenotype analysis was performed with N3 animals, small RNA seq was done with N7 and N8 embryos (all breedings to ICR background).

Dicer^{GNT} mutant mice

The *Dicer*^{GNT} allele was generated by replacing wild-type exon 3 with a mutant exon in which Lys60 was mutated to encode asparagine. The *Dicer* locus was targeted with a vector containing homology arms and a *loxP*-flanked neomycin cassette 5′ of exon 3 that contained the Lys60Asn mutation. Southern blotting of genomic SacI-digested DNA from individual ESC-derived clones with a 3′ probe was used to identify homologous recombinants, where the *Dicer*$^{GNT\text{-}Neo}$ allele displaying a 5.9-kb DNA fragment could be distinguished from the wild-type allele of 7.1-kb fragment size. Cre-mediated recombination resulted in the excision of the *loxP*-flanked neomycin cassette and the generation of the *Dicer*^{GNT} allele. Mice analyzed in this study were on a C57Bl/6 genetic background.

Dicer^{DQCH} mutant mice

The *Dicer*$^{FH\text{-}DQCH}$ allele was generated by retargeting the *Dicer*^{Neo} allele, which contains a Flag-HA-HA sequence 5′ of exon 2 and a *loxP*-flanked neomycin cassette within intron 2 (Comazzetto et al., 2014). This was achieved with a vector comprised of homology arms, an FRT-flanked hygromycin cassette and exon 5 in which the Glu166 codon was mutated to encode glutamine. Southern blotting of genomic SacI-digested DNA from individual ESC-derived clones with a 3′ probe was used to identify homologous recombinants with the *Dicer*$^{FH\text{-}DQCH\text{-}Neo\text{-}Hyg}$ allele displaying a 7.8-kb DNA fragment. Flp-mediated recombination removed the FRT-flanked hygromycin cassette and generated the *Dicer*$^{FH\text{-}DQCH\text{-}Neo}$ allele that was identified with the 3′ probe as a 5.9-kb SacI DNA fragment. Cre-mediated recombination led to the excision of the *loxP*-flanked neomycin cassette and the generation of the *Dicer*$^{FH\text{-}DQCH}$ allele.

The targeting for both alleles was performed in A9 ES cells. Targeted ES cells were injected into C57BL/6 eight-cell-stage embryos. Targeted mice were crossed to deleter Cre mice (Schwenk et al., 1995) or FLP- expressing transgenic mice (Farley et al., 2000) to remove antibiotic resistance cassettes. The mice analyzed in this study were on a C57Bl/6 genetic background.

Cell culture and transfection

Mouse ESCs were cultured in 2i-LIF media: KnockOut-DMEM (ThermoFisher) supplemented with 15% fetal calf serum (Sigma), 1x L-Glutamine (Sigma), 1x non-essential amino acids (ThermoFisher), 50 μM β-Mercaptoethanol (ThermoFisher), 1000 U/mL LIF (Isokine), 1 μM PD0325901, 3 μM CHIR99021 (Selleck Chemicals), penicillin (100 U/mL), and streptomycin (100 μg/mL). All plastic was coated with 1% gelatin (Sigma) in PBS.

NIH 3T3 fibroblasts were cultured in DMEM (Sigma) supplemented with 10% fetal calf serum, penicillin (100 U/mL), and streptomycin (100 μg/mL).

METHOD DETAILS

Phenotype analyses

Genotyping

Tail biopsies were processed by PCR genotyping kit (Top-Bio) according to the manufacturer's protocol. 1 μl aliquots were used for genotyping PCR using 0.5 U/reaction of DNA polymerase (highQu). Genotyping primers are provided in the key resources table.

Embryo harvest

Mice were mated overnight, and the presence of a vaginal plug indicated embryonic day (E) 0.5. The embryos were washed in PBS and fixed in 4% PFA.

Proliferation assay - EdU staining and apoptosis TUNEL assay

Pregnant mice were injected with 60 μl of 10mM EdU 1.5 hour before embryo harvest at E10.5 and E14.5. The incorporation of EdU was visualized by Click-it EdU Imaging Kit (Invitrogen) in E10.5 whole mount samples and on 7μm paraffin sections from E14.5 embryos. Apoptosis was visualized in whole mount E10.5 embryos by TUNEL method using *In Situ* Cell Death Detection Kit, TMR red (Sigma-Aldrich).

MicroCT

E18.5 embryos were fixed for 1 week in 4% PFA and stained with Lugol's Iodine solution for 2 weeks. Stock solution (10g KI and 5g I2 in 100ml H2O) was diluted to 25% working solution in water. Stained specimens were embedded in 2.5% low gelling temperature agarose. Scan was performed on SkyScan 1272 high-resolution microCT (Bruker, Belgium), with resolution set to 4 μm.

Hematopoiesis panel

20 ul of blood from each E18.5 embryo was collected in tube containing anticoagulant EDTA and diluted with 175uL of V-53D Diluent (Mindray, 105-000146-00). The samples were measured in mode Complete blood count with Differentials (CBC + DIFF) on analyzer Mindray 5300 Vet. One-way Anova with Tukey posttest was used for statistical analysis.

RNAi activity in cultured cells assay

Effects of different Dicer isoforms on RNAi-mediated repression in Pkr$^{-/-}$ U-2 OS or 3T3 cells were monitored as described previously (Demeter et al., 2019). Briefly, cells were co-transfected with a plasmid expressing a Dicer variant (or LacZ as a negative control), dsRNA (Lin28IR, RlucIR, or MosIR), a targeted *Renilla* luciferase reporter with complementary sequences to dsRNA from Lin28IR and RlucIR, and a non-targeted firefly luciferase.

For transfection, cells were plated on 24-well plates, grown to 80% density and transfected using Lipofectamine 3000 (Thermo Fisher) according to the manufacturer's protocol. The total amount of transfected DNA was kept constant (1 μg/well).

Specific repression of the targeted *Renilla* luciferase was estimated as *Renilla* luciferase activity normalized to the non-targeted firefly luciferase activity, and non-specific effect of MosIR (expressing a non-targeting dsRNA). The value 1.0 corresponds to absence of RNAi, the value of LacZ negative control reflects repression mediated by endogenously-expressed Dicer.

Western blotting

Mouse tissues, U-2 OS cells transfected with Dicer variants or ES cells were homogenized mechanically in RIPA lysis buffer supplemented with 2x protease inhibitor cocktail set (Millipore) and loaded with SDS dye. Protein concentration was measured by Bradford assay (Bio-Rad) and 80 μg of total protein was used per lane. Proteins were separated on 5.5% polyacrylamide (PAA) gel and transferred on PVDF membrane (Millipore) using semi-dry blotting for 50 min, 35 V. The membrane was blocked in 5% skim milk in TBS-T, Dicer was detected using anti-HA 3F10 monoclonal primary antibody (High Affinity rat IgG1, Roche #11867431001; dilution 1:500), anti-HA rabbit primary antibody (Cell Signaling, #3724, dilution 1:1,000) or anti-Flag (M2 mouse monoclonal antibody, Sigma #F3165, dilution 1:10,000) and incubated overnight at 4°C. Secondary anti-Rat antibody (Goat anti-Rat IgG, HRP conjugate, ThermoFisher #31470, dilution 1:50,000), HRP-conjugated anti-Mouse Igg binding protein (Santa-Cruz #sc-525409, dilution 1:50,000) or anti-Rabbit-HRP antibody (Santa-Cruz #sc-2357, dilution 1:50,000) was incubated 1 h at room temperature. For TUBA4A and TARBP2 detection, samples were run on 10% PAA gel and incubated overnight at 4 °C with anti-Tubulin (Sigma, #T6074, dilution 1:10,000) or anti-TARBP2 (ThermoFisher #LF-MA0209, dilution 1:1,000) mouse primary antibodies. HRP-conjugated anti-mouse IgG binding protein (Santa-Cruz, #sc-525409, dilution 1:50,000) was used for detection. Signal was developed on films (X-ray film Blue, Cole-Parmer #21700-03) using SuperSignal West Femto Chemiluminescent Substrate (Thermo Scientific).

Immunoprecipitation

NIH 3T3 cells transfected with plasmids expressing HA-tagged Dicer$^{\Delta HEL1}$ or Dicer^{SOM} variants were lysed in IP Lysis Buffer (10 mM phosphate buffer, pH 7.2, 120 mM NaCl, 1 mM EDTA, 0.5% v/v NP-40, 10% v/v glycerol). Insoluble material was pelleted by centrifugation. Cleared supernatants were diluted 4-times with IP Dilution Buffer (10 mM phosphate buffer, pH 7.2, 100 mM NaCl, 1 mM EDTA, 0.1% v/v NP-40) and incubated with anti-HA magnetic beads (anti-HA mAb, clone #2-2.2.14, ThermoFisher #88836) for 2 h on a rotator. Beads were washed 4-times with IP Dilution Buffer, finally re-suspended in 60 μl water and processed for western blotting. All buffers were supplemented with 1x Protease Inhibitor Cocktail Set (Millipore) and the whole procedure was performed at 4 °C.

RNA sequencing

ESC small RNA-seq

Cells were plated on 6-well plates and grown to 80 % density. Cells were transfected with 2 μg/well of pCAG-EGFP-MosIR plasmid and cultured for 48 hours. Cells were washed with PBS, homogenized in Qiazol lysis reagent (Qiagen) and total RNA was isolated by Qiazol-chloroform extraction and ethanol precipitation method (Toni et al., 2018). RNA quality was verified by Agilent 2100 Bioanalyzer. Small RNA libraries were constructed using NEBNext Multiplex Small RNA Library Prep Set for Illumina (New England Biolabs) according to the manufacturer's protocol. Small RNA libraries were size selected on 6% PAAGE gel, a band of 140 - 150 bp was cut from the gel and RNA was extracted using Monarch® Genomic DNA Purification Kit. Quality of the libraries was assessed by Agilent 2100 bioanalyzer. Libraries were sequenced on the Illumina HiSeq2000 platform at the Genomics Core Facility at EMBL.

E15.5 small RNA-seq

E15.5 embryos were removed from the uterus and washed in PBS. The yolk sac was taken for genotyping and embryos were transferred into RNAlater (Thermo Fisher Scientific). Embryos were homogenized in Qiazol lysis reagent (Qiagen) and total RNA was isolated by Qiazol-chloroform extraction and ethanol precipitation method (Toni et al., 2018). Small RNA libraries were constructed using Nextflex Small RNA-seq kit v3 for Illumina (Perkin Elmer) according to the manufacturer's protocol; 3′ adapter ligation was performed overnight at 20 °C, 15 cycles were used for PCR amplification and NextFlex beads were used for size selection. Final libraries were sequenced by 75-nucleotide single-end reading using the Illumina NextSeq500/550 platform at the core genomics facility of IMG.

Bioinformatic analyses

RNA-seq data (Table S5) were deposited in the Gene Expression Omnibus database under GEO: GSE196310.

Mapping of small RNA-seq data

Small RNA-seq reads were trimmed in two rounds using fastx-toolkit version 0.0.14 (http://hannonlab.cshl.edu/fastx_toolkit) and cutadapt version 1.8.3 (Martin, 2011). First, 4 random bases were trimmed from left side:

```
fastx_trimmer -f 5 -i {INP}.fastq -o {TMP}.fastq
```

Next, NEXTflex adapters were trimmed. Additionally, the N-nucleotides on ends of reads were trimmed and reads containing more than 10% of the N-nucleotides were discarded:

```
cutadapt –format="fastq" –front=”GTTCAGAGTTCTACAGTCCGACGATCNNNN” –adapter=”NNNNTGGAATTCTCGGGTGCCAAGG” –error-rate=0.075 –times=2 –overlap=14 –minimum-length=12 –max-n=0.1 –output=”$ {TRIMMED}.fastq" –trim-n –match-read-wildcards $ {TMP}.fastq
```

Trimmed reads were mapped to the mouse (mm10) genome using STAR aligner (Dobin et al., 2013) with following parameters:

```
STAR –readFilesIn $ {TRIMMED}.fastq.gz –runThreadN 4 –genomeDir $ {GENOME_INDEX} –genomeLoad LoadAndRemove –readFilesCommand unpigz -c –readStrand Unstranded –limitBAMsortRAM 20000000000 –outFileNamePrefix $ {FILENAME} –outReadsUnmapped Fastx –outSAMtype BAM SortedByCoordinate –outFilterMultimapNmax 99999 –outFilterMismatchNoverLmax 0.1 –outFilterMatchNminOverLread 0.66 –alignSJoverhangMin 999 –alignSJDBoverhangMin 999
```

miRNA expression analyses

Mapped reads were counted using program featureCounts (Liao et al., 2014). Only reads with lengths 19-25nt were selected from the small RNA-seq data:

```
featureCounts -a $ {ANNOTATION_FILE} -F $ {FILE} -minOverlap 15 -fracOverlap 0.00 -s 1 -M -O -fraction -T 8 $ {FILE}.bam
```

The GENCODE gene set (Frankish et al., 2019) was used for the annotation of long RNA-seq data. The miRBase 22.1 (Kozomara et al., 2019). set of miRNAs was used for the annotation of small RNA-seq data for main figures, mirGeneDB annotation of high-confidence miRNAs (Fromm et al., 2022) was used to make sure that results were not biased by annotated low-confidence miRNAs from the miRBase. Statistical significance and fold changes in gene expression were computed in R using the DESeq2 package (Love et al., 2014). Genes were considered to be significantly up- or down-regulated if their corresponding p-adjusted values were smaller than 0.05.

miRNA expression plots – normalization of data, miRNA and miRNA* sorting

First, the relative position of each mature miRNA (“5p” and “3p” for the miRNA-5p and miRNA-3p, respectively) provided by miRBase 22.1. annotation (Kozomara et al., 2019) was manually curated and completed. Second, the miRNA type of each mature miRNA (“miRNA” and ”miRNA*” for the guide strand and passenger strand miRNA, respectively) provided by miRBase annotation was completed in this way:

1) The mature miRNAs were assigned into the pair by their hairpin names. The DESeq2 baseMean values of E15.5 and GNT experiments were added to each mature miRNA.
2) The pairs of mature miRNAs with complete miRNA type annotation (both, “miRNA” and “miRNA*” types were present) were preserved.
3) If there is only one mature miRNA annotated in the hairpin, it is assigned as “single_miRNA”.
4) If the baseMean values of both miRNAs in the pair are lower than 0.25, it is assigned as “lowExp”.
5) For the remaining pairs of mature miRNAs, if the baseMean value of one miRNA is at least double to the second one, it is assigned as “miRNA” / “miRNA*” or “miRNA*” / “miRNA”, respectively. Otherwise it is assigned as “notClear”.
6) Finally, the newly determined miRNA types are compared to each other. If the mature miRNAs were determined as “miRNA” in one experiment and as “miRNA*” in the other, it is assigned as “cellSpecific”. In all the other cases, it there is any discrepancy among the determined miRNA type, it is assigned as “notCLear”.

The annotation of the mirtrons was taken from Ladewig et al. 2012.

mirGeneDB annotation of high-confidence miRNAs (Fromm et al., 2022) was used to make sure that results were not biased by annotated low-confidence miRNAs from the miRBase

The DESeq2 baseMean and fold changes were plotted and visualized by home-made R scripts.

The MA plots related to the dominant or passenger strand miRNAs contain only the corresponding miRNAs, all the miRNAs otherwise.

Small RNA clustering analysis

Small RNA read clusters (Figure S2F) were identified following the algorithm used in previous studies (Flemr et al., 2013; Demeter et al., 2019). Briefly:

1) Reads were weighted to fractional counts of 1/n where n represents the number of loci to which read maps

2) Reads were then collapsed into a unified set of regions and their fractional counts were summed
3) Clusters with less than 3 reads per million (RPM) were discarded
4) Clusters within 50 bp distance of each other were joined

Only clusters appearing in all replicates of the same genotype (intersect) were considered in the final set. Union of coordinates of overlapping clusters were used to merge the clusters between the samples. Clusters were then annotated, and if a cluster overlapped more than one functional category, the following classification hierarchy was used: miRNA > transposable elements > mRNA (protein coding genes) > misc. RNA (other RNA annotated in ENSEMBL or RepeatMasker; Smit et al., 2013–2015) > other (all remaining annotated or not annotated regions).

Cleavage fidelity analysis

Only miRNAs with DESeq2 baseMean values >= 100 were selected. The cleavage points' coordinates (CP) were extracted from their miRBase 22.1 annotation (Kozomara et al., 2019). The reads of the lengths 19-25nt were selected from each replicate library. The starting and ending position of all reads were summed up in the CP and its vicinity (+/-15nt) and assigned as 3′-CP of miRNA-5p and 5′-CP of miRNA-3p, respectively. Then, the canonical miRBase CPs were re-defined based on our wild-type data:

1) Position with maximal counts (median among replicates) is assigned as the new CP.
2) If the new CP is more than 7nt outside the canonical one, keep the canonical one.
3) If there are multiple CPs with the same max counts, keep the canonical one.
4) If there are no data / no reads, keep the canonical one.

The counts were extracted for each miRNA at the position of the newly defined CP with 5nt flanks on each side. The read counts were re-calculated into read densities. The final matrix was achieved as a subtraction between a mutant and its corresponding wild-type control. Top 50 miRNAs from *Dicer* mutants were selected based on the absolute value of the difference at the position of CP. Selected miRNAs were ordered by the change of *ESC* fidelity at the position of CP.

Partial processing analysis

All sequence reads were selected that overlapped the corresponding pre-miRNA locus in the sense direction. All coordinates (starting/ending position of the miRNA-5p/-3p) were extracted from the miRBase 22.1 annotation (Kozomara et al., 2019). The categories shown in the Figures 5D and S5D were defined by pre-miRNA boundaries and the two annotated Dicer cleavage points (deviation of the boundaries +/-2nt allowed). Each read was unambiguously assigned into the appropriate category. The percentage from the total number of overlapping reads was calculated.

Luciferase assay

Dual luciferase activity was measured according to Hampf and Gossen (Hampf and Gossen, 2006) with some modifications. Briefly, cells were washed with PBS and lyzed in PPTB lysis buffer (0.2% v/v Triton X-100 in 100 mM potassium phosphate buffer, pH 7.8). A 3-5 μl aliquots were used for measurement in 96-well plates using Modulus Microplate Multimode Reader (Turner Biosystems). First, firefly luciferase activity was measured by adding 50 μl substrate (20 mM Tricine, 1.07 mM $(MgCO_3)_4 \cdot Mg(OH)_2$, 2.67 mM $MgSO_4$, 0.1 mM EDTA, 33.3 mM DTT, 0.27 mM Coenzyme A, 0.53 mM ATP, 0.47 mM D-Luciferin, pH 7.8) and signal was integrated for 10 sec after a 2 sec delay. Signal was quenched by adding 50 μl *Renilla* substrate (25 mM Na_4PP_i, 10 mM Na-Acetate, 15 mM EDTA, 500 mM Na_2SO_4, 500 mM NaCl, 1.3 mM NaN_3, 4 μM Coelenterazine, pH to 5.0) and *Renilla* luciferase activity was measured for 10 sec after a 2 sec delay. Hairpin-expressing plasmids and luciferase reporters are described and deposited in Addgene. RlucIR plasmid expressing a hairpin structure targeted to *Renilla* luciferase coding region was prepared similarly to MosIR using common cloning techniques.

Recombinant plasmid preparation

pCIneo plasmid carrying human *DICER1* (GenBank: NM_1777438) was prepared by standard molecular cloning procedures. The C-terminal 2× FLAG tag and deletion (dHEL1, dHEL2 and dDExD) variants were prepared using Q5 Site-Directed Mutagenesis Kit (NEB) according to the manufacturer's instructions.

pFastBac plasmids carrying recombinant mouse full-length Dicer and short variant ($Dicer^{O}$) were prepared as follows. The N-terminal fragment containing TwinStrep and HA tags together with TEV protease cleavage site was PCR amplified and inserted into BamHI-SalI restriction sites in pFastBACT1 plasmid (Invitrogen). Subsequently, the C-terminal fragment containing 2xFLAG and 8xHis tags together with TEV protease cleavage site was PCR amplified and inserted into NotI-HindIII restriction sites.

Mouse Dicer and $Dicer^{O}$ omitting start and stop codons were PCR-amplified from pEF1-MH.BI-$mDcr^{SOM}$ (Addgene) and pEF1-MH.BI-$mDcr^{OO}$ (Addgene) plasmids, respectively, and inserted in-frame into SalI-NotI sites of the modified pFastBACT1 plasmid using common cloning techniques. C-terminal 2× FLAG tag and deletion variants were prepared using Q5 Site-Directed Mutagenesis Kit (NEB) according to the manufacturer's instructions (PCR primers: Twin-HA-TEV_Fwd, Twin-HA-TEV_Rev, 3C-FLAG-His_Fwd, 3C-FLAG-His_Rev, mDicer_SalI_Fwd, mDicerO_SalI_Fwd, mDicer-NotI_Rev).

The catalytically inactive variants of Dicer/$Dicer^{O}$ were prepared by mutating the key residues E1560 and E1807 of the RNAse III domains into alanine residues (Zhang et al., 2004) using Q5 Site-Directed Mutagenesis Kit (NEB) kit according to the manufacturer's

instructions (PCR primers: mDicer E1560A Forward, mDicer E1560A Reverse, mDicer E1807A Forward, mDicer E1807A reverse). List of all used oligonucleotides can be found in key resources table. All constructs were verified by sequencing.

Dicer variants with mutations in HEL1 domain (VTLQC, LKKKK, Y1688A, and V1755A/F1760A) and with swapped HEL1 domain to the one from *D. melanogaster* Dcr-2 were prepared using Gibson Assembly Cloning kit (NEB) according to the manufacturer's instructions.

Preparation of recombinant proteins

The coding sequence and the necessary regulatory sequences of mouse Dicer variants or TARBP2 were transposed into bacmid using *E. coli* strain DH10bac. The viral particles were obtained by transfection of the bacmids into the Sf9 cells using FuGENE Transfection Reagent (Eastport) and further amplification in Sf9 cells.

Dicer variants were expressed in 200 ml of Hi5 cells (infected at 1.2×10^6 cells/ml) with the corresponding P1 virus at multiplicity of infection >1. The cells were harvested 48 hours post infection, washed by 1x PBS, and stored at -80°C. Subsequent operations were carried out at + 4°C. Pellets were resuspended in ice-cold lysis buffer containing 50 mM Tris (pH 8.0), 300 mM NaCl, 0.4% Triton X-100, 10% (v/v) glycerol, 10 mM imidazole, 1 mM DTT, 2 mM $MgCl_2$, benzonase (250U), and protease inhibitors (0.66 μg/ml pepstatin, 5 μg/ml benzamidine, 4.75 μg/ml leupeptin, 2 μg/ml aprotinin) (Applichem). The resuspended cells were gently shaken for 10 min at 4°C. To aid the lysis, cells were briefly sonicated. The lysate was cleared by centrifugation at 21,000xg for 1 hr at 4°C. The supernatant was passed through a column containing 2.5 ml NiNTA-agarose (QIAGEN). The affinity matrix was washed 5-times with 15 ml of washing buffer (50 mM Tris (pH 8.0), 500 mM NaCl, 1 mM DTT, 2 mM $MgCl_2$, and 10 mM imidazole). The protein was eluted three times with 3.5 ml of elution buffer (50 mM Tris (pH 8.0), 500 mM NaCl, 1 mM DTT, 2 mM $MgCl_2$, and 300 mM imidazole). The fractions containing protein were pooled and concentrated to 1 ml using 100 kDa cut-off Vivaspin Turbo15 (Sartorius). The proteins were further purified on a size exclusion column (Superose 6 Increase 10/300 GL, GE Healthcare) equilibrated with a buffer containing 50 mM Tris (pH 8.0), 150 mM NaCl, 1 mM DTT, 2 mM $MgCl_2$. Fractions containing protein were pooled, concentrated, snap-frozen in liquid nitrogen, and stored at -80°C until further use.

Purification of the wild-type Dicer for structural studies included treatment by buffer containing 6 mM EDTA, prior to gel filtration. To preclude the RNA cleavage, the gel filtration buffer (and all buffers in subsequent procedures) contained 2 mM $CaCl_2$ instead of 2 mM $MgCl_2$.

TARBP2 was expressed in Sf9 cells (infected at 1.2×10^6 cells/ml) with the corresponding P1 virus at multiplicity of infection >1. The cells were harvested 48 hours post infection, washed by 1x PBS, and stored at -80°C. TARBP2 was purified as described for Dicer, except for size exclusion chromatography in which Superdex 75 Increase 10/300 GL (GE Healthcare) was used.

In vitro cleavage assay

Substrate preparation

In vitro synthesized RNA oligonucleotides were diluted to 250 nM with nuclease-free water and mixed with T4 Polynucleotide Kinase buffer. The RNA was refolded by heating the mixture at 95°C for 3 min and snap-cooled on ice for 5 min. After addition of RNase inhibitors (NEB), T4 polynucleotide kinase (NEB), and [γ-^{32}P]-ATP (HARTMANN ANALYTIC), the reaction was incubated at 37°C for 10 minutes. The 5′-radiolabelled RNA was purified on G-25 columns (GE Healthcare) and diluted to a final concentration of 50 nM. The radiolabelled RNA Decade Marker (ThermoFisher Scientific) was prepared according to the manual. The RNA and the marker were aliquoted and stored at -20°C.

Nuclease-activity assay

Time-course experiments were performed in 10 μl, containing 5 nM labelled RNA substrate, and 100 nM $Dicer^{SOM}$ and $Dicer^{\Delta HEL}$, respectively, in 30 mM Tris (pH 7.0), 30 mM NaCl, 1 mM DTT, and 2 mM $MgCl_2$ at 37°C. Increasing concentrations (12.5, 25, and 50) of $Dicer^{SOM}$ and $Dicer^{\Delta HEL1}$, respectively, were mixed with 5 nM labelled RNA substrate in 30 mM Tris (pH 7.0), 30 mM NaCl, 1 mM DTT, and 2 mM $MgCl_2$. After 60 min incubation at 37°C, the reactions were stopped with equal volume of 95% formamide, boiled for 5 min, and analyzed on a 20% polyacrylamide gel containing 8 M urea.

After electrophoresis, the gels were exposed for 6-18 hours onto a phosphor imaging screen (Fujifilm). The signal was detected using FLA 9000 phosphorimager (Fujifilm) and analyzed in Multi Gauge v3.2 software.

In vitro reconstitution of the Dicer–pre-miR-15a complex

To refold pre-miR-15a RNA, it was heated for 3 min at 95°C and snap-cooled on ice for 5 min. The complex was formed by mixing 1.5 nmol of pre-miR-15a and 0.5 nmol of catalytically inactive Dicer or $Dicer^{O}$ variant in 50 μl of 50 mM Tris (pH 8.0), 100 mM NaCl, 1 mM DTT, and 2 mM $MgCl_2$. After 30 min incubation on ice, the mixture was applied onto Superose 6 Increase 5/150 GL (Cytiva) column attached to an ÄKTA Purifier (Cytiva). Fractions containing the complex were collected and concentrated to 0.2 mg/ml. The complex of the wild-type Dicer with pre-miR-15a and TARBP2 was prepared by direct mixing of 150 pmol of pre-miR-15a, 50 pmol of Dicer and 55 pmol of TARBP2 in 50 μl of 50 mM Tris (pH 8.0), 100 mM NaCl, 1 mM DTT, and 2 mM $CaCl_2$. The mixture was incubated on ice for 30 min and applied on CryoEM grid. The purity and homogeneity of the protein was assessed by SDS-PAGE, while RNA was verified by denaturing gel electrophoresis (20% polyacrylamide gel containing 8 M urea) and visualized using SYBR Gold dye (ThermoFisher Scientific).

Cryo-EM specimen preparation and data acquisition

The purified Dicer or Dicer–pre-miR-15a complex were diluted to a concentration of about 1 μM in a buffer containing 50 mM Tris (pH 8.0), 100 mM NaCl, 1 mM DTT, and 2 mM $MgCl_2$. The Lacey carbon M300 grid (SPI supplies) was glow-discharged (15 sec, hydrogen-oxygen) immediately before preparing the cryo-EM specimen. In a Vitrobot Mark IV (ThermoFisher Scientific), 3.5 μl of the protein–RNA complex was applied on the grid from the plasma treated side. The grid was blotted for 5.0 sec, blot force -3, in 100% humidity at 4°C, and plunged in liquid ethane cooled by liquid nitrogen. For Dicer, UltraAuFoil M300 (R1.2/1.3) grid (Quantifoil) was glow-discharged (60 sec, argon-oxygen) and 3.5 μl of the protein was applied from the plasma treated side. The grid was blotted for 3.0 sec, blot force 0 in 100% humidity at 4°C. The data were collected using Titan Krios (ThermoFirsher Scientific) transmission electron microscope using SerialEM software (Mastronarde, 2005). The details about data acquisition, processing, structural refinement and validation are shown in Table S4.

Image processing of electron micrographs

The movies were first processed by MotionCor2 (Zheng et al., 2017) for generation of motion corrected, dose-weighted micrograph stacks. The CTF parameters were estimated using GCTF (Zhang, 2016). The micrographs were further manually curated to select for astigmatism lower than 800 Å and CTF fit parameter lower than 4.5 Å. For each dataset, a set of 30-50 randomly selected micrographs was used for manual particle picking using e2boxer.py tool from the EMAN2 (Tang et al., 2007) package. The manually picked particles were used for model generation using crYOLO (Wagner et al., 2019). The particles obtained from full dataset picking were imported into cryoSPARC (Punjani et al., 2017). Further analysis comprised the following steps, 2D classification, *ab-initio* modelling and 3D Refinement. The initial volume maps were used as a reference for re-analysis of the data using 3D Classification in Relion 3.1 (Scheres, 2012) and/or training of TOPAZ (Bepler et al., 2019) tool to improve the quality of particle picking procedure. The final 3D Refinement was performed in cryoSPARC. The detailed statistics are available in Table S4.

Cryo-EM model building and refinement

Initial PDB coordinates of the Dicer structure were taken from AlphaFold database (Jumper et al., 2021). Regions of low confidence prediction (pLDDT < 50) were excluded from the structure and the remaining blocks of the coordinates were fitted into the density map using UCSF Chimera's tool 'Fit in Map (Pettersen et al., 2004)'. The PDB coordinates and the density map were then imported into program Coot (Emsley et al., 2010) and the tool 'Real Space Refine Zone' was used to achieve optimal fit of the PDB coordinates within the map. Low resolution regions and regions where the map was lacking density were excluded from the structure. The dsRBD of Dicer was docked into map with rigid body approach and fit was optimized using Phenix 'rigid_body' strategy (Liebschner et al., 2019). The coordinates were validated using Coot's tools 'Ramachandran Plot', 'Rotamer Analysis', and 'Density Analysis'. The same procedure was applied to Dicer–pre-miR-15a complex. The initial coordinates of pre-miR-15a were obtained from a modeling server RNAComposer (Antczak et al., 2016; Popenda et al., 2012). The model was fitted and refined into the density map using ProSMART Self Restraints implemented in Coot software. The model of Dicer–pre-miR-15a was fitted and refined into Dicer–pre-miR-15a–TARBP2 pre-cleavage complex density map. The TARBP2 dsRBDs were fitted into the map according to the predicted structure obtained from AlphaFold. TARBP2 dsRBD1 and dsRBD2 were fitted into the non-sharpened map. The coordinates of the Dicer structure and the Dicer–RNA complexes in the pre-cleavage states were subjected to further structural refinement in the Dicer core region using Phenix software and ISOLDE (Croll, 2018). For the cleavage states of Dicer and Dicer^{O}, initial PDB coordinates of the Dicer/Dicer^{O} structure were predicted by AlphaFold software. After excluding low confidence prediction regions (pLDDT < 50), the structured were fitted into density maps obtained from CryoSparc as described above. Protein domains that were not resolved within the density map (residues 1–500) were excluded from the models. Modelled pre-miR-15a was manually fitted into the density map. MolProbity and PDB Validation tool was used to obtain the overall refinement and structural statistics.

Data visualization

Molecular graphics images were produced using the UCSF Chimera (Pettersen et al., 2004) and ChimeraX (Pettersen et al., 2021) package from the Resource for Biocomputing, Visualization, and Informatics at the University of California, San Francisco (supported by NIH P41 RR-01081) and/or Coot (Emsley et al., 2010).

QUANTIFICATION AND STATISTICAL ANALYSIS

In general, all of the experiments were performed with at least duplicate independent biological samples. The number of replicates was influenced by limited availability of the biological material. Differential expression analysis of miRNAs and mRNAs relied on statistics integrated into the DESeq2 tool. One-way Anova with Tukey posttest was used for statistical analysis of blood data. Two sided t-test was used for analysis of RNAi effects in transfection assays. Sample sizes or number of replicates are provided in the text and in figures. No statistical method was used to predetermine sample sizes.

For the quantification of the EMSA assays, the analyses were carried out using the Multi Gauge v3.2 software (Fujifilm). GraphPad Prism was used to plot the obtained values (Specific binding with Hill slope) and perform the statistical analysis. The bound fraction was determined as the disappearance of the signal corresponding to the unbound substrate (Lane 0). Each data point represents an average of at least two independent experiments. Error bars represent standard deviation (SD).

Molecular Cell, Volume *82*

Supplemental information

Structural and functional basis of mammalian microRNA biogenesis by Dicer

David Zapletal, Eliska Taborska, Josef Pasulka, Radek Malik, Karel Kubicek, Martina Zanova, Christian Much, Marek Sebesta, Valeria Buccheri, Filip Horvat, Irena Jenickova, Michaela Prochazkova, Jan Prochazka, Matyas Pinkas, Jiri Novacek, Diego F. Joseph, Radislav Sedlacek, Carrie Bernecky, Dónal O'Carroll, Richard Stefl, and Petr Svoboda

Supplemental information

Supplemental information includes the following material:

Figures

Figure S1 - Production and validation of Dicer mouse mutants.

Figure S2 - Phenotype of $Dicer^{\Delta HEL1/\Delta HEL1}$ mouse mutants.

Figure S3 - miRNome dysregulation in $Dicer^{\Delta HEL1/\Delta HEL1}$ mouse mutants

Figure S4 - Purification and cryo-EM analyses of apo-Dicer.

Figure S5 - Purification and cryo-EM analyses of Dicer–pre-miR-15a complex.

Figure S6 - Reconstitution and cryo-EM analyses of Dicer^{O}–pre-miR-15a complex.

Figure S7 - Reconstitution and cryo-EM analysis of Dicer–pre-miR-15a–TARBP2 complex.

Tables

Table S2 - Expression of host genes of most upregulated mirtrons in ESCs

Table S3 - Asymmetric cleavage of miRNAs

Table S4 - Cryo-EM data collection and refinement statistics

Table S5 - RNA-seq libraries

Table S1 - miRNA expression in mutants is provided in a non-PDF format separately

Supplementary Figures

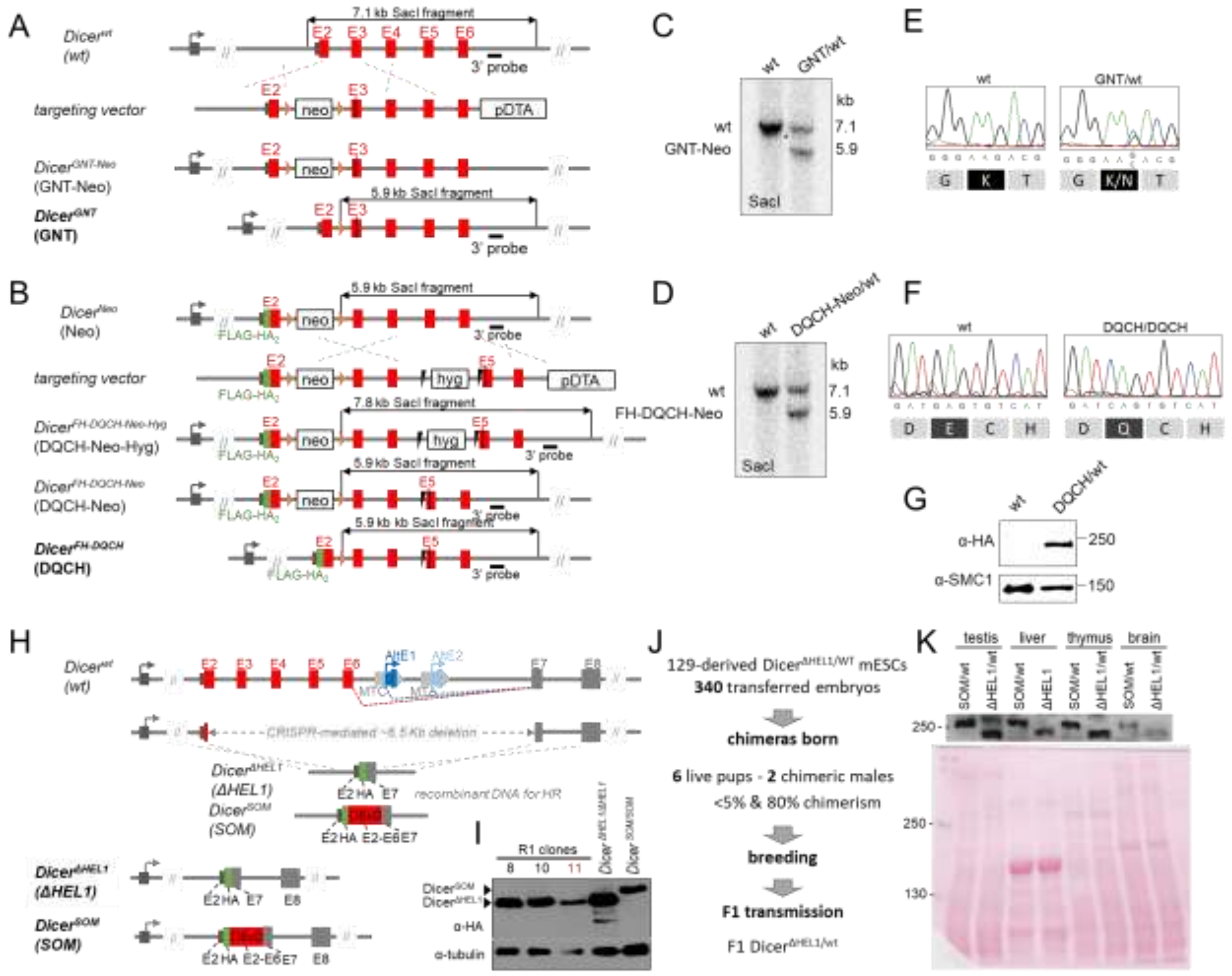

Figure S1 Production and validation of Dicer mouse mutants. Related to Figure 1A. (**A, B**) Schematic depiction of introduction of the GNT and the DQCH mutation into endogenous *Dicer* gene, respectively. (**C**) Detection of the GNT and (**D**) DQCH alleles by Southern blotting. (**E, F**) Validation of the mutated alleles by Sanger sequencing. (**G**) Western blot demonstrating expression of the mutated $Dicer^{DQCH}$ protein. (**H**) Schematic depiction of engineering of $Dicer^{\Delta HEL1}$ (ΔHEL1) and $Dicer^{SOM}$ (SOM) in the genomic sequence encoding HEL1 of the endogenous *Dicer* gene. Briefly, a fragment from exon 2 to exon 7 was removed using CRISPR /Cas9 and recombined with a $Dicer^{\Delta HEL1}$ recombination construct carrying exon 2 (5' UTR and start codon), HA-tag, and exon 7 coding sequence. $Dicer^{SOM}$, which was produced using the same strategy, was described previously[1]. Both alleles were validated by sequencing. (**I**) Western blotting of selected positive clones using anti-HA antibody, tubulin was used as a loading control. The heterozygous line 11 gave rise to $Dicer^{\Delta HEL1}$ mice. (**J**) Outline of the $Dicer^{\Delta HEL1}$ mouse strain production process. (**K**) Western blot analysis of $Dicer^{\Delta HEL1}$ expression in different tissues of a heterozygote $Dicer^{\Delta HEL1/wt}$ mouse. Tissues from $Dicer^{SOM/wt}$ mouse were used for comparison. 80 μg of total protein lysate were loaded per lane. Ponceau staining of the membrane shown below provides control for equal loading.

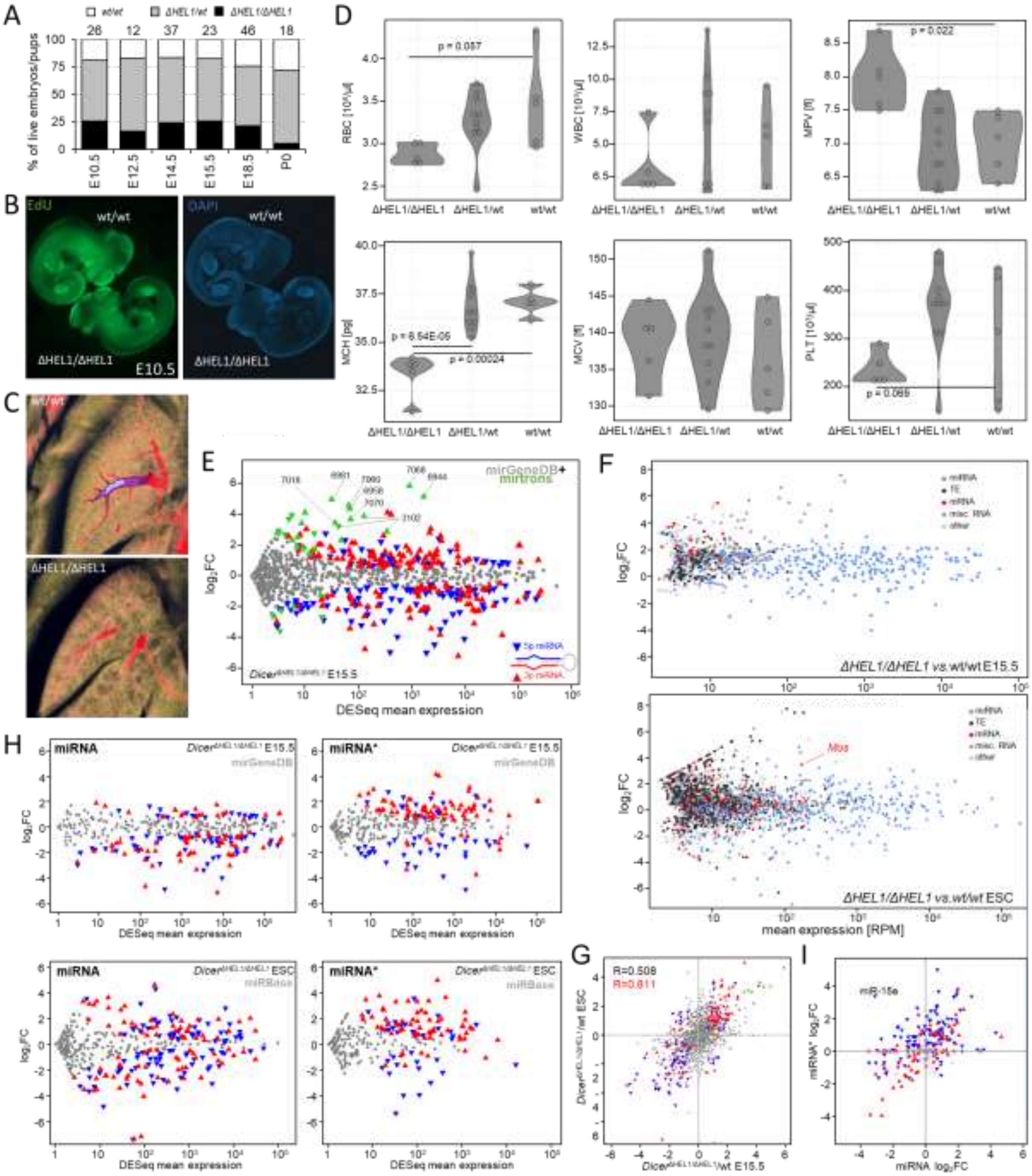

Figure S2 **Phenotype of *Dicer*$^{\Delta HEL1/\Delta HEL1}$ mouse mutants.** Related to Figure 1 and 2 (**A**) Genotype segregation in embryos from *Dicer*$^{\Delta HEL1/wt}$ parents. *Dicer*$^{\Delta HEL1/\Delta HEL1}$ embryos survive until birth but die soon after. (**B**) A combined EdU and DAPI staining of E10.5 embryos shows no apparent difference in general proliferation pattern suggesting more specific mechanism beyond the growth retardation phenotype. The experiment was performed twice with the same result. (**C**) MicroCT scans of lungs from E18.5 embryos. False coloring reveals reduced airway development and branching (red and purple colors) in *Dicer*$^{\Delta HEL1/\Delta HEL1}$ embryos. For each genotype a section with the highest signal was selected in corresponding anatomical positions. Scale bar = 5mm. (**D**) Defects in hematopoiesis found in *Dicer*$^{\Delta HEL1/\Delta HEL1}$ mice. Shown are: red blood cell count per μl (RBC, reduced by 17%), mean red blood cell volume (MCV), mean hemoglobin per cell (MCH, reduced by 10%), platelet count per μl (PLT), mean platelet volume (MPV), and white blood cell count (WBC) per μl. (**E**) Differential expression of miRNAs is not an artifact of using miRBase annotationa. MA plot shows differentially-expressed high-confidence annotated murine miRNAs from mirGeneDB database [2], which reveals highly similar miRNA dysregulation as shown in Fig. 2H except of mirtrons, which are not included in the mirGeneDB and had to be analyzed separately using mirtron annotation by Ladewig et al. [3]. Significantly

dysregulated 5p and 3p miRNAs (DESeq p-value 0.05) are shown as oriented blue ▼ and red ▲ triangles, respectively. Mirtrons are represented by green triangles whose orientation is the same as that of significantly dysregulated 5p and 3p miRNAs. Three embryos with the wild type and five with the mutant genotype were used. (**F**) Effect of Dicer$^{\Delta HEL1}$ expression on small RNAs in E15.5 embryos and in ESCs. Each MA plot shows results of small RNA-seq analysis of a *Dicer*$^{\Delta HEL1/\Delta HEL1}$ sample compared with the normal control (wild-type siblings or the parental ESC line). Each colored point represents a genomic region (cluster) producing 21-23 nt RNAs. Clusters were identified and categorized by a previously developed algorithm [4]. Cluster "expression" is defined as a fraction of small RNA reads mapping to it per million 21-23 nt small RNAs (RPM). (**G**) Relative changes of significantly differentially expressed miRNAs in *Dicer*$^{\Delta HEL1/\Delta HEL1}$ E15.5 embryos (shown as colored triangles, other miRNAs are depicted as grey circles) correlate with changes of these miRNAs in *Dicer*$^{\Delta HEL1/\Delta HEL1}$ ESCs. Axes depict log_2FC. (**H**) Unique 3p passenger strand bias in *Dicer*$^{\Delta HEL1/\Delta HEL1}$ samples (upper two MA plots) or embryonic stem cell mutants (lower two MA plots) is reproducible when using a mirGeneDB miRNA annotation. MA plots depict relative changes of dominant miRNAs (left) and passenger strands (miRNA*, right) in *Dicer*$^{\Delta HEL1/\Delta HEL1}$ mutants. 5p and 3p origins of significantly changed miRNAs or miRNA*s are distinguished by color and triangle orientation as depicted. (**I**) Relative changes of dominant miRNAs and their passenger strands in *Dicer*$^{\Delta HEL1/\Delta HEL1}$ ESCs. Each triangle depicts the strand (5p or 3p) of the dominant miRNA, its position corresponds to relative changes of the dominant miRNA (x-axis) and its corresponding miRNA* (y-axis). Deep color indicates significantly dysregulated miRNAs.

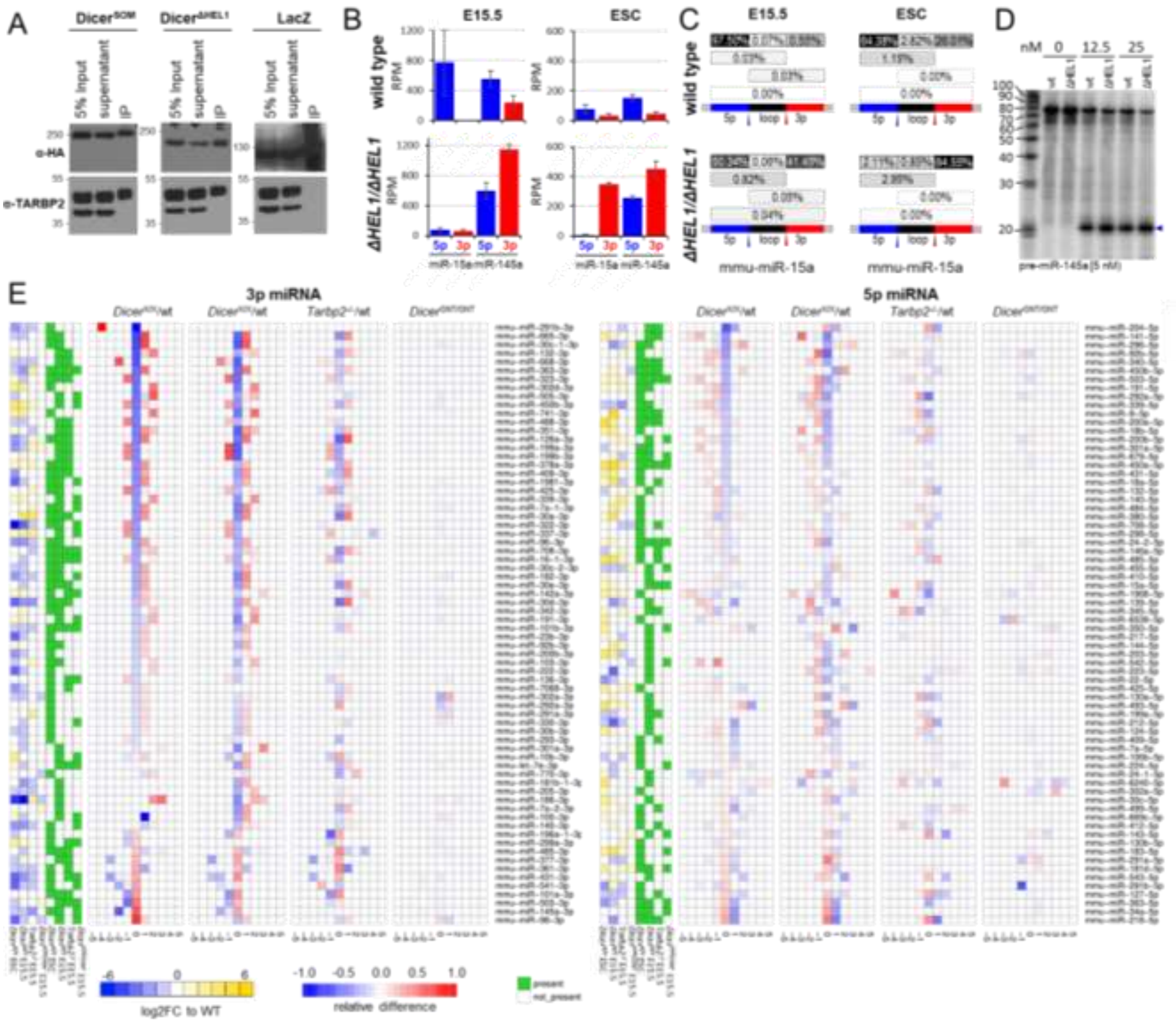

Figure S3 miRNome dysregulation in *Dicer*$^{\Delta HEL1/\Delta HEL1}$ mouse mutants. Related to Figure 3. (**A**) TARBP2 binds Dicer$^{\Delta HEL1}$. The western blots show TARBP2 presence in immunoprecipitates of Dicer^{SOM} and Dicer$^{\Delta HEL1}$ isoforms. HA-tagged Dicer^{SOM} or Dicer$^{\Delta HEL1}$ transiently expressed in NIH 3T3 cells were immunoprecipitated with α–HA antibody, and were analyzed by western blotting. The lower band in bottom western blots is a TARBP2 isoform, which does not interact with Dicer. (**B**) Expression of miR-15a and miR-145a miRNAs in *Dicer*$^{\Delta HEL1/\Delta HEL1}$ and *Tarbp2*$^{-/-}$ mutants. Expression is shown in reads per million (RPM) of 19-25 nt RNA fragments. Error bars = SD. (**C**) Products of asymmetric cleavage of pre-miR-15a are detectable in RNA-seq data from E15.5 and ESC samples. The blue-black-red lines at the bottom of each panel represent genomic pre-miRNA sequence with 5p miRNA, loop and 3p miRNA. Dicer cleavage positions are depicted by blue (3' end of 5p miRNA) and red (5' end of 3p miRNA) arrowheads. Above are shown RNA fragments corresponding to pre-miRNA, mature miRNAs, the loop, and fragments cleaved only at the 3' of 5p miRNA or 5' of 3p miRNA. Numbers correspond to percentages observed in RNA-seq data from ESCs. (**D**) miR-145a *in vitro* cleavage assay. 5 nM of *in vitro* synthesized P^{32} 5'-end labeled pre-miRNA were incubated with indicated concentrations of recombinant Dicer variants at 37°C for 60 minutes, resolved by PAGE, and visualized by phosphorimaging. Blue and red arrowheads point to products corresponding to cleavage sites giving rise to 5p and 3p miRNA, respectively. (**E**) Cleavage fidelity in *Dicer*$^{\Delta HEL1/\Delta HEL1}$ mouse mutants. Heatmaps depict analysis of cleavage sites at the 5' end of 3p miRNAs (left heatmap) and 3' end of 5p miRNAs (right heatmap) in 50 most affected miRNAs among all miRNAs (>100 DESeq RPMs) in Dicer$^{\Delta HEL1/\Delta HEL1}$ E15.5 embryos and ESCs. At the center is the annotated cleavage site. Each column of squares represents one nucleotide from the cleavage site in direction into the mature miRNA (to the right) or upstream of it (to the left). Red-blue colors indicate relative changes in a miRNA cleavage site relative to the wild type sample.

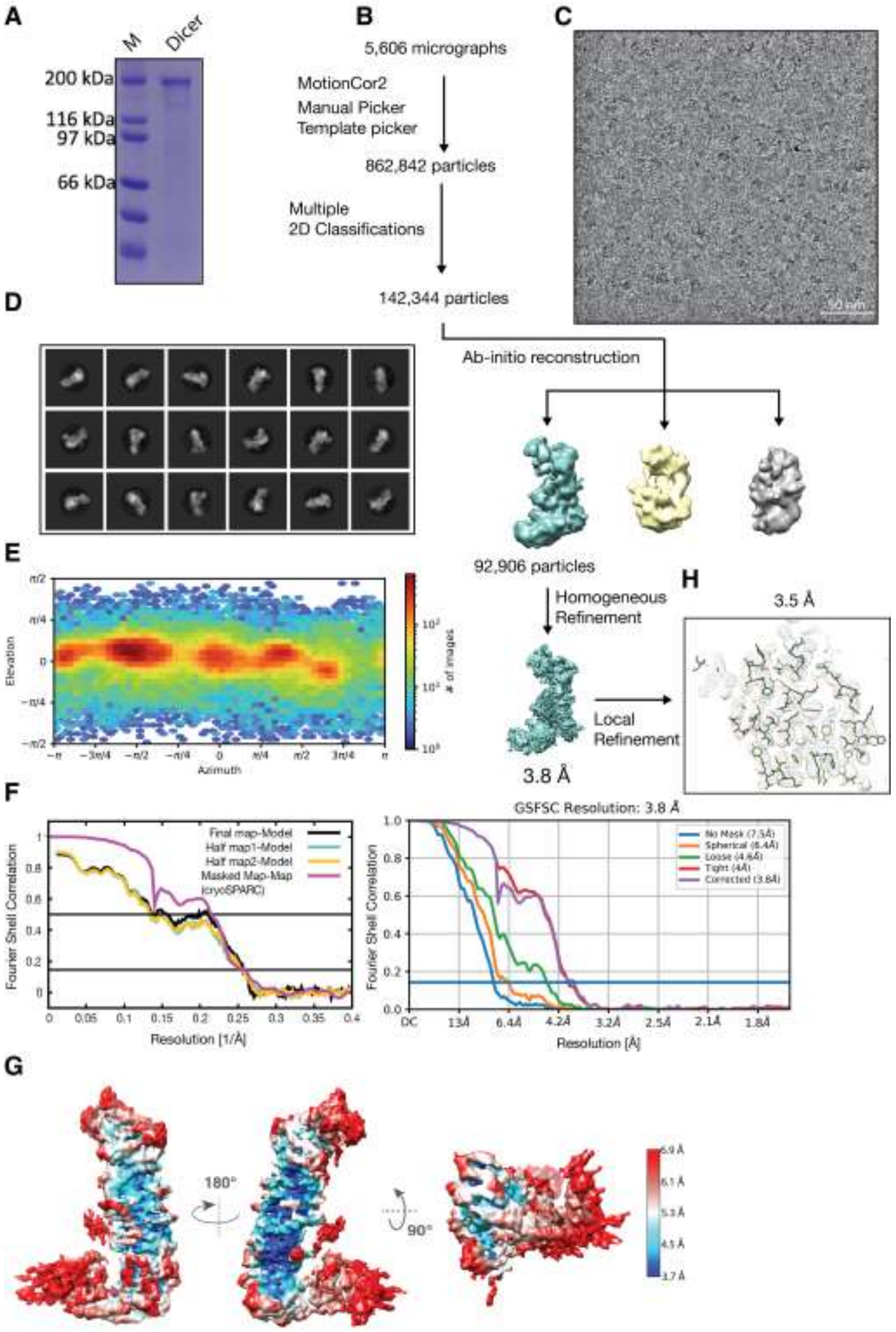

Figure S4 **Purification and cryo-EM analyses of apo-Dicer.** Related to Figure 4B (**A**) SDS-page analysis of apo-Dicer. (**B**) Outline of the image processing steps used to obtain the 3.8-Å-resolution cryo-EM reconstruction of apo-Dicer. 3D classes with no density of the helicase domain (due to inherent flexibility) were not used for the final reconstruction. (**C**) Representative Cryo-EM micrograph of apo-Dicer. (**D**) Gallery of reference-free 2D class averages. (**E**) Heat map for distribution of particles for the final 3D reconstruction. (**F**) Final map FSC (magenta) and map-to-model FSC for the full map (black), half map 1 (cyan), half map 2 (gold) curves (left). FSC curves and resolutions calculated in cryoSPARC during final refinement before and after applying soft masks (right). (**G**) Local resolution map of the final 3D reconstruction. (**H**) cryo-EM map from local refinement and fitting of coordinates.

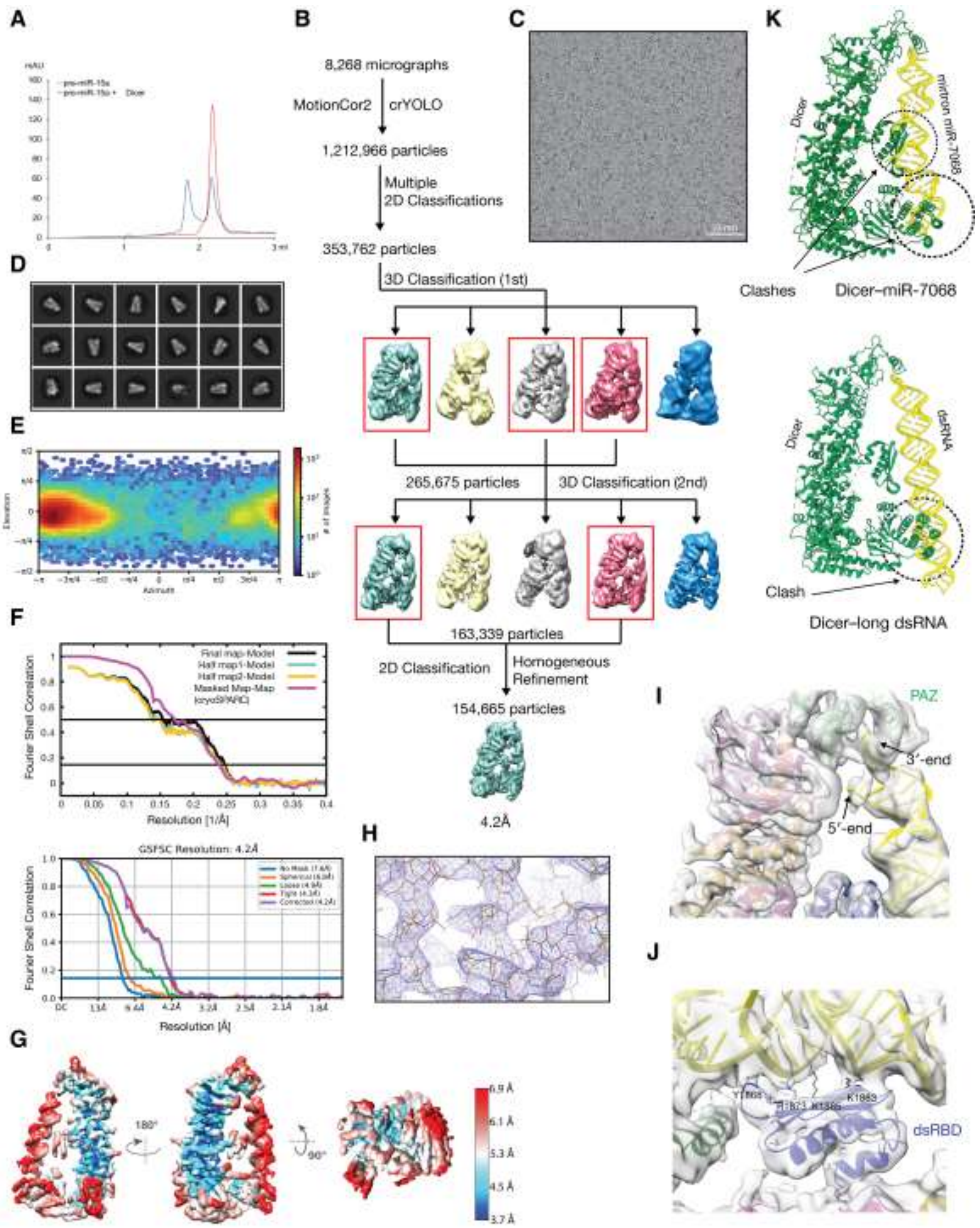

Figure S5 **Purification and cryo-EM analyses of Dicer–pre-miR-15a complex.** Related to Figure 4C (**A**) Gel filtration analysis of Dicer–pre-miR-15a complex. (**B**) Outline of the image processing steps used to obtain the 4.2-Å-resolution cryo-EM reconstruction of the Dicer–pre-miR-15a complex. (**C**) Representative cryo-EM micrograph of the Dicer–pre-miR-15a complex. (**D**) Gallery of reference-free 2D class averages. (**E**) Heat map for distribution of particles for the final 3D reconstruction. (**F**) Final map FSC (magenta) and map-to-model FSC for the full map (black), half map 1 (cyan), half map 2 (gold) curves (top). FSC curves and resolutions calculated in cryoSPARC during final refinement before and after applying soft masks (bottom) (**G**) Local resolution map of the final 3D reconstruction. (**H**) cryo-EM map and fitting of coordinates. (**I**) A close-up of the 3' end of the pre-miR-15a substrate recognition by the PAZ domain of Dicer. (**J**) A close-up of Dicer dsRBD–pre-miR-15a interface in the pre-cleavage state (putative interacting residues are indicated). (**K**) Dicer in the pre-cleavage state cannot optimally bind long mirtrons or dsRNA due to steric hindrance (indicated by arrows). The models are build based of the cryo-EM structure of Dicer–pre-miR-15a complex, in which pre-miR-15a was replaced by miR-7068 (top) or a 42-bp dsRNA (bottom).

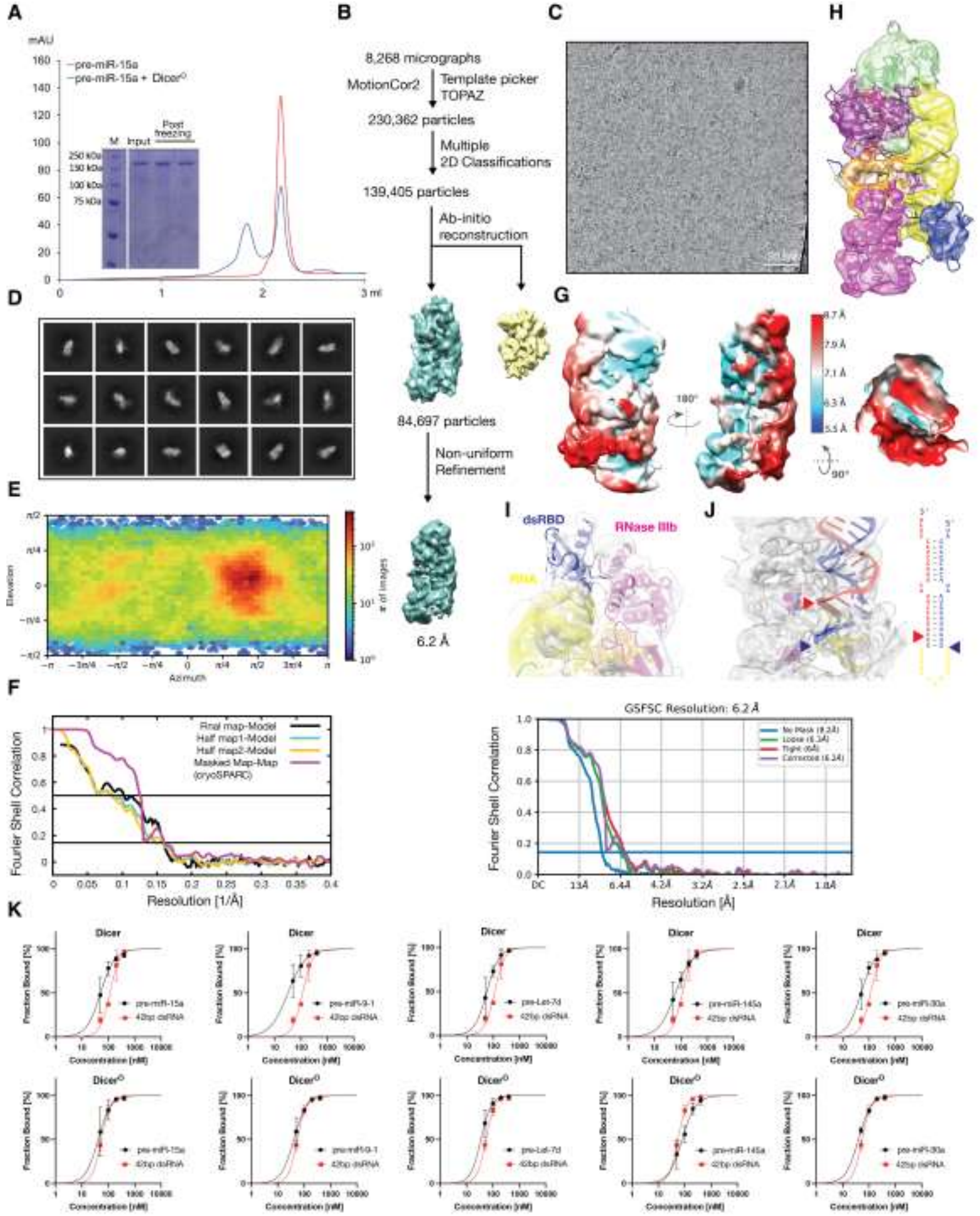

Figure S6 **Reconstitution and cryo-EM analyses of Dicer^{O}–pre-miR-15a complex.** Related to Figure 5. (**A**) Gel filtration and SDS analyses of Dicer^{O}–pre-miR-15a complex. (**B**) Outline of the image processing steps used to obtain the 6.2-Å-resolution cryo-EM reconstruction of the Dicer^{O}–pre-miR-15a complex. (**C**) Representative cryo-EM micrograph. (**D**) Gallery of reference-free 2D class averages. I Heat map for distribution of particles for the final 3D reconstruction. (**F**) Final map FSC (magenta) and map-to-model FSC for the full map (black), half map 1 (cyan), half map 2 (gold) curves (left). FSC curves and resolutions calculated in cryoSPARC during final refinement before and after applying soft masks (right). (**G**) Local resolution map of the final 3D reconstruction. (**H**) Structural models superposed to segmented cryo-EM densities and for the PAZ (green), Platform (violet), Ruler (red), RNase IIIa (orange), and RNase IIIb (magenta) domains of Dicer^{O} and pre-miR-15a (yellow) are shown. (**I**) A close-up of dsRBD/RNase IIIb–RNA interface. (**J**) A close-up of putative RNA cleavage sites (indicated by arrows) and their alignment with Dicer's catalytic sites (in magenta). (**K**) Quantification of electrophoretic mobility shift assays of Dicer isoforms with different mi-RNA precursors and a 42bp perfect hairpin. Data points, mean ± SD (n=2-3).

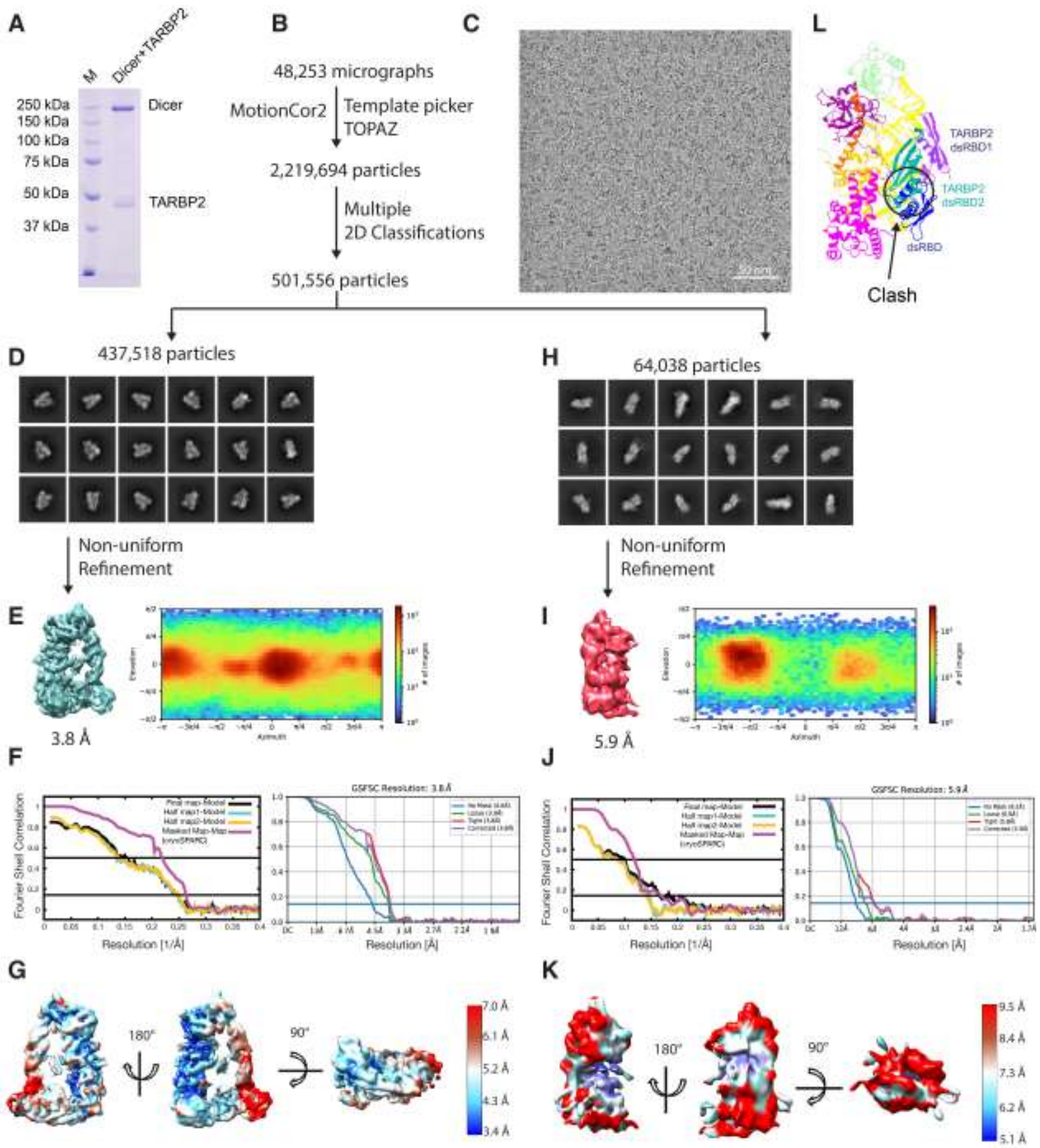

Figure S7 Reconstitution and cryo-EM analysis of Dicer–pre-miR-15a–TARBP2 complex. Related to Figure 6. (**A**) SDS-page analysis of Dicer–pre-miR-15a–TARBP2 complex. (**B**) Outline of the image processing steps of the Dicer–pre-miR-15a–TARBP2 complex. (**C**) Representative cryo-EM micrograph of the ternary complex. (**D**) Gallery of 2D class averages for the pre-cleavage state. (**E**) 3.8-Å-resolution cryo-EM reconstruction of the pre-cleavage state (left). Heat map for distribution of particles for the final 3D reconstruction (right) (**F**) Final map FSC (magenta) and map-to-model FSC for the full map (black), half map 1 (cyan), half map 2 (gold) curves (left). FSC curves and resolutions calculated in cryoSPARC during final refinement before and after applying soft masks (right). (**G**) Local resolution map of the final 3D reconstruction. (**H**) Gallery of 2D class averages for the cleavage state. (**I**) 5.9-Å-resolution cryo-EM reconstruction of the cleavage state (left). Heat map for distribution of particles for the final 3D reconstruction (right). (**J**) Final map FSC (magenta) and map-to-model FSC for the full map (black), half map 1 (cyan), half map 2 (gold) curves (left). FSC curves and resolutions calculated in cryoSPARC during final refinement before and after applying soft masks (right). (**K**) Local resolution map of the final 3D reconstruction. (**L**) Superimposition of pre-miR-15a with TARBP2 dsRBD12 from the pre-cleavage state on the Dicer–pre-miR-15a–TARBP2 structure in the cleavage state.

SUPPLEMENTARY TABLES

Table S2 Expression of host genes of most upregulated mirtrons in ESCs. Related to Figure 2.

host gene	host gene id	mRNA baseMean	log2FC	pvalue	padj	mirtron
Cherp	ENSMUSG00000052488.7	617.8	0.845	0.004	0.377	mmu-miR-7068
Dbn1	ENSMUSG00000034675.17	52.0	-0.304	0.542	1.000	mmu-miR-6944
Arap3	ENSMUSG00000024451.8	10.4	0.614	0.358	1.000	mmu-miR-6981
Fbrs	ENSMUSG00000042423.9	197.3	0.219	0.520	1.000	mmu-miR-7060
Dennd6b	ENSMUSG00000015377.9	163.9	0.105	0.791	1.000	mmu-miR-6958
Gfra4	ENSMUSG00000027316.15	11.4	-0.720	0.280	1.000	mmu-miR-6973b
Fbxw9	ENSMUSG00000008167.14	711.2	0.223	0.536	1.000	mmu-miR-7070
Hspg2	ENSMUSG00000028763.17	2418.0	-0.348	0.264	1.000	mmu-miR-7018
Nav1	ENSMUSG00000009418.15	451.2	0.807	0.006	0.499	mmu-miR-1231
Atp2b4	ENSMUSG00000026463.17	275.5	-1.178	0.003	0.351	mmu-miR-6903
Arhgef17	ENSMUSG00000032875.8	301.1	-0.549	0.085	1.000	mmu-miR-3102
Etfb	ENSMUSG00000004610.4	666.6	-0.477	0.121	1.000	mmu-miR-7051
Ptprs	ENSMUSG00000013236.17	602.1	0.012	0.971	1.000	mmu-miR-6977
Rrp1	ENSMUSG00000061032.9	1706.2	-0.101	0.757	1.000	mmu-miR-6907
Myh3	ENSMUSG00000020908.14	27.6	-0.389	0.473	1.000	mmu-miR-6923
Baiap3	ENSMUSG00000047507.12	4.4	-0.270	0.733	1.000	mmu-miR-3547
Ciao3	ENSMUSG00000002280.10	367.9	-0.313	0.296	1.000	mmu-miR-6966
Hip1r	ENSMUSG00000000915.15	189.2	0.241	0.499	1.000	mmu-miR-7032
Farsa	ENSMUSG00000003808.18	1774.8	0.373	0.228	1.000	mmu-miR-7069
Mst1	ENSMUSG00000032591.15	93.0	-0.334	0.458	1.000	mmu-miR-7088
Ap2a2	ENSMUSG00000002957.11	2406.3	0.011	0.972	1.000	mmu-miR-7063
Dnase1l1	ENSMUSG00000019088.13	27.5	-0.661	0.207	1.000	mmu-miR-7091

Table S3 Asymmetric cleavage of miRNAs. Related to Figure 3. Shown are frequencies of specific miRNA fragments in RNA-sequencing data from *Dicer*$^{\Delta HEL1/\Delta HEL1}$ ESCs. The fragment miR-5p+loop is produced by asymmetric cleave at the 5' end of 3p miRNA.

miRNA	miR-5p	loop	miR-3p	**miR-5p+loop**	miR-3p+loop
mmu-miR-7041	0.0000	0.0000	0.6667	**0.3333**	0.0000
mmu-miR-7067	0.0000	0.0000	0.3333	**0.3333**	0.0000
mmu-miR-667	0.5358	0.0037	0.0838	**0.3322**	0.0000
mmu-miR-31	0.5807	0.0000	0.0519	**0.2948**	0.0031
mmu-miR-29c	0.1667	0.0000	0.1667	**0.2222**	0.0000
mmu-miR-101b	0.0000	0.0000	0.8350	**0.1456**	0.0012
mmu-miR-465b	0.5317	0.0000	0.3394	**0.1106**	0.0000
mmu-miR-465b	0.5317	0.0000	0.3394	**0.1106**	0.0000
mmu-miR-539	0.2650	0.2222	0.0513	**0.0769**	0.0256
mmu-miR-154	0.2100	0.0284	0.6601	**0.0664**	0.0002
mmu-miR-3070	0.2405	0.0000	0.6881	**0.0476**	0.0000
mmu-miR-465c	0.5198	0.0000	0.4097	**0.0407**	0.0000
mmu-miR-465c	0.5198	0.0000	0.4097	**0.0407**	0.0000
mmu-miR-142a	0.3436	0.0082	0.3154	**0.0402**	0.0000
mmu-miR-377	0.5106	0.0000	0.4012	**0.0359**	0.0000
mmu-miR-367	0.0000	0.0827	0.7694	**0.0351**	0.0000
mmu-miR-878	0.6171	0.0000	0.2904	**0.0334**	0.0000
mmu-miR-293	0.2776	0.0000	0.6612	**0.0291**	0.0001
**mmu-miR-15a**	**0.0193**	**0.0078**	**0.8588**	**0.0270**	**0.0000**
mmu-miR-141	0.1689	0.1947	0.3683	**0.0264**	0.0034
mmu-miR-324	0.0620	0.0361	0.8431	**0.0250**	0.0000
mmu-miR-883b	0.5185	0.0000	0.4577	**0.0238**	0.0000
mmu-miR-376b	0.0291	0.0173	0.9008	**0.0235**	0.0000
mmu-miR-485	0.3695	0.0001	0.5528	**0.0214**	0.0146
mmu-miR-20b	0.9475	0.0006	0.0089	**0.0199**	0.0004
mmu-miR-743b	0.0394	0.0000	0.9235	**0.0185**	0.0000
mmu-miR-665	0.0400	0.0000	0.8164	**0.0176**	0.0820
mmu-miR-679	0.7761	0.0000	0.0568	**0.0167**	0.0000
mmu-miR-465a	0.0921	0.0000	0.8556	**0.0143**	0.0057
mmu-miR-181c	0.0812	0.0000	0.8520	**0.0140**	0.0000
mmu-miR-188	0.9352	0.0139	0.0000	**0.0139**	0.0000
mmu-miR-411	0.7924	0.0023	0.1631	**0.0139**	0.0000
mmu-miR-93	0.9053	0.0019	0.0396	**0.0135**	0.0000
mmu-miR-301a	0.5232	0.0067	0.1228	**0.0133**	0.0000
mmu-miR-362	0.4786	0.0000	0.4550	**0.0130**	0.0000
mmu-miR-341	0.0241	0.0000	0.8388	**0.0121**	0.0003
mmu-miR-380	0.3384	0.0000	0.5523	**0.0115**	0.0000
mmu-miR-211	0.8401	0.0000	0.1078	**0.0109**	0.0021
mmu-miR-29a	0.0085	0.0000	0.9267	**0.0096**	0.0000
mmu-miR-677	0.2121	0.0000	0.0004	**0.0089**	0.0025

Table S4 Cryo-EM data collection and refinement statistics. Related to Figure 4-6.

**Instrument**					
Microscope	FEI Titan Krios				
**Data collection**					
**Sample**	**Dicer**	**Dicer–RNA**	**Dicer^{O}–RNA**	**Dicer–RNA-TARBP2 (pre-cleavage)**	**Dicer–RNA-TARBP2 (cleavage)**
EMDB accession number	EMD-14387	EMD-14383	EMDB-14384	EMDB-14856	EMDB-14854
PDB accession number	7YZ4	7YYM	7YYN	7ZPK	7ZPI
Voltage (kV)	300	300	300	300	300
Detector (counting mode)	Gatan K2	Gatan K2	Gatan K2	Gatan K3	Gatan K3
Symmetry	C1	C1	C1	C1	C1
Electron dose ($e^-/Å^2$)	55.0	55.0	55.0	60.198	60.198
Defocus range (µm)	-0.8 to -3.5	-1 to -3.5	-1 to -3.5	-0.8 to -3.5	-0.8 to -3.5
Pixel size (Å)	0.828	0.828	0.828	0.835	0.835
Movies collected	6,354	16,601	9,956	48,253	48,253
Initial particles	862,842	1,212,966	230,362	2,219,694	2,219,694
Final particles	92,906	154,665	84,697	437,518	64,038
**Model composition**					
Protein residues	1234	1281	705	1513	705
**Refinement**					
Map resolution (cryoSPARC) 0.143/0.5 (Å)	3.8/4.6	4.2/5.8	6.2/7.9	3.8/4.5	5.9/8.4
Map-to-model FSC 0.143/0.5 (Å)	3.9/7.3	4.3/6.7	6.3/9.7	4.0/6.5	6.0/9.7
Combined map resolution range (Å)	3.5 – 4.2	-	-	-	-
Map sharpening *B*-factor ($Å^2$)	87.8	134.8	451.4	189.2	520.0
**R.m.s deviations**					
Bond lengths (Å)	0.004	0.004	0.013	0.005	0.004
Bond angles (°)	0.780	0.798	1.917	1.089	0.290
**Validation**					
MolProbity score	1.12	1.07	0.78	1.15	0.70
Clashscore	1.46	1.57	0.92	2.24	0.61
Poor rotamers (%)	0.92	0.18	0.64	0.68	0.64
**Ramachandran plot**					
Favored (%)	96.44	97.07	98.70	97.18	97.44
Allowed (%)	3.56	2.93	1.30	2.82	2.56
Disallowed (%)	0.00	0.00	0.00	0.00	0.00

Table S5 RNA-seq libraries used in the study. Related to Figure 1-3 and STAR Methods (Method Details, section Bioinformatics analyses).

stage	type	genotype	library name	note
ESC	small RNA	*Dicer$^{wt/wt}$*	s_ESC_WT+MosIR_RS7.1	transfected with MosIR
ESC	small RNA	*Dicer$^{wt/wt}$*	s_ESC_WT+MosIR_RS7.2	transfected with MosIR
ESC	small RNA	*Dicer$^{wt/wt}$*	s_ESC_WT+MosIR_RS7.3	transfected with MosIR
ESC	small RNA	*Dicer$^{\Delta HEL1/\Delta HEL1}$*	s_ESC_XHOM+MosIR_RS10.1	transfected with MosIR
ESC	small RNA	*Dicer$^{\Delta HEL1/\Delta HEL1}$*	s_ESC_XHOM+MosIR_RS10.2	transfected with MosIR
ESC	small RNA	*Dicer$^{\Delta HEL1/\Delta HEL1}$*	s_ESC_XHOM+MosIR_RS10.3	transfected with MosIR
E15.5	small RNA	*Dicer$^{wt/wt}$*	s_E15.5_WT_1	
E15.5	small RNA	*Dicer$^{wt/wt}$*	s_E15.5_WT_6	
E15.5	small RNA	*Dicer$^{wt/wt}$*	s_E15.5_WT_8B	
E15.5	small RNA	*Dicer$^{\Delta HEL1/\Delta HEL1}$*	s_E15.5_XHOM_10B_r2	
E15.5	small RNA	*Dicer$^{\Delta HEL1/\Delta HEL1}$*	s_E15.5_XHOM_2	
E15.5	small RNA	*Dicer$^{\Delta HEL1/\Delta HEL1}$*	s_E15.5_XHOM_3B	
E15.5	small RNA	*Dicer$^{\Delta HEL1/\Delta HEL1}$*	s_E15.5_XHOM_4	
E15.5	small RNA	*Dicer$^{\Delta HEL1/\Delta HEL1}$*	s_E15.5_XHOM_7B	
E15.5	small RNA	*Dicer$^{wt/wt}$*	s_E15.5_WT_11	
E15.5	small RNA	*Dicer$^{wt/wt}$*	s_E15.5_WT_14	
E15.5	small RNA	*Dicer$^{wt/wt}$*	s_E15.5_WT_16	
E15.5	small RNA	*Dicer$^{GNT/GNT}$*	s_E15.5_GNTHOM_3	
E15.5	small RNA	*Dicer$^{GNT/GNT}$*	s_E15.5_GNTHOM_4	
E15.5	small RNA	*Dicer$^{GNT/GNT}$*	s_E15.5_GNTHOM_9	
E15.5	small RNA	*Tarbp2$^{+/+}$*	SRS2781156 B6T2-65Tarbp2_WT_1	PRJNA423238 SRP127346
E15.5	small RNA	*Tarbp2$^{+/+}$*	SRS2781151 B6T2-65Tarbp2_WT_2	PRJNA423238 SRP127346
E15.5	small RNA	*Tarbp2$^{+/+}$*	SRS2781150B6T2-65Tarbp2_WT_3	PRJNA423238 SRP127346
E15.5	small RNA	*Tarbp2$^{-/-}$*	SRS2781155 B6T2-54Tarbp2_Mut_1	PRJNA423238 SRP127346
E15.5	small RNA	*Tarbp2$^{-/-}$*	SRS2781154 B6T2-60Tarbp2_Mut_2	PRJNA423238 SRP127346
E15.5	small RNA	*Tarbp2$^{-/-}$*	SRS2781157 B6T2-90Tarbp2_Mut_3	PRJNA423238 SRP127346

References

1. Taborska, E., Pasulka, J., Malik, R., Horvat, F., Jenickova, I., Jelic Matosevic, Z., and Svoboda, P. (2019). Restricted and non-essential redundancy of RNAi and piRNA pathways in mouse oocytes. PLoS Genet *15*, e1008261. 10.1371/journal.pgen.1008261.
2. Fromm, B., Hoye, E., Domanska, D., Zhong, X., Aparicio-Puerta, E., Ovchinnikov, V., Umu, S.U., Chabot, P.J., Kang, W., Aslanzadeh, M., et al. (2022). MirGeneDB 2.1: toward a complete sampling of all major animal phyla. Nucleic Acids Res *50*, D204-D210. 10.1093/nar/gkab1101.
3. Ladewig, E., Okamura, K., Flynt, A.S., Westholm, J.O., and Lai, E.C. (2012). Discovery of hundreds of mirtrons in mouse and human small RNA data. Genome Res *22*, 1634-1645. 10.1101/gr.133553.111.
4. Flemr, M., Malik, R., Franke, V., Nejepinska, J., Sedlacek, R., Vlahovicek, K., and Svoboda, P. (2013). A retrotransposon-driven dicer isoform directs endogenous small interfering RNA production in mouse oocytes. Cell *155*, 807-816. 10.1016/j.cell.2013.10.001.
